# Internal Energy Dependence of the Pyrrole Dimer Cation Structures Formed in a Supersonic Plasma Expansion: Charge-Resonance and Hydrogen-Bonded Isomers

**DOI:** 10.1021/acs.jpca.4c01834

**Published:** 2024-05-13

**Authors:** Dashjargal Arildii, Yoshiteru Matsumoto, Otto Dopfer

**Affiliations:** † Institut für Optik und Atomare Physik, Technische Universität Berlin, Hardenbergstrasse 36, 10623 Berlin, Germany; ‡ Department of Chemistry, Faculty of Science, Shizuoka University, 836 Ohya, Suruga-ku, Shizuoka 422-8529, Japan; § International Research Frontiers Initiative, Tokyo Institute of Technology, 4259 Nagatsuta-cho, Midori-ku, Yokohama 226-8503, Japan

## Abstract

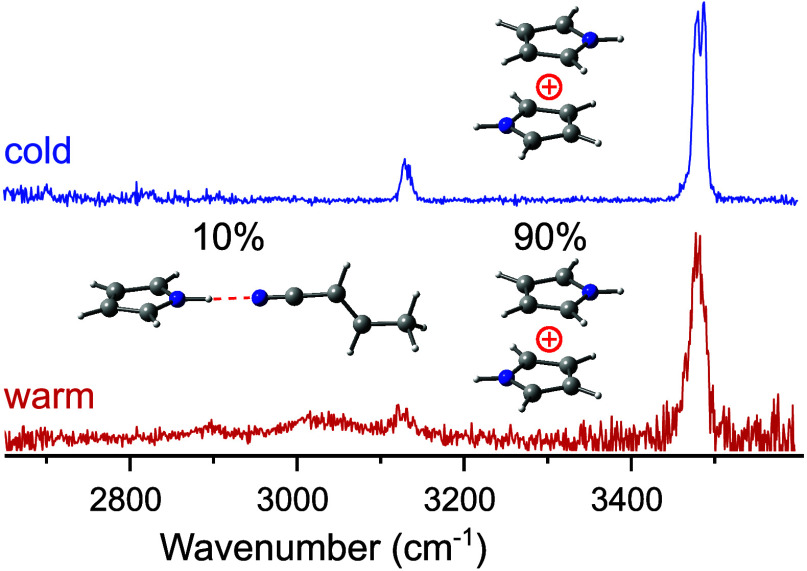

The structures of the pyrrole dimer cation (Py_2_^+^) formed in an electron-ionization-driven supersonic plasma expansion of Py seeded in Ar or N_2_ are probed as a function of its internal energy by infrared photodissociation (IRPD) spectroscopy in a tandem mass spectrometer. The IRPD spectra recorded in the CH and NH stretch ranges are analyzed by dispersion-corrected density functional theory (DFT) calculations at the B3LYP-D3/aug-cc-pVTZ level. The spectra of the cold Ar/N_2_-tagged Py_2_^+^ clusters, Py_2_^+^L_*n*_ (*n* = 1–5 for Ar, *n* = 1 for N_2_), indicate the exclusive formation of the most stable antiparallel π-stacked Py_2_^+^ structure under cold conditions, which is stabilized by charge–resonance interaction. The bare Py_2_^+^ dimers produced in the ion source have higher internal energy, and the observation of additional transitions in their IRPD spectra suggests a minor population of less stable hydrogen-bonded isomers composed of heterocyclic Py/Py^+^ structures formed after intramolecular H atom transfer and ring opening. These intermolecular isomers differ from the chemically bonded structures proposed earlier in the analysis of IRPD spectra of Py_2_^+^ generated by VUV ionization of neutral Py_*n*_ clusters.

## 1 Introduction

Intermolecular interactions between aromatic rings are important in chemical and biological recognition and determine the three-dimensional structure and function of biological supramolecular architectures such as proteins and DNA.^1,2^ Radical ions of arenes play a crucial role in chemical reaction mechanisms, charge transport of organic semiconductors, and radiation damage.^3−6^ In addition to π hydrogen bonding (π H-bonding), cation/anion−π, and π–π stacking interactions,^7−11^ charge–resonance (CR) is a very strong fundamental force in charged arene dimers.^12^ Such singly charged dimer cations are stabilized by forming a π-stacked sandwich structure and sharing the positive excess charge between the two aromatic π-electron systems.^12^ The charge distribution between the two units depends on their ionization energy (IE), and the unit with lower IE carries a higher positive partial charge. Due to the CR interaction between the monomers, the splitting between the two electronic states originating from the symmetric and antisymmetric linear combinations of the wave functions described for homodimers as
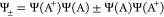
results in an intense CR transition.^12,13^ Here, the bound symmetric Ψ_+_ ground state is stabilized by the CR, while the destabilized antisymmetric Ψ_–_ state is repulsive. The coupling between these two electronic states is strongest for A_2_^+^ homodimers and reduced in AB^+^ heterodimers by an amount depending on the difference in the IEs of A and B.^14,15^

CR interactions were first detected in the condensed phase by electron spin resonance^16−18^ and optical absorption spectroscopy.^19−22^ However, these experiments suffer from environmental perturbations, such as solvent, matrix, and counterions. In the gas phase, the environmental influence is eliminated, and binding energies of several aromatic homo- and heterodimers were measured by mass spectrometric techniques.^23−25^ Moreover, the analysis of the CR transitions measured for the aromatic homodimers of benzene,^26−29^ naphthalene,^30,31^ toluene,^32^ pyrene,^33^ and several heterodimers^32,34,35^ has proven that the splitting between the two electronic states (Ψ_±_) depends on the size and IE of the two monomer units. Nonetheless, the electronic CR bands observed by optical absorption in the spectral range around 1 eV are too broad even in the gas phase (because it is a transition into a repulsive state) to precisely estimate the positive charge distribution of the monomers.

Previously, we demonstrated that vibrational infrared (IR) spectroscopy in the ground electronic state (Ψ_+_) provides a sensitive probe of the charge distribution in isolated aromatic A_2_^+^ and AB^+^ dimers.^14^ The employed technique of IR photodissociation (IRPD) of mass-selected ions generated in a supersonic plasma expansion was first applied to the prototypical case of the pyrrole dimer cation (Py_2_^+^) and its clusters and related heterodimers and is based on the high sensitivity of certain vibrational frequencies of Py on its partial charge. Py (C_4_H_5_N) is a five-membered planar heterocyclic arene with *C*_2*v*_ symmetry and a suitable model system to study the CR interaction by IRPD for the following reasons.^14^ First, Py_2_^+^ exhibits a π-stacked structure with a strong CR interaction due to its small size and strong overlap of the two π-electron systems. Moreover, Py has a single isolated, uncoupled, and strongly IR active NH stretch oscillator, whose frequency is rather sensitive to the charge state of Py^(+)^ (ν_NH_ = 3531 and 3447 cm^–1^ for *q* = 0 and 1*e*).^36,37^ This is different from bare aromatic hydrocarbon molecules, such as benzene and polycyclic aromatic hydrocarbons, whose CH stretch frequencies are less sensitive to charge and have weaker IR oscillator strength in the cation ground state. Moreover, the CH stretch range in these hydrocarbons is often complicated by Fermi resonances (e.g., C_6_H_6_ and C_6_H_4_Cl_2_^+^) and/or the Jahn–Teller effect (e.g., C_6_H_6_^+^).^27,34,38−42^

**Figure 1:**
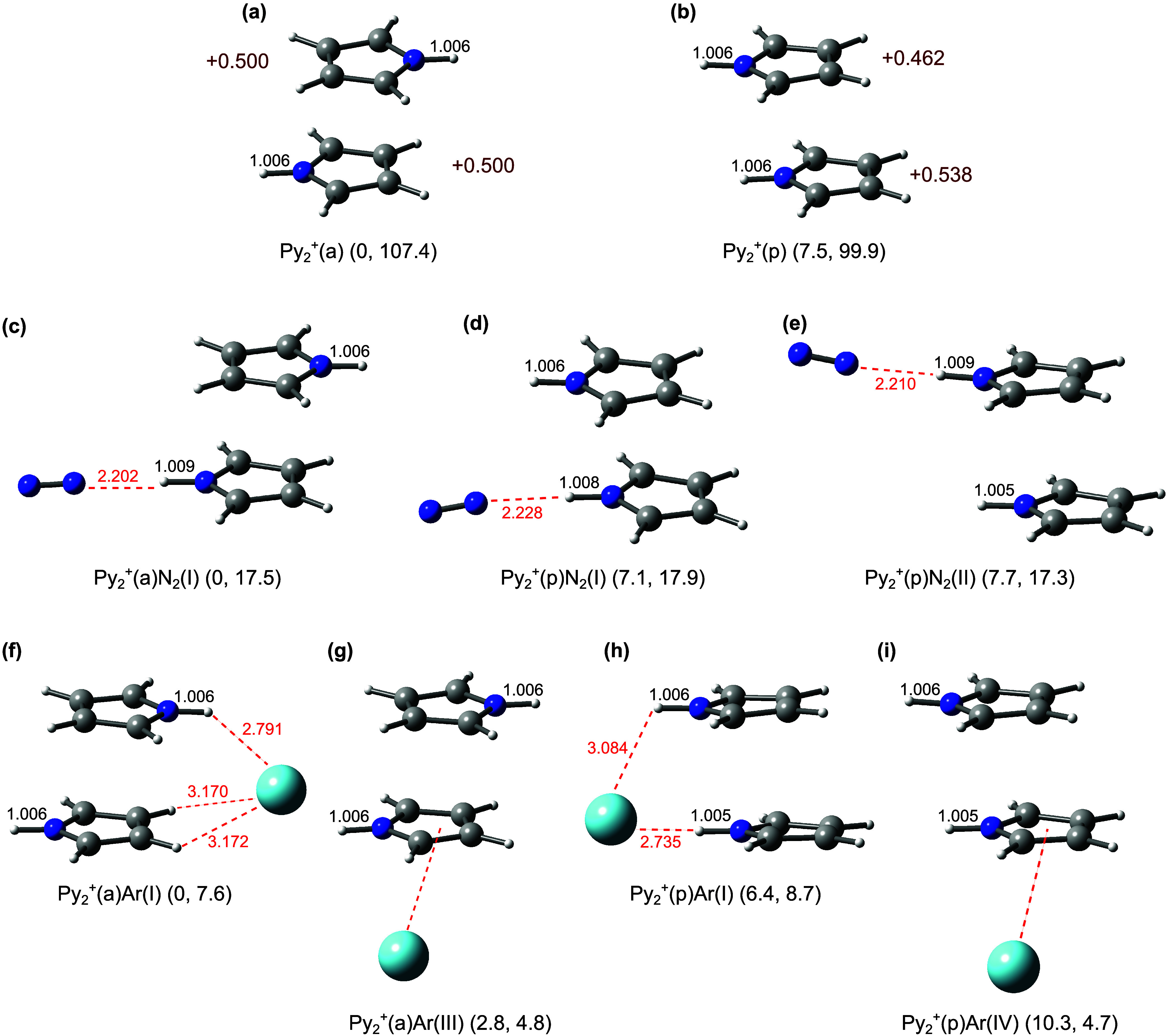
Optimized structures of (a, b) Py_2_^+^(a/p), (c–e) Py_2_^+^(a/p)N_2_, and (f–i) Py_2_^+^(a/p)Ar isomers calculated at the B3LYP-D3/aug-cc-pVTZ level. Selected intra- and intermolecular lengths (in Å) are indicated in black and red colors, respectively. Values in dark red indicate molecular charges (in units of *e*). The energies in parentheses correspond to relative energies and dissociation energies of the most weakly bonded ligand (*E*_0_ and *D*_0_ in kJ mol^–1^). Further higher-energy isomers of Py_2_^+^(a/p)N_2_ and Py_2_^+^(a/p)Ar are available in Figures S2 and S4, respectively.

Our previous study established a nearly linear correlation between the positive partial charge of the Py unit and the redshift in its free NH stretch frequency (ν_NH_^f^), illustrating that IR chromophores are useful probes of the CR interaction in dimer cations using IR spectroscopy.^14^ The Py_2_^+^ cation stabilized by CR has *q* = +0.5*e* for each Py unit, and consequently, its ν_NH_^f^ detected at 3480 cm^–1^ is midway between ν_NH_^f^ of Py and Py^+^.^14^ However, previously, only the antiparallel Py_2_^+^(a) global minimum structure, in which the NH groups of the two Py units are pointing in opposite directions, was considered (Figure 1).^14^ Py_2_^+^(a) has *C*_2*h*_ symmetry, and consequently, each Py unit has *q* = +0.5*e*. The rather high dissociation energy is calculated as *D*_0_ = 107.4 kJ mol^–1^ (B3LYP-D3/aug-cc-pVTZ). The IR spectrum computed for Py_2_^+^(a) predicts a single and intense antisymmetric NH stretch band (ν_NH_^a^) at 3500 cm^–1^ (Table 1). Due to its inversion symmetry, the symmetric NH stretch mode (ν_NH_^s^) is IR forbidden (Figure S1). However, the slightly less stable Py_2_^+^(p) isomer (Figure 1), in which the NH groups of the two Py units point toward the same direction in a parallel fashion, is only Δ*E*_0_ = 7.5 kJ mol^–1^, higher in energy than Py_2_^+^(a). Because Py_2_^+^(p) has *C*_1_ symmetry, the charge distribution on the two Py units is no longer equal, with *q* = +0.462 and +0.538*e*. As a result, the predicted ν_NH_^a,s^ modes of Py_2_^+^(p) are different from those of Py_2_^+^(a). In particular, both normal modes predicted at ν_NH_^s^ = 3510 and ν_NH_^a^ = 3499 cm^–1^ with a splitting of 11 cm^–1^ have comparable IR oscillator strengths (120 and 131 km mol^–1^, Table 1). The reported IRPD spectrum of Py_2_^+^ exhibits a single unresolved ν_NH_ band at 3480 cm^–1^ with a large width of 30 cm^–1^ because of its high internal energy, resulting from its large binding energy.^14^ As this width exceeds the predicted splitting of 11 cm^–1^ of Py_2_^+^(p), the presence of this less stable isomer in the molecular beam cannot readily be ruled out from the measured IRPD spectrum. To clarify the Py_2_^+^(a/p) isomer population, we analyze herein colder Py_2_^+^ dimers generated by tagging with Ar and N_2_ ligands. Their much lower binding energies result in higher-resolution IRPD spectra.

**Table 1:** Positions and Suggested Vibrational Assignments of the Transitions Observed in the IRPD Spectra of Py_2_^+^, Py_2_^+^Ar, and Py_2_^+^N_2_ Compared to Frequencies of the Most Stable Isomers Calculated at the B3LYP-D3/aug-cc-pVTZ Level

	exp (cm^–1^)^a^	mode	isomer	calc (cm^–1^)^b^	mode
Py_2_^+^(hot)	A 3477 (30)	ν_NH_^f,a,s^	Py_2_^+^(a)	3500 (289)	ν_NH_^a^
				3137 (19)	ν_CH_
	C 3128 (53)	ν_CH_	Py_2_^+^(p)	3510 (120)	ν_NH_^s^
				3499 (131)	ν_NH_^a^
	D 3026 (135)	ν_NH_^b^		3139 (12)	ν_CH_
			Py_2_^+^(CC1)(I)	1878 (5376)	ν_NH_^b^
	E 2891 (51)	FR		3126 (9)	ν_CH_
				3053 (5)	ν_CH_^′^
	F 3236 (84)		Py_2_^+^(CC2)(I)	2055 (5126)	ν_NH_^b^
				3126 (10)	ν_CH_
	G 2756 (63)			2909 (4)	ν_CH_^′^
Py_2_^+^	A 3479 (19)	ν_NH_^f,a,s^	Py_2_^+^(OC1)(I)	2792 (3561)	ν_NH_^b^
				3128 (14)	ν_CH_
				3031 (5)	ν_CH2_
	C 3126 (29)	ν_CH_	Py_2_^+^(OC2)(I)	2858 (2958)	ν_NH_^b^
				3128 (15)	ν_CH_
				3022 (3)	ν_CH2_
	D 3028 (116)	ν_NH_^b^	Py_2_^+^(OC3)(I)	3503 (123)	ν_NH_^f^
				2570 (1332)	ν_NH_^b^
	E 2888 (68)	FR		3136 (5)	ν_CH_
				3043 (9)	ν_CH2_
Py_2_^+^N_2_	A 3489 (5)	ν_NH_^f^	Py_2_^+^(a)N_2_(I)	3503 (138)	ν_NH_^f^
				3447 (523)	ν_NH_^b^
				3138 (11)	ν_CH_
	B 3449 (17)	ν_NH_^b^	Py_2_^+^(p)N_2_(I)	3504 (154)	ν_NH_^f^
				3464 (338)	ν_NH_^b^
				3138 (11)	ν_CH_
	C 3133 (11)	ν_CH_	Py_2_^+^(p)N_2_(II)	3511 (108)	ν_NH_^f^
				3447 (535)	ν_NH_^b^
				3139 (10)	ν_CH_
Py_2_^+^Ar	A 3487 (8)	ν_NH_^a^	Py_2_^+^(a)Ar(I)	3502 (99)	ν_NH_^s^
				3499 (219)	ν_NH_^a^
				3139 (15)	ν_CH_
			Py_2_^+^(a)Ar(III)	3502 (7)	ν_NH_^s^
	B 3480 (13)	ν_NH_^s^		3501 (277)	ν_NH_^a^
				3138 (16)	ν_CH_
			Py_2_^+^(p)Ar(I)	3508 (183)	ν_NH_^s^
				3501 (92)	ν_NH_^a^
	C 3130 (11)	ν_CH_		3139 (11)	ν_CH_
			Py_2_^+^(p)Ar(IV)	3510 (116)	ν_NH_^s^
				3499 (131)	ν_NH_^a^
				3139 (12)	ν_CH_

a The peak positions and widths (in parentheses) are taken from the peak deconvolution.

b The peak positions are the maxima of the convolution; IR intensities are in parentheses (in km mol^–1^).

A further motivation for the present study results from a recent IRPD spectrum of Py_2_^+^ reported by Wei et al.,^43^ which differs in several aspects from our earlier report,^14^ including the production mechanism and the investigated spectral range. First, we produce Py_2_^+^ in the high-pressure region of a supersonic plasma expansion, in which a Py monomer is first ionized by electron (or chemical) ionization (EI), and subsequently, Py_2_^+^ dimers are formed by three-body collisions. In the study by Wei et al.,^43^ Py_2_^+^ dimer ions are generated by vacuum ultraviolet (VUV) ionization of neutral Py_*n*_ clusters at 118 nm in the collision-free region of a supersonic expansion. Thus, Py_2_^+^ cations may be produced by ionization of neutral Py_2_ dimers or larger Py_*n*_ clusters followed by fragmentation of Py_*n*>2_^+^ into Py_2_^+^. Second, our limited spectral range of 3100–3600 cm^–1^ covered only the two transitions at 3480 and 3130 cm^–1^ assigned to ν_NH_ and aromatic ν_CH_ modes of π-stacked Py_2_^+^(a).^14^ The Py_2_^+^ spectrum of Wei et al.^43^ recorded in the extended range of 2600–3700 cm^–1^ shows the same two bands assigned to ν_CH_ (3138 cm^–1^) and ν_NH_ (3479 cm^–1^) of Py_2_^+^(a) but exhibits two additional weaker transitions at 2896 and 3020 cm^–1^ in the range not covered previously. These peaks were assigned to structures denoted cc1 and cn1 with newly formed covalent C–C and C–N bonds between the two Py units as a result of complex intracluster ion–molecule reactions (including intermolecular proton transfer) triggered by VUV ionization.^43^ Similar ion–molecule reactions upon VUV ionization have previously been revealed in naphthalene-pyridine by mass spectroscopic measurements combined with quantum chemical calculations^44^ and in pyridine dimers by IRPD spectroscopy.^45^ Although, in the latter study, the authors provided spectroscopic evidence of the ion–molecule reaction in pyridine dimer cations, they did not specify whether the reaction products are generated from the ionization of neutral pyridine dimer or larger clusters such as trimers followed by postionization fragmentation.^45^ Therefore, the reaction mechanisms remain rather unclear in these VUV-produced cation clusters. For the example of VUV-generated Py_2_^+^,^43^ the suggested chemically bonded cc1 and cn1 isomers do not convincingly explain the broad and intense transition centered at 3020 cm^–1^ in the IRPD depletion spectrum, in particular, its blue tail ranging up to 3300 cm^–1^. A similar broad and intense band at 3150 cm^–1^ with blue tail ranges up to 3500 cm^–1^ was observed in the IR spectrum of VUV-generated (acrylonitrile-Py_2_)^+^ and explained by an NH···N H-bonded ν_NH_.^46^ In addition, according to the computed potential energy surface diagram,^43^ these ion–molecule reactions require at least 2.0 eV activation energy to be initiated from Py_2_^+^(a) via barriers in collision-free conditions. This energy is much higher than the dissociation energy of Py_2_^+^(a) of around 1.1 eV, and thus, it is rather unclear how these cc1/cn1 isomers can be stabilized under collision-free conditions. Because of the high energy available from VUV ionization of Py_*n*_, many possible reaction products and even direct fragmentation may be expected instead of forming stable cc1 and cn1 structures. Finally, the fragmentation pathways of the cc1 and cn1 structures to generate Py^+^ (or isobaric isomers) upon IRPD have remained unclear because they involve intermolecular proton transfer.

To shed further light on the spectroscopic assignments of the features observed in the IRPD spectra of Py_2_^+^ generated by VUV/EI, we compare, in the present study, IRPD spectra in the extended range of 2650–3550 cm^–1^ for Py_2_^+^ cations produced in the EI source with variable internal energy by tagging with N_2_ and Ar ligands and by generating Py_2_^+^ in the (i) high-pressure collision region or (ii) low-pressure collision-free region of the plasma expansion. In general, an upper limit for the internal energy is given by the binding energy of the most weakly bonded ligand in the cluster. The IRPD spectra of cold Py_2_^+^(Ar/N_2_)_*n*_ clusters minimize broadening arising from sequence hot bands and thus facilitate isomer identification. The IRPD spectra are analyzed by considering various intermolecular and chemically bonded isomers and their vibrational signatures obtained by dispersion-corrected density functional theory calculations at the B3LYP-D3/aug-cc-pVTZ level. This study extends previous work in two directions. First, we discuss the cold IRPD spectra of Py_2_^+^N_2_ and Py_2_^+^Ar_*n*_ to determine the population of the stacked Py_2_^+^(a) and Py_2_^+^(p) isomers. Second, we analyze the IRPD spectrum of bare Py_2_^+^ formed by EI and compare it to that of Py_2_^+^ generated by VUV ionization, discuss the effect of internal energy on the formation of Py_2_^+^, and offer alternative isomer assignments for the IRPD spectrum of Py_2_^+^ compared to the previous VUV work.^43^

## 2 Experimental and Computational Methods

IRPD spectra of mass-selected Py_2_^+^ and Py_2_^+^L_*n*_ clusters with L = Ar or N_2_ are recorded in the ν_CH/NH_ range (2650–3550 cm^–1^) in a quadrupole–octupole–quadrupole tandem mass spectrometer coupled to an EI source as described elsewhere.^47,48^ Briefly, the clusters are produced in a pulsed supersonic plasma expansion by EI of Py (Sigma-Aldrich, >98%, heated to 50 °C) seeded in Ar or N_2_ carrier gas (9–10 bar) close to the nozzle orifice (nozzle-in condition) and subsequent three-body clustering reactions. The filaments of the EI source are powered by an offset voltage of up to 200 V, which sets an upper limit for the kinetic energy of the electrons impacting the molecular beam to 200 eV. A possible production route for Py_2_^+^ and Py_2_^+^L_*n*_ clusters under this condition starts with EI of neutral Py (eq 1) followed by three-body collisions to form Py_2_^+^ (eq 2) and Py_2_^+^L_*n*_ (eq 3)^49,50^
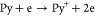
1
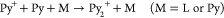
2
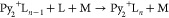
3

To generate Py_2_^+^ clusters with higher internal energy, the pulsed nozzle orifice is pulled out by approximately 1 cm from the electron impact (nozzle-out condition). Here, neutral Py_*n*_ clusters are thought to be generated first by three-body collisions in the supersonic expansion (eq 4), and then, these cold neutral clusters are ionized downstream by EI to generate the cation clusters (eq 5) in the collision-free region.
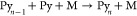
4
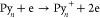
5

Thus, although these Py_*n*_^+^ cluster ions may cool somewhat by evaporative cooling, leading to smaller Py_*m*_^+^ clusters with *m* < *n*, they are directly extracted through the skimmer without further collisional cooling, resulting in potentially warmer clusters. This type of EI production mimics more closely the conditions using VUV ionization,^43^ apart from the higher available ionization excess energy in the EI method.

The ions generated in the central and coldest part of the plasma expansion are extracted through a skimmer into the first quadrupole, which is tuned to the mass of the parent cluster ion of interest. The mass-selected clusters are then deflected by 90° into the adjacent octupole ion guide and irradiated with an IR laser pulse (ν_IR_) emitted from an optical parametric oscillator pumped by a Q-switched nanosecond Nd:YAG laser, with pulse energies of 2–5 mJ, a repetition rate of 10 Hz, and a bandwidth of <2 cm^–1^. A wavemeter is used to calibrate the IR frequency to an accuracy of 1 cm^–1^. Resonant vibrational excitation followed by intracluster vibrational energy redistribution leads to the evaporation of the most weakly bonded ligands and generates daughter ions (eqs 6–7)
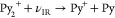
6
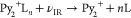
7

The resulting daughter ions are mass-selected by the second quadrupole and monitored by a Daly detector as a function of ν_IR_ to obtain the IRPD action spectrum of the parent clusters. The ion source is triggered at twice the laser frequency to separate the metastable decay background from the laser-induced dissociation signals (IRPD). All IRPD spectra are normalized for frequency-dependent variations in the IR photon flux measured by a pyroelectric detector. The peak widths of the vibrational transitions observed in the IRPD spectra are mainly caused by unresolved rotational structure, sequence hot bands involving inter- and intramolecular modes, and possibly contributions of several isomers. In addition to IRPD, low-energy collision-induced dissociation (CID) and metastable decay (MD) experiments are performed in the octupole ion guide to confirm the composition of the mass-selected parent clusters. For the CID spectra, the octupole is filled with N_2_ gas (10^–5^ mbar), resulting in collisions with 10 eV energy in the laboratory frame. This corresponds to center-of-mass collision energies of 3.2 and 1.9 eV for Py^+^ and Py_2_^+^, respectively.

Quantum chemical calculations are performed at the dispersion-corrected (unrestricted) B3LYP-D3/aug-cc-pVTZ level to obtain the geometries, energies, and harmonic vibrational spectra of Py_2_^+^ and their tagged clusters.^51^ This level reliably describes the electrostatic, induction, and dispersion forces, as demonstrated in our previous studies on clusters of Py^+^.^14,36,52^ Relative energies (*E*_e_) and dissociation energies of the ligand with the lowest binding energy (*D*_e_) are corrected for harmonic zero-point vibrational energies to derive *E*_0_ and *D*_0_. Corrections for basis set superposition error are not included because they are expected to be 1% (or less) for the large basis set employed.^53,54^ The Cartesian coordinates of all considered isomers and their corresponding energies are provided in the Supporting Information. Single-point energy calculations are carried out using CCSD and CBS-QB3 methods to confirm the reliability of the B3LYP-D3 energies. Harmonic vibrational frequencies are scaled by a factor of 0.9619 for ν_CH/NH_ obtained by comparison to experimental values of bare Py, ν_NH_ = 3531 cm^–1^, to compensate for effects of vibrational anharmonicity and errors in the harmonic force field.^36,52^ This procedure enables convenient comparison with measured frequencies and their assignments. Calculated vibrational spectra are convoluted with Lorentzian line profiles with fwhm = 4 cm^–1^ to facilitate the assignments of the IRPD spectra. Natural bond orbital (NBO) analysis is employed to evaluate the charge distribution in Py_2_^+^L_*n*_ clusters.

Anharmonic vibrational calculations are carried out at the lower B3LYP-D3/aug-cc-pVDZ level to reduce computational costs. The reliability of this level using a smaller basis set is evaluated by comparing the calculated harmonic and anharmonic spectra of Py to those calculated at the B3LYP-D3/aug-cc-pVTZ level. Then, both anharmonic calculations are compared to the experimental IR spectrum of Py.^55−57^ Harmonic vibrational frequencies calculated at the B3LYP-D3/aug-cc-pVDZ level are scaled by a factor of 0.9628 for ν_CH/NH_.

## 3 Results and Discussion

### 3.1 Overview of IRPD Spectra of Bare and Tagged Py_2_^+^ Clusters

**Figure 2:**
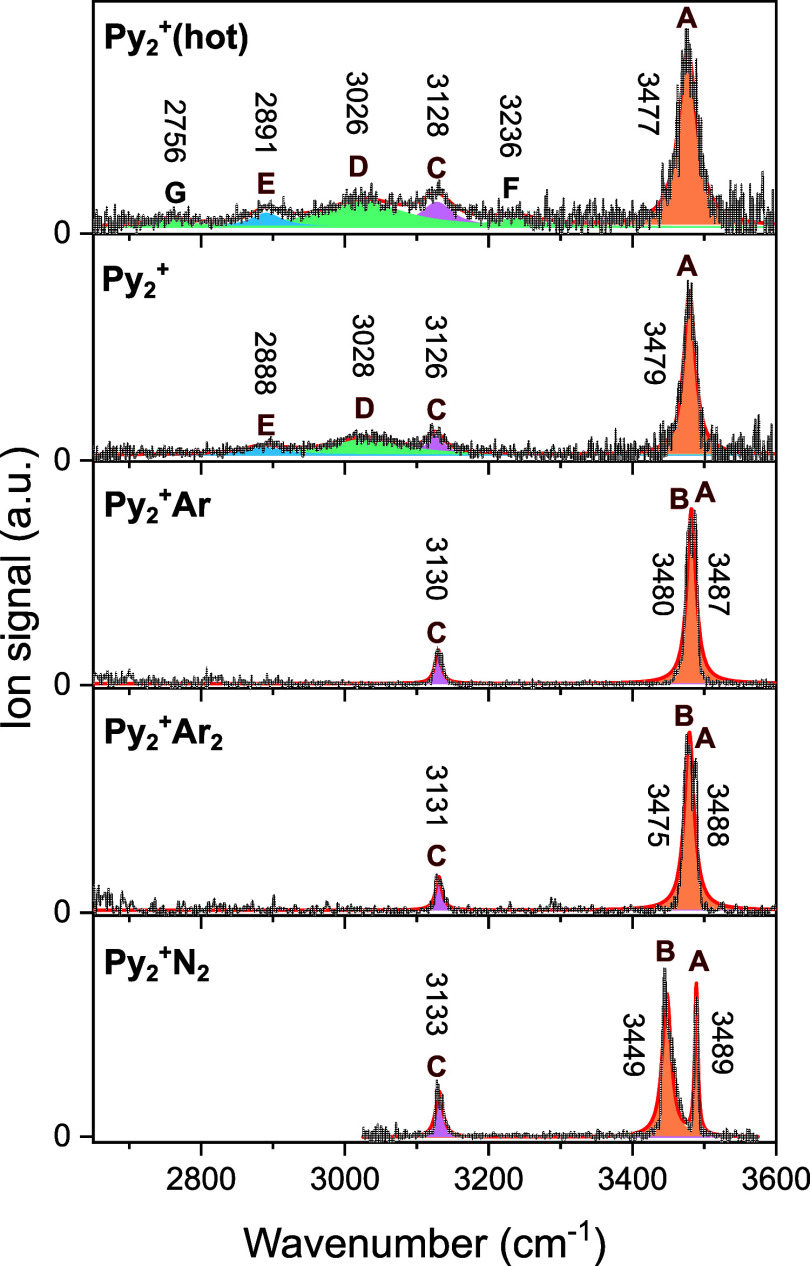
IRPD spectra of Py_2_^+^(hot), Py_2_^+^, Py_2_^+^Ar_*n*_ (*n* = 1–2), and Py_2_^+^N_2_ measured in the CH and NH stretch range and their deconvolution using Lorentzian line profiles. The respective peak positions and areas normalized to 1 are listed in Tables 1 and S1.

IRPD spectra of Py_2_^+^ and its cold Py_2_^+^Ar_1,2_ and Py_2_^+^N_2_ clusters are compared in Figure 2. The spectrum of bare Py_2_^+^ (nozzle-in) is monitored in the Py^+^ fragment channel, while the Py_2_^+^L_*n*_ cluster spectra are recorded in the Py_2_^+^ channel. The observed peak positions and widths are compiled in Table 1 along with the suggested vibrational and isomer assignments. The IRPD spectra of Py_2_^+^ and Py_2_^+^Ar_1,2_ are measured in the extended spectral range from 2650 to 3550 cm^–1^, while the Py_2_^+^N_2_ spectrum is obtained in the 3050–3550 cm^–1^ range. Thus, they cover the ν_NH_ (A, B) and ν_CH_ (C) bands of stacked Py_2_^+^. Overall, the IRPD spectra show good agreement with previous spectra.^14,43^ However, the intensity ratio of the ν_NH_ and ν_CH_ bands of our Py_2_^+^ spectrum is much higher than in the previously reported Py_2_^+^ spectrum^43^ because the ν_NH_ band is strongly saturated in the latter measurement. In comparison to the spectrum of bare Py_2_^+^, the spectra of the tagged clusters exhibit much narrower bands and distinct splittings of the ν_NH_ bands (3400–3500 cm^–1^), which facilitates isomer identification. On the other hand, the ν_CH_ bands (band C) are not greatly affected by tagging. Significantly, the Py_2_^+^ spectrum shows two additional broad peaks centered at 2888 and 3028 cm^–1^ (D, E). Because these transitions are not detected in the IRPD spectrum of Py_2_^+^Ar_*n*_ (and Py_2_^+^N_2_ for band D), this result may suggest that the population of structural isomers is different for Py_2_^+^ and Py_2_^+^L_*n*_ due to their different internal energy.

To elucidate the internal energy dependence on the Py_2_^+^ isomer population, we consider IRPD spectra of Py_2_^+^ recorded under different expansion conditions. The Py_2_^+^(hot) spectrum at higher internal energy measured for the nozzle-out condition is compared in Figure 2 to the spectrum of Py_2_^+^ with less internal energy recorded for the nozzle-in condition. The Py_2_^+^(hot) spectrum exhibits four major peaks (A, C–E) at 3477, 3128, 3026, and 2891 cm^–1^ with fwhm of 30, 53, 135, and 51 cm^–1^, respectively, which are also observed in the colder Py_2_^+^ spectrum, and two additional small and broad peaks F and G at 3236 and 2756 cm^–1^. Small shifts (up to ±3 cm^–1^) and substantially larger widths are observed for the major peaks in the Py_2_^+^(hot) spectrum, indicating that the clusters formed under the nozzle-out condition have indeed higher internal energy. The additional peak G could either be the result of hot bands or minor contributions of new isomers, and the signal F could simply be the tail of peak D. Overall, the Py_2_^+^(hot) spectrum suggests that the Py_2_^+^ isomer composition and their population are not substantially affected by the formation mechanism and the higher internal energy. To quantify the internal energy effects, the IRPD spectra of Py_2_^+^(hot) and Py_2_^+^ are deconvoluted using Lorentzian line profiles in Figure 2. The resulting peak positions and respective integrated areas are listed in Table S1, along with corresponding data for Py_2_^+^L_*n*_. The six peaks at 3477, 3128, 3026, 2891, 3236, and 2756 cm^–1^ observed for Py_2_^+^(hot) are deconvoluted with the respective areas of 0.45, 0.11, 0.29, 0.06, 0.06, and 0.04 (normalized to 1), while the spectrum of colder Py_2_^+^ is deconvoluted into four peaks at 3479, 3126, 3028, and 2888 cm^–1^ with areas of 0.51, 0.09, 0.32, and 0.08, respectively. The similar relative peak areas of both deconvoluted IRPD spectra for the four main bands support the interpretation that the isomer composition of bare Py_2_^+^ is indeed not greatly affected by its internal energy.

In the Py_2_^+^Ar spectrum, a clear splitting into two narrow ν_NH_ bands (A and B) is resolved due to their low internal energy arising from the low-binding energy of Ar. Significantly, the peaks D and E observed at 3028 and 2888 cm^–1^ for bare Py_2_^+^ are not present (or below the detection limit) for Py_2_^+^Ar_1,2_ (and also Py_2_^+^N_2_ for D). The deconvoluted Py_2_^+^Ar_*n*_ spectra have ν_NH_ and ν_CH_ peak areas of 0.88 and 0.12 (*n* = 1) and 0.89 and 0.11 (*n* = 2), while the corresponding peak areas of Py_2_^+^ are 0.51 and 0.09. Similar to Py_2_^+^Ar_*n*_, the IRPD spectrum of cold Py_2_^+^N_2_ exhibits two characteristic features in the ν_NH_ range (A and B). The ν_NH_ and ν_CH_ peak areas (0.84 and 0.16) are comparable to those of Py_2_^+^Ar_*n*_, despite its somewhat higher internal energy, indicating that the Py_2_^+^ isomer composition is rather similar in the relatively cold tagged Py_2_^+^L_*n*_ clusters. On the other hand, the bare Py_2_^+^ clusters appear to have more than one structural isomer because of the unique appearance of the two additional bands D and E and the different ν_NH_/ν_CH_ intensity ratio. Apparently, these extra Py_2_^+^ isomers do not form stable tagged Py_2_^+^L_*n*_ clusters, probably due to their high internal energy resulting from their formation process. In the following, we analyze the isomer contributions based on the IRPD spectra of the tagged and bare Py_2_^+^ clusters by focusing on the strongly IR active ν_NH_ bands because the unresolved ν_CH_ bands are not sensitive to the internal energy of the clusters and the ligand binding sites.

### 3.2 Py_2_^+^ Structures at Cold Conditions

#### 3.2.1 Py_2_^+^N_2_

**Figure 3:**
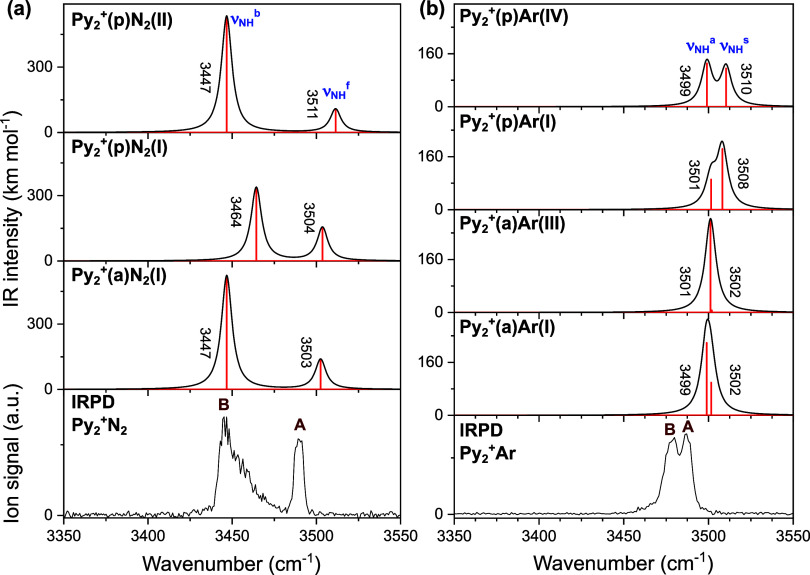
Comparison of IRPD spectra of (a) Py_2_^+^N_2_ and (b) Py_2_^+^Ar in the NH stretch range to linear IR absorption spectra computed for the most stable isomers of Py_2_^+^(a/p)N_2_ and Py_2_^+^(a/p)Ar at the B3LYP-D3/aug-cc-pVTZ level. The positions, widths, and vibrational and isomer assignments of the transitions observed are listed in Table 1.

We utilize the N_2_ tag to collect initial clues on the structural population of cold Py_2_^+^ isomers. N_2_ only slightly affects the Py_2_^+^ structure by the small intermolecular interaction based on its negative quadrupole moment and parallel polarizability. These cause weak electrostatic, inductive, and dispersive attraction between Py_2_^+^ and N_2_, while keeping the internal energy of the cluster substantially lower than in bare Py_2_^+^ due to a strongly reduced dissociation energy (*D*_0_ ∼ 15 versus ∼100 kJ mol^–1^).^36,50,58−61^ As a result, the IRPD spectrum of Py_2_^+^N_2_ exhibits two intense and well-resolved ν_NH_ peaks A and B. The optimized stable structures of Py_2_^+^N_2_ based on π-stacked Py_2_^+^ are shown in Figures 1 and S2. We consider the three main N_2_ binding motifs for attachment denoted NH, CH, and π. Because of the *C*_2*h*_ symmetry of Py_2_^+^(a), three minima are found for Py_2_^+^(a)N_2_, while five minima are obtained for Py_2_^+^(p)N_2_ due to the lower *C*_1_ symmetry of Py_2_^+^(p). In general, N_2_ binds with a linear rather than a T-shaped orientation toward the positive charge of Py_2_^+^ because of the angular anisotropy of the charge-quadrupole and charge-induced dipole interactions.^50,59^ The computed IR spectra of all considered isomers are compiled in Figures 3 and S3 (Tables 1 and S2).

In the Py_2_^+^(a)N_2_(I) global minimum, N_2_ binds to the NH group of one unit of Py_2_^+^ through a NH···N_2_ H-bond with *D*_0_ = 17.5 kJ mol^–1^. In the two Py_2_^+^(a)N_2_(II–III) local minima, N_2_ binds either to the C_α_H groups of both Py units in a bifurcated fashion or the π cloud with *D*_0_ = 12.9 and 11.8 kJ mol^–1^, respectively. Upon N_2_ complexation, the symmetry of Py_2_^+^(a) reduces from *C*_2*h*_ to *C*_s_. The N–H proton donor bond of the Py unit is elongated by 3 mÅ, resulting in a 53 cm^–1^ redshift in ν_NH_^a^ of Py_2_^+^(a). As a result, two distinct transitions are predicted at 3503 (ν_NH_^f^) and 3447 cm^–1^ (ν_NH_^b^) with a splitting of 56 cm^–1^ and enhanced IR intensities (138 and 523 km mol^–1^). Because in the less stable Py_2_^+^(a)N_2_(II–III) isomers, the N_2_ ligand binds to the C_α_H or π sites of the Py units, the N–H bonds are barely affected, and the calculated ν_NH_ bands are nearly identical to those of bare Py_2_^+^(a). Due to symmetry reduction, the ν_NH_^s^ bands are weakly activated (5 and 54 km mol^–1^). However, because ν_NH_^a,s^ of Py_2_^+^(a)N_2_(II–III) overlap with ν_NH_^f^ of Py_2_^+^(a)N_2_(I), it is difficult to evaluate their potential contribution to the measured IRPD spectrum and thus we consider only the Py_2_^+^(a)N_2_(I) global minimum further.

Py_2_^+^(p)N_2_ has five local minima, which include two H-bonded, one C_α_H bifurcated, and two π-bonded isomers. All of them are substantially higher in *E*_0_ (>7 kJ mol^–1^) than Py_2_^+^(a)N_2_(I). In the lowest energy structure, Py_2_^+^(p)N_2_(I), N_2_ binds with *D*_0_ = 17.9 kJ mol^–1^ to the NH group of the Py unit with the higher positive partial charge (+0.538*e*). However, *E*_0_ is only 0.6 kJ mol^–1^ larger for Py_2_^+^(p)N_2_(II) (*D*_0_ = 17.3 kJ mol^–1^), in which N_2_ binds to the other Py unit with +0.462*e*. Thus, both isomers should be considered. In the substantially less stable Py_2_^+^(p)N_2_(III) isomer, N_2_ binds in a bifurcated fashion to C_α_H groups of both Py units with *D*_0_ = 13.1 kJ mol^–1^. This isomer is higher in *E*_0_ than Py_2_^+^(p)N_2_(I) by 4.8 kJ mol^–1^. Similar to Py_2_^+^(a)N_2_(II), N_2_ binding has a negligible effect on the N–H bond lengths, and thus, the computed ν_NH_ frequencies are nearly identical to those of Py_2_^+^(p), with a splitting of 11 cm^–1^. In Py_2_^+^(p)N_2_(IV–V), N_2_ attaches to the π-electron system of the bottom or top Py unit with the same *D*_0_ of 12.0 kJ mol^–1^. Again, similar to Py_2_^+^(p)N_2_(III), their predicted IR spectra are almost identical to that of Py_2_^+^(p). Therefore, we exclude Py_2_^+^(p)N_2_(III–V) from further assignment because their predicted ν_NH_ splitting is too small compared to the experimental one. In Py_2_^+^(p)N_2_(I), N_2_ binds almost linearly to the NH group of the bottom Py. However, its NH···N_2_ H-bond is longer than that of Py_2_^+^(a)N_2_(I) (2.228 vs 2.202 Å), although the partial charge on the Py unit is higher (+0.538 vs +0.500*e*), because N_2_ is tilted away from the top Py unit. Nevertheless, *D*_0_ is higher by 0.4 kJ mol^–1^ due to the slightly higher positive partial charge. The N–H proton donor bond elongates by 2 mÅ, resulting in redshifts of 6 and 35 cm^–1^ for ν_NH_^f^ and ν_NH_^b^ predicted at 3504 and 3464 cm^–1^, respectively, with a splitting of 40 cm^–1^. When N_2_ attaches to the top Py unit (Py_2_^+^(p)N_2_(II)), N_2_ tilts away from the bottom Py unit. Because the orientation between N_2_ and Py is more favorable than in Py_2_^+^(p)N_2_(I), the NH···N_2_ H-bond is shorter (2.210 Å) in Py_2_^+^(p)N_2_(II). However, *D*_0_ is lower by 0.6 kJ mol^–1^ because of a slightly smaller positive partial charge on the top Py unit. N_2_ binding causes an N–H bond elongation of 3 mÅ, leading to a redshift of 52 cm^–1^ in ν_NH_^b^. As a result, ν_NH_^b^ and ν_NH_^f^ are predicted at 3447 and 3511 cm^–1^, with a splitting of 64 cm^–1^. This substantial difference in peak splitting between these two isomers is again caused by the uneven charge distribution on the Py units.

The IRPD spectrum of Py_2_^+^N_2_ is compared in Figure 3 to the computed IR spectra of the most stable H-bonded isomers. The IRPD spectrum exhibits two distinct single peaks A and B at 3489 and 3449 cm^–1^ in the ν_NH_ range with fwhm of 5 and 17 cm^–1^, respectively, which are readily assigned to ν_NH_^f^ and ν_NH_^b^. Peak A is narrow and symmetric while peak B is broad, blue-shaded, and intense. Peak B has the characteristic contour of an H-bonded XH proton donor stretch fundamental, with a sharp P-branch head and a long blue-shaded tail. Such a band shape arises from an increase in rotational constants upon vibrational excitation and the excitation of sequence hot bands involving intermolecular modes.^48,59,62^ This band indicates that an H-bonded Py_2_^+^N_2_ isomer is present. Because of the detection of only a single P-branch head of band B and the sharp and symmetric appearance of peak A, we conclude that the spectrum is dominated by a single isomer, while others are below the detection limit (<15%), considering the achieved signal-to-noise ratio, the computed IR oscillator strengths, and the similar N_2_ binding energies. The calculations predict three low-energy H-bonded isomers, namely Py_2_^+^(a)N_2_(I) and Py_2_^+^(p)N_2_(I–II). The ratio of the integrated peak areas of B and A determined as 3.0 is closer to the corresponding IR cross-section ratio of Py_2_^+^(a)N_2_(I) (3.8) than those of Py_2_^+^(p)N_2_(I–II) (2.2 and 5.0). In addition, the measured splitting of 40 cm^–1^ is close to that predicted for the global minimum (56 cm^–1^). Thus, considering the predicted relative energies, peak splittings, and relative intensities, peaks A and B are assigned to ν_NH_^f^ and ν_NH_^b^ of Py_2_^+^(a)N_2_(I). As the *D*_0_ values of H-bonded Py_2_^+^(a/p)N_2_ are similar (∼17 kJ mol^–1^), their N_2_ tagging efficiencies are similar, too. Thus, the detection of only one Py_2_^+^N_2_ isomer is taken as strong evidence that the cold Py_2_^+^ population is largely dominated by the most stable Py_2_^+^(a) structure, while Py_2_^+^(p) is below the detection limit.

**Figure 4:**
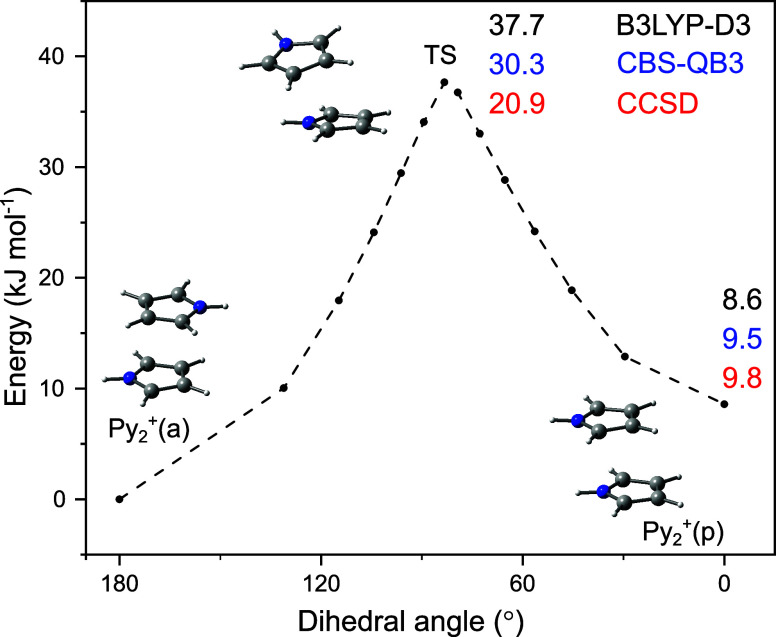
Potential energy surface (uncorrected for zero-point energy) for the internal rotation of Py_2_^+^(a) into Py_2_^+^(p) as a function of the NH···HN dihedral angle using the nudged elastic band method (B3LYP-D3/aug-cc-pVTZ). The corresponding single-point energies obtained at the CCSD and CBS-QB3 levels are also included for the stationary points, respectively.

To rationalize the Py_2_^+^(a/p) isomer ratio deduced from the IRPD spectrum of Py_2_^+^N_2_, we consider the potential energy surface for internal rotation converting Py_2_^+^(a) into Py_2_^+^(p) using the nudged elastic band method implemented in the ORCA software package^63,64^ at the B3LYP-D3/aug-cc-pVTZ level (Figure 4). The relative energies of Py_2_^+^(a) and Py_2_^+^(p) are *E*_e_ = 0 and 8.6 kJ mol^–1^ (*E*_0_ = 0 and 7.5 kJ mol^–1^) and the rotational barriers are *V*_b_(a → p)=37.7 and *V*_b_(p → a) = 29.2 kJ mol^–1^ for the forward and backward reaction (uncorrected for zero-point energies), respectively. The corresponding single-point energies at the CBS-QB3 and CCSD levels are rather similar, Δ*E*_e_/Δ*E*_0_(CBS-QB3) = 9.5/8.7 and Δ*E*_e_(CCSD) = 9.8 kJ mol^–1^ and *V*_b_(a → p) = 30.3 (CBS-QB3) and 20.9 (CCSD). The high rotational *V*_b_ barrier suggests that once less stable Py_2_^+^(p)N_2_ isomers are formed during the expansion, a substantial fraction of them may be kinetically trapped in their potential well.^65^ On the other hand, the abundance of Py_2_^+^(p) may be estimated as only 17% at 298 K, using the Boltzmann distribution assuming Δ*E*_0_(B3LYP-D3) = 7.5 kJ mol^–1^, revealing that our experimental result is consistent with the calculation.

#### 3.2.2 Py_2_^+^Ar

We utilize Ar tagging to further reduce the internal energy of Py_2_^+^ and the impact of the tag on the Py_2_^+^ structure. The most stable structures computed for Py_2_^+^Ar based on Py_2_^+^(a/p) are displayed in Figures 1 and S4. The calculation yields three favorable nonequivalent binding sites for Ar, namely NH, CH, and π for Py_2_^+^(a/p), with three minima for Py_2_^+^(a)Ar and five minima for Py_2_^+^(p)Ar due to its lower *C*_1_ symmetry. Their computed IR spectra are compared in Figures 3 and S5 (Tables 1 and S2). In general, Ar binds weaker to Py_2_^+^ than N_2_ (e.g., *D*_0_ = 7.6 vs 17.5 kJ mol^–1^ for Py_2_^+^(a)Ar(I) and Py_2_^+^(a)N_2_(I), respectively) due to the additional induction and electrostatic charge-quadrupole interactions for the N_2_ clusters. Therefore, Ar tagging yields potentially colder clusters than N_2_ tagging, with less impact on the intramolecular bond lengths (e.g., Δ*r*_NH_ = 0.2 vs 3 mÅ for the N–H donor bond). Because of the weaker interaction, the difference between H-bonded and π-bonded isomers is smaller for Ar than for N_2_, 2.8 vs 5.6 kJ mol^–1^. The *E*_0_ energies of Py_2_^+^(a)Ar(I–III) lie all within 3.0 kJ mol^–1^, which makes it difficult to rule out the contributions of any of them based on energetic arguments. Ar attachment reduces the symmetry of Py_2_^+^(a) from *C*_2*h*_ to *C*_*s*_ and activates ν_NH_^s^ of Py_2_^+^(a), which is IR forbidden in bare Py_2_^+^(a). It is calculated at 3502 cm^–1^, i.e., 3 cm^–1^ higher than ν_NH_^a^ (3499 cm^–1^) of Py_2_^+^(a)Ar(I) and 1 cm^–1^ higher than ν_NH_^a^ (3501 cm^–1^) of Py_2_^+^(a)Ar(II–III). The Py_2_^+^(p)Ar(I–V) structures have *E*_0_ energies higher by 6.4–10.3 kJ mol^–1^ compared to Py_2_^+^(a)Ar(I). Their ν_NH_^s^ and ν_NH_^a^ are barely changed compared to that of Py_2_^+^(p) due to weak interaction with Ar, with a splitting of 7 cm^–1^ (3508 and 3501 cm^–1^) for Py_2_^+^(p)Ar(I) and 11 cm^–1^ (3510 and 3499 cm^–1^) for Py_2_^+^(p)Ar(II–V).

The IRPD spectrum of Py_2_^+^Ar exhibits two ν_NH_ bands A and B at 3487 and 3480 cm^–1^, with fwhm of 8 and 13 cm^–1^ (Figure 3), respectively. These distinctive symmetric peaks are split by only 7 cm^–1^ and have similar IR intensities. However, they cannot be easily assigned to one of the proposed structures despite the cold spectrum of Py_2_^+^ obtained by Ar tagging. Nevertheless, because the Py_2_^+^N_2_ spectrum clearly reveals the dominant abundance of Py_2_^+^(a)N_2_, it is likely that the Py_2_^+^Ar population is also dominated by the most stable Py_2_^+^(a)Ar isomer. Therefore, peaks A and B are currently assigned to ν_NH_^f^ and ν_NH_^b^ of Py_2_^+^(a)Ar(I–III), respectively.

#### 3.2.3 Py_2_^+^Ar_*n*_ (*n* = 2–5)

To further study the internal energy effect on Py_2_^+^ formation and to extract experimental information about the Ar binding energy, IRPD spectra of Py_2_^+^Ar_*n*_ (*n* = 2–5) are recorded from 2650 to 3600 cm^–1^ (*n* = 2, Figure 2) and 3050 to 3550 cm^–1^ (*n* = 3–5, Figure S6 and Table S3). All IRPD spectra are obtained in the dominant Py_2_^+^ channel. The IRPD spectrum of Py_2_^+^Ar_2_ has only four main transitions at 3487, 3479, and 3475 cm^–1^ (ν_NH_) and 3131 cm^–1^ (ν_CH_), and no transitions below 3100 cm^–1^, which again indicates that only clusters with stacked Py_2_^+^ form at low internal energy conditions. The ν_NH_ and ν_CH_ bands become sharper with increasing *n*, indicating the decreasing temperature (internal energy) of the larger clusters. The ν_NH_ bands of Py_2_^+^ shift to lower frequency with increasing *n*, indicating the progressive solvation of the two NH groups of Py_2_^+^. Interestingly, the IR spectra of Py_2_^+^Ar_*n*_ with *n* = 1–4 have the same transition at 3489 cm^–1^ (ν_NH_^f^) regardless of *n*, assigned to free NH group(s). Significantly, its intensity decreases with *n*, indicating again the progressive solvation of the two NH groups by Ar ligands. The constant appearance of ν_NH_^f^ at the same frequency for *n* = 1–4 suggests that all of these Py_2_^+^Ar_*n*_ clusters have the same Py_2_^+^(a) core. Unfortunately, the nearly identical ν_NH_ and *E*_0_ values of the many calculated isomers for Py_2_^+^Ar_*n*_ (*n* = 2–5, Figures S7–S11) prevent a detailed isomer assignment. IRPD of Py_2_^+^Ar_5_ at 3130 cm^–1^ (37.4 kJ mol^–1^) is sufficient to photodissociate into Py_2_^+^ with the loss of all five Ar atoms. This result is consistent with the total binding energy of the most stable Py_2_^+^(a)Ar_5_ isomer, calculated as *D*_0_ = 35.7 kJ mol^–1^, and provides further evidence for the reliability of the B3LYP-D3 approach.

### 3.3 Py_2_^+^ Structure at Warm Conditions

The IRPD spectrum of bare Py_2_^+^ is monitored in the Py^+^ channel (Figure 2). The absorbed photon energy (ν_IR_) is substantially lower than *D*_0_ estimated as *D*_0_ = 107.4 and 99.9 kJ mol^–1^ (8978 and 8351 cm^–1^) for Py_2_^+^(a) and Py_2_^+^(p), respectively. Therefore, under our single-photon absorption conditions, only hot (stacked) clusters with an internal energy of at least 6000 cm^–1^ can be detected. The IR spectrum exhibits four transitions (A, C–E) at 3479, 3126, 3028, and 2888 cm^–1^ with fwhm values of 19, 29, 116, and 68 cm^–1^, respectively. In contrast to the tagged clusters, the ν_NH_ and ν_CH_ bands of bare Py_2_^+^ are broader and do not exhibit any splitting due to their higher internal energy, which prevents any isomer assignment to a/p. Moreover, the two new and broad transitions D and E below 3100 cm^–1^ are not predicted in the harmonic IR spectra of Py_2_^+^(a/p). Thus, the contribution of further isomers of Py_2_^+^ with different composition has to be considered. In the previous report,^43^ these additional peaks have been assigned to ν_CH_ bands of the chemically bonded cc1 and cn1 structures. Thus, we calculate herein their optimized geometries and IR spectra at our favored B3LYP-D3/aug-cc-pVTZ level and compare them to their computed spectrum^43^ and our IRPD spectrum measured for Py_2_^+^ (Figure S12 and Table S4). Our calculations predict similar transitions with comparable oscillator strengths for cc1 and cn1 as in ref (43), although they are slightly blueshifted by 20–30 cm^–1^. In the considered spectral range, cc1 has five main transitions at 3505, 3125, 3105, 2936, and 2907 cm^–1^, whereas cn1 has four bands at 3511, 3135, 3109, and 2912 cm^–1^. While their ν_CH_ modes near 2900 cm^–1^ can be assigned to the peak observed at 2888 cm^–1^, none of their scaled harmonic frequencies can account for the broad peak at 3028 cm^–1^, in particular its blue tail extending up to 3300 cm^–1^. Therefore, harmonic calculations of Py_2_^+^(a/p) and cc1/cn1 alone cannot fully explain all major features observed in the IRPD spectrum of Py_2_^+^, in contrast to the claim in ref (43).

To this end, anharmonic calculations of Py_2_^+^(a/p) and cc1/cn1 are conducted at the reduced B3LYP-D3/aug-cc-pVDZ level to evaluate the possible contribution of overtones and combination bands. By comparing harmonic and anharmonic IR spectra of neutral Py using the aug-cc-pVTZ and aug-cc-pVDZ basis sets, we confirm the reliability of reducing the basis set. Indeed, the anharmonic spectra of Py are very similar at both levels in the 3100–2650 cm^–1^ range. Moreover, they compare favorably with the measured IR spectrum of Py (Figure S13).^55^ The anharmonic IR spectra obtained for Py_2_^+^(a/p) between 2650 and 3100 cm^–1^ predict several combination bands, which can potentially contribute to the transition at 2888 cm^–1^. However, they still cannot explain the broad transition observed at 3028 cm^–1^ with its long blue tail (Figure S12). Figure S12 also includes the anharmonic IR spectra of cc1 and cn1. Their predicted ν_NH_ and ν_CH_ bands are consistent with both the harmonic calculations and the IRPD spectrum (bands A and C). The anharmonic cc1 spectrum has several combination bands and overtones, which may contribute to the 2888 cm^–1^ band (E) but can again not explain the broad 3028 cm^–1^ band (D). Similarly, cn1 has multiple combination bands and overtones near 2888 cm^–1^, while only a few transitions with very low oscillator strengths (<1% of the major transition) are predicted around 3028 cm^–1^. However, because of their rather low relative intensities, these transitions are unlikely to account for the intense and broad 3028 cm^–1^ peak. Thus, further isomers need to be considered to assign the major transition D.

**Figure 5:**
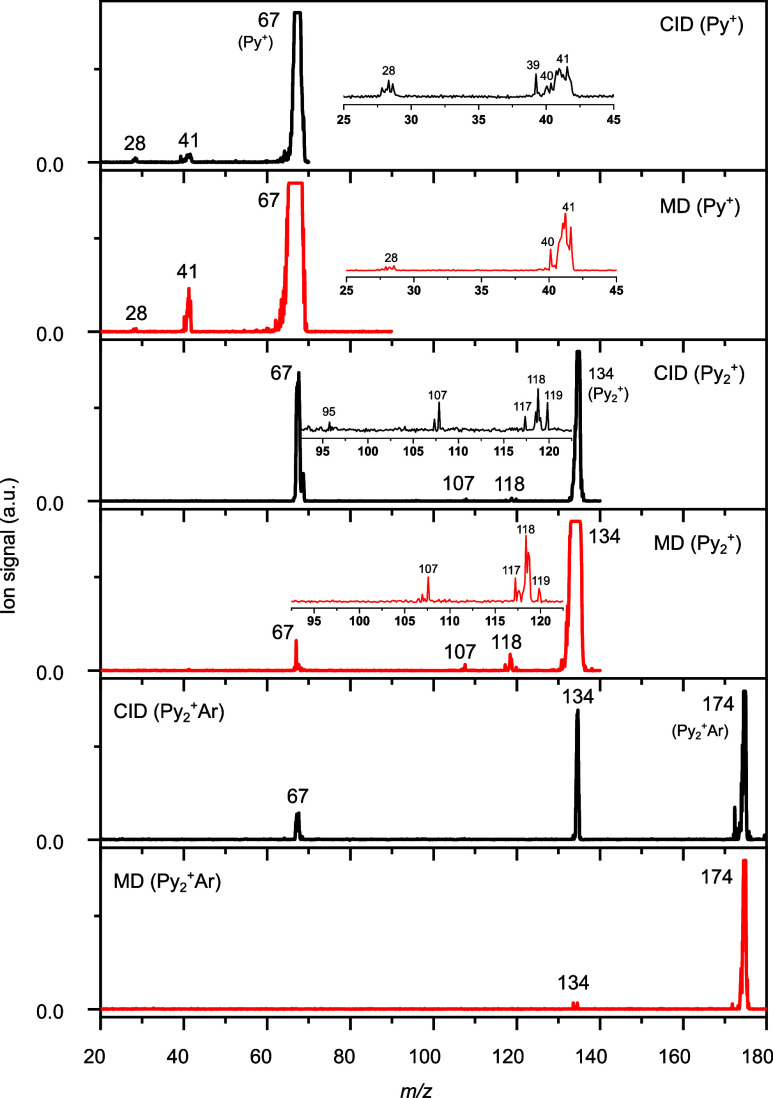
Collision-induced dissociation (CID) and metastable decay (MD) spectra of Py^+^, Py_2_^+^, and Py_2_^+^Ar.

The CID and MD spectra in Figure 5 provide interesting insight into possible structures of the observed Py_2_^+^ isomers. The CID spectrum of Py_2_^+^Ar (*m*/*z* 174) exhibits two fragment peaks at *m*/*z* 134 and 67, resulting from sequential loss of Ar and Py. The absence of additional peaks confirms the sole presence of Py_2_^+^Ar. The MD spectrum of Py_2_^+^Ar exhibits only loss of Ar due to its low-binding energy and illustrates the relatively high stability of Py_2_^+^. Interestingly, the CID spectrum of bare Py_2_^+^ (*m*/*z* 134) shows additional unexpected fragment peaks at *m*/*z* 95, 107, and 117–119 besides the expected Py^+^ daughter at *m*/*z* 67. This result reveals the presence of other dissociation channels of *m*/*z* 134 ions, with neutral mass losses of 39, 27, and 17–15 u. Because the CID experiment of Py_2_^+^Ar reveals that the intermediate π-stacked Py_2_^+^ fragment dissociates further into only Py^+^, the additional dissociation channels observed for CID of *m*/*z* 134 are taken as strong indication for the presence of other Py_2_^+^ isomers. Previous fragmentation studies on various cyclic and noncyclic Py^+^ monomer isomers (*m*/*z* 67) such as pyrrole, cyclopropyl cyanide, crotonitrile, allylcyanide, and methacrylonitrile showed fragmentation into *m*/*z* 28, 39, 40, 41, 51, and 52, corresponding to neutral losses of 39, 28, 27, 26, 16, and 15 u, respectively.^66−68^ Their relative intensities depend strongly on the parent structures and, thus, may be used for isomer identification.^66^ Our CID spectrum of *m*/*z* 67 shows three major fragments at *m*/*z* 28 (CH_2_N^+^), 39 (C_3_H_3_^+^), and 41 (C_3_H_4_^+^), and a less intense one at *m*/*z* 40 (C_2_H_3_N^+^), which are indeed all EI fragments of heterocyclic Py.^55^ However, this observation does not exclude the existence of other Py^+^ isomers because these are postulated to isomerize to a common structure prior to fragmentation, presumably the five-membered Py^+^ ring, once the energy exceeds a certain threshold.^66,69^ To this end, the MD spectrum of *m*/*z* 67 recorded under collision-free conditions in the octupole results from ions with reduced internal energy compared to CID and are, thus, less prone to isomerization of the Py^+^ isomers prior to fragmentation. Indeed, the MD spectrum of *m*/*z* 67 differs from the CID spectrum, with peaks at *m*/*z* 28, 40, and 41. Moreover, the intensity ratios of *m*/*z* 28/41 and 39/41 decrease substantially while the peak ratio of *m*/*z* 40/41 increases. Since heterocyclic Py^+^ is relatively stable under collision-free conditions, these differences in peak intensity ratios further indicate the existence of other Py^+^ isomers. As previously reported, an increase of fragment *m*/*z* 40 and a decrease of *m*/*z* 28 are expected from open-ring isomers (denoted here open-cycle, OC), such as crotonitrile and allylcyanide.^66^ The mass losses observed in our CID spectrum of *m*/*z* 134 ions are consistent with some of the fragmentation channels of Py^+^ isomers, suggesting that the unexpected fragment ions result from a nonheterocyclic Py isomer in Py_2_^+^. The small CID peak at *m*/*z* 95 (28^+^ + 67) is a potential fragment of heterocyclic Py^+^. On the other hand, *m*/*z* 107 (40^+^ + 67) is a potential fragment ion of open-ring isomers (Py(OC)···Py)^+^, similar to *m*/*z* 117 (50^+^ + 67), 118 (51^+^ + 67), and 119 (52^+^ + 67).^66^ In addition, MD of Py_2_^+^ isomers (*m*/*z* 134) produces the main unexpected fragments *m*/*z* 107 and 117–119 of (Py(OC)-Py)^+^. Previously, Py(OC)^+^ structures were found to undergo fragmentation faster than the five-membered Py^+^ ring,^69^ which supports our results. Because the CID and MD spectra of Py_2_^+^ suggest other isomer contributions with one intact Py unit (as expected from eq 2), we focus our search of potential Py_2_^+^ isomers in the following on candidate structures produced by combining a Py^+^ isomer with neutral heterocyclic Py.

The main MD channels of Py^+^ in Py_2_^+^ are *m*/*z* 40 and 50–52. The product analyses upon UV and EI ionization of Py along with thermochemical considerations agree with the assignment of *m*/*z* 40 and 52 to C_3_H_4_^+^ (HCN loss) and C_3_H_2_N^+^ (CH_3_ loss), respectively, while the identity of *m*/*z* 50 and 51 has not been clarified so far.^68,70−72^ The fragment ions *m*/*z* 40, 51, and 52 are mainly attributed to open-ring isomers derived from crotonitrile and allylcyanide.^66^ The *m*/*z* 40 ion was suggested to arise from hydrogen migration (H-migration) prior to ring opening.^68^ Therefore, based on our CID and MD analysis, it is reasonable to consider H-migrated closed-ring (Py(CC)^+^) and open-ring (Py(OC)^+^) isomers derived from crotonitrile and allylcyanide (Figure S14). The maximum EI energy of 200 eV can certainly provide enough energy to induce such ring-opening reactions of Py^+^ in the employed plasma ion source.

**Figure 6:**
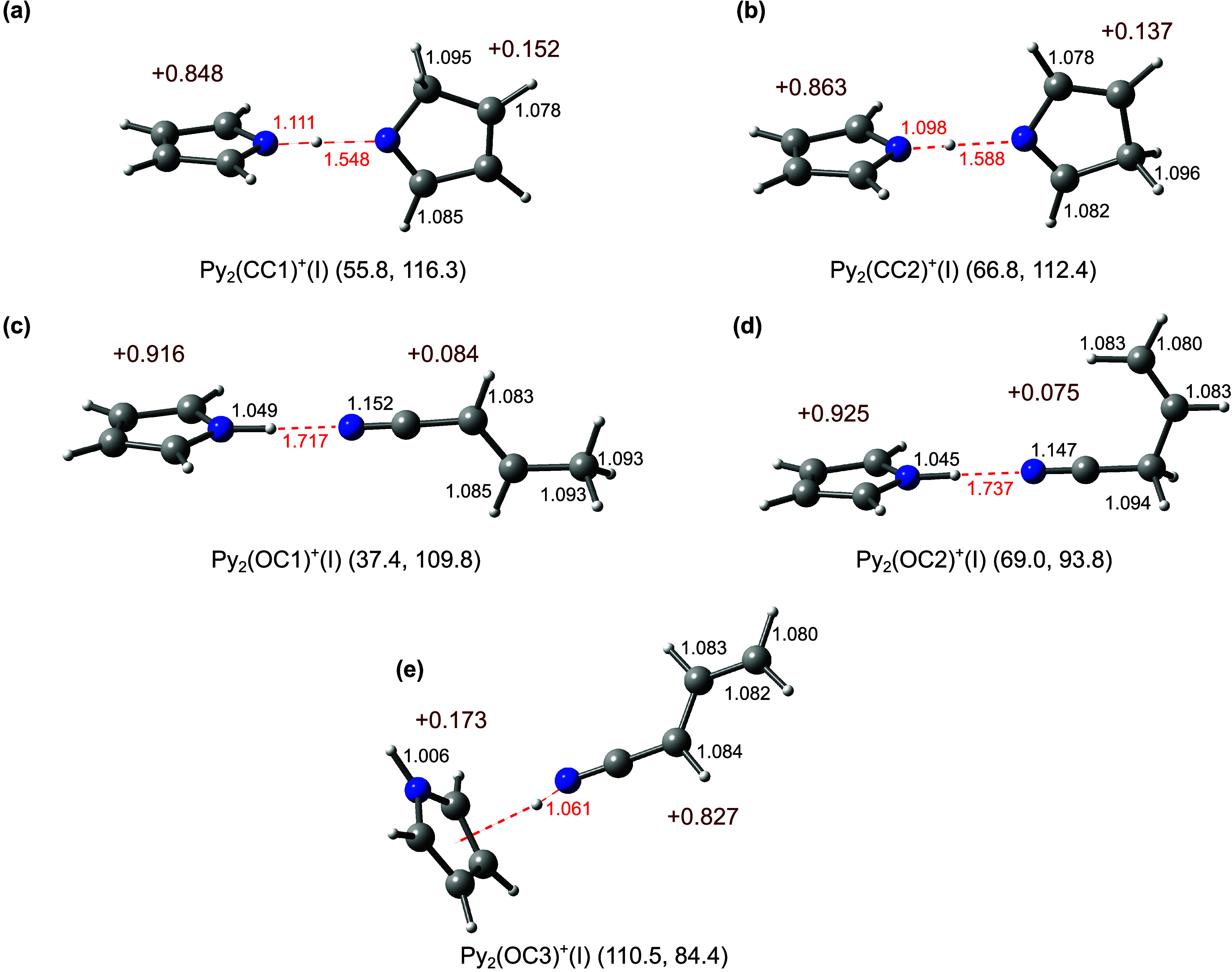
Optimized structures of low-energy Py_2_(CC)^+^ and Py_2_(OC)^+^ isomers obtained at the B3LYP-D3/aug-cc-pVTZ level. Selected intra- and intermolecular lengths (in Å) are indicated in black and red colors, respectively. Values in dark red indicate the molecular charges (in units of *e*). The energies in parentheses correspond to relative energies and dissociation energies (*E*_0_ and *D*_0_ in kJ mol^–1^). Further higher-energy isomers of Py_2_(CC)^+^ and Py_2_(OC)^+^ are shown in Figures S15 and S17.

**Figure 7:**
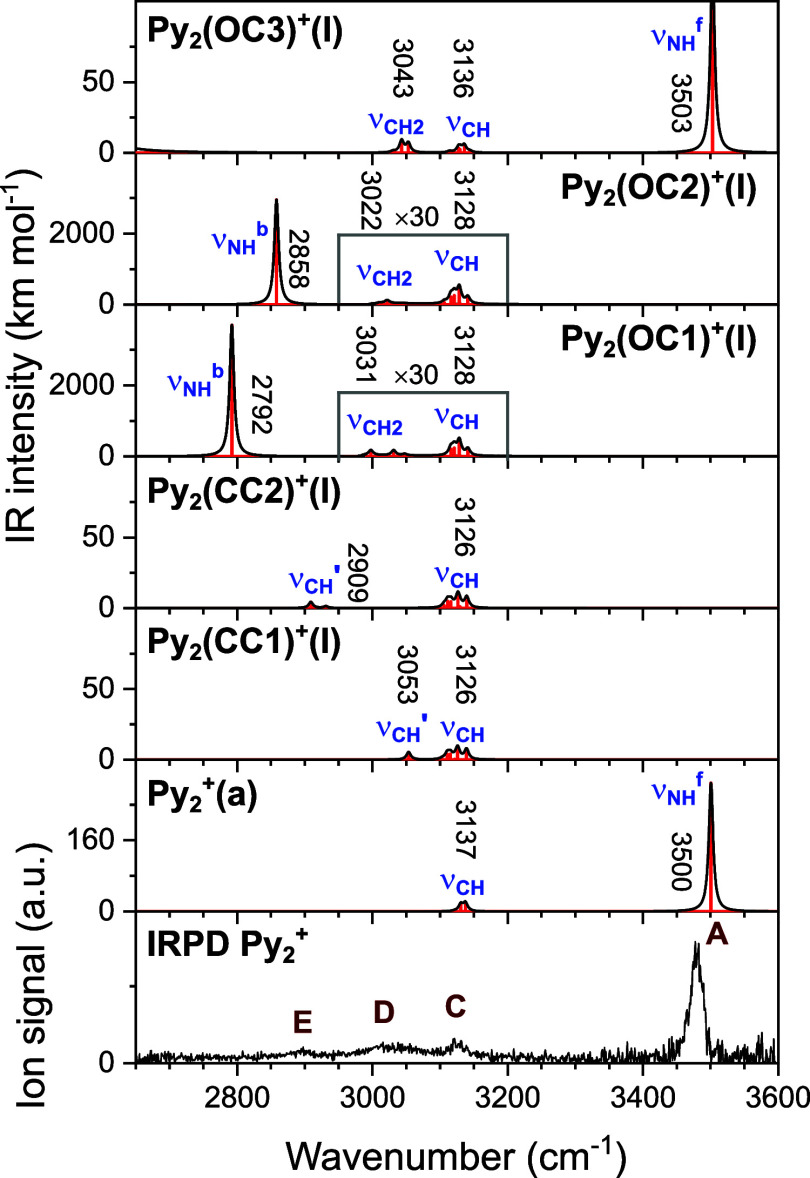
Comparison of IRPD spectra of Py_2_^+^ in the CH and NH stretch ranges to linear IR absorption spectra of the low-energy isomers of Py_2_^+^(a), Py_2_(CC)^+^, and Py_2_(OC)^+^ computed at the B3LYP-D3/aug-cc-pVTZ level. The linear IR absorption spectra of Py_2_(OC1)^+^ and Py_2_(OC2)^+^ are vertically expanded by a factor of 30 in the range of 2950–3200 cm^–1^. The positions, widths, and vibrational and isomer assignments of the transitions observed are listed in Table 1.

Figure S14 shows the two Py(CC)^+^ isomers produced by H-migration from N to C_α_ and C_β_, denoted Py(CC1)^+^ and Py(CC2)^+^, respectively, which are substantially less stable than the canonical Py^+^ ion (*E*_0_ = 173.5 and 155.7 kJ mol^–1^). In Py(CC1)^+^, the hybridization of C_α_ changes from sp^2^ to sp^3^. Computed intermolecular minima of Py_2_(CC1)^+^ dimers formed from Py(CC1)^+^ and neutral Py are shown in Figures 6 and S15. The Py_2_(CC1)^+^(I) global minimum is stabilized by a strong ionic NH···N H-bond between the N–H group of Py^+^ and N of Py(CC1) with *D*_0_ = 116.3 kJ mol^–1^. Interestingly, the positive charge in this dimer is now mostly localized on Py^+^ (+0.848*e*), indicating an exothermic charge transfer process when forming the Py_2_(CC1)^+^(I) dimer from Py and Py(CC1)^+^. This result is consistent with the computed IE values of 780.6 and 889.3 kJ mol^–1^ (8.09 and 9.22 eV) for Py and Py(CC1) (Table S5). Despite of its high binding energy *D*_0_ due to its strong H-bond, its relative energy lies still higher than that of the Py_2_^+^(a) global minimum by *E*_0_ = 55.8 kJ mol^–1^. Its computed IR spectrum predicts two ν_CH_ bands at 3126 and 3053 cm^–1^ for the most intense transitions of the aromatic (ν_CH_) and aliphatic (ν_CH_′) modes, respectively (Figure 7 and Table 1). Its ν_NH_^b^ band is strongly red-shifted down to 1878 cm^–1^ (i.e., far outside the scanned range) because the N–H donor bond elongates to 1.111 Å due to the strong N–H···N H-bond. Corresponding minima of Py_2_(CC2)^+^ resulting from Py(CC2)^+^ are also included in Figures 6 and S15. The lowest energy isomer, Py_2_(CC2)^+^(I), exhibits again a strong ionic N–H···N H-bond (*D*_0_ = 112.4 kJ mol^–1^, *E*_0_ = 66.8 kJ mol^–1^), and its IR spectrum is characterized by two ν_CH_ bands at 3126 (ν_CH_) and 2909 cm^–1^ (ν_CH_′) and ν_NH_^b^ at 2055 cm^–1^ (Figure 7 and Table 1). Again, the positive charge in Py_2_(CC2)^+^(I) is mostly localized on the Py unit (+0.863*e*), consistent with the notation Py^+^···Py(CC2) and the corresponding computed IE values (780.6 and 864.4 kJ mol^–1^). Further less stable Py_2_(CC1/2)^+^ minima and their IR spectra are shown in Figures S15 and S16, respectively, but are not considered further because they are much higher in energy.

Based on our CID and MD results, we propose the three open-ring ions Py(OC1–3)^+^ derived from crotonitrile, allylcyanide, and an isomer of allylcyanide arising from H-migration,^73^ with relative energies of *E*_0_ = 203.4, 247.0, and 87.5 kJ mol^–1^, respectively (Figure S14). Optimized structures of corresponding Py_2_(OC)^+^ dimers are displayed in Figures 6 and S17. The Py_2_(OC1)^+^(I) global minimum is stabilized by an ionic NH···N H-bond between of Py^+^ (0.916*e*) and Py(OC1), again indicating charge transfer from Py(OC1)^+^ to Py upon formation of this dimer (ΔIE = 163.7 kJ mol^–1^ for Py and Py(OC1)). Py_2_(OC1)^+^ is higher in energy than Py_2_^+^(a) by only 37.4 kJ mol^–1^, indicating that the strong interaction between Py^+^ and Py(OC1) and exothermic charge transfer mostly compensates for the high *E*_0_ of Py(OC1)^+^ relative to Py^+^ (203.4 kJ mol^–1^). Its computed IR spectrum (Figure 7) is characterized by three main transitions at 3128 (ν_CH_), 3031 (ν_CH2_), and 2792 cm^–1^ (ν_NH_^b^). Similar to Py_2_(OC1)^+^(I), Py_2_(OC2)^+^(I) is also stabilized through a strong ionic H-bond (*D*_0_ = 93.8 and *E*_0_ = 69.0 kJ mol^–1^). The lower *D*_0_ and higher *E*_0_ values are a result of the lower stability of Py(OC2)^+^ compared to Py(OC1)^+^, Δ*E*_0_ = 43.6 kJ mol^–1^ (Figure S14).^69^ Its predicted IR spectrum has three major transitions at 3128 (ν_CH_), 3022 (ν_CH2_), and 2858 cm^–1^ (ν_NH_^b^). Py_2_(OC3)^+^(I) is stabilized by a strong NH···π H-bond (cation–π interaction) between the NH group of Py(OC3)^+^ and the aromatic π system of Py with *D*_0_ = 84.4 kJ mol^–1^ and *E*_0_ = 110.5 kJ mol^–1^. The calculated IR spectrum predicts four transitions including ν_NH_^f^ (3503 cm^–1^), ν_NH_^b^ (2570 cm^–1^, outside the scanned range), ν_CH_ (3136 cm^–1^), and ν_CH2_ (3043 cm^–1^). In contrast to Py_2_(OC1/2)^+^(I), this dimer does not exhibit charge transfer because the IE of Py(OC3) is lower than that of Py (756.4 vs 780.6 kJ mol^–1^). The structures and IR spectra computed for further higher-energy isomers of Py_2_(OC)^+^ are shown in Figures S17 and S18. These are not discussed further because of their (slightly) higher energy.

The computed IR spectra of the proposed Py_2_^+^ isomers are compared in Figure 7 to the IRPD spectrum of Py_2_^+^. Although according to the calculations, all isomers considered in this figure may contribute to the measured spectrum, only two are required to explain all transitions observed, namely Py_2_^+^(a) and Py_2_(OC1 and/or OC2)^+^(I). Peaks A, C, and E are readily assigned to ν_NH_^f^, ν_CH_, and ν_CH2_ vibrations, respectively. The broad and strongly blue-shaded peak D shows the characteristic band profile of a H-bonded proton donor stretch mode involved in an ionic XH···Y H-bond,^50,62^ in our case ν_NH_^b^ of a NH···N H-bond. Such ν_NH_^b^ transitions occur often in Fermi resonance (FR) with the corresponding NH bending overtone (2β_NH_),^53,74,75^ or couple with ν_CH_ fundamentals (local mode coupling), which may also contribute to peak E. From our proposed structures, Py_2_(OC1)^+^(I) and Py_2_(OC2)^+^(I) are the most reasonable candidates for the peaks D and E, when considering that the scaled harmonic frequencies slightly overestimate the ν_NH_^b^ redshift. Following this scenario, the isomer ratio between Py_2_^+^(a) and Py_2_(OC)^+^ is roughly estimated as 90:10 from a comparison between computed and measured relative IR intensities, indicating that the most stable Py_2_^+^(a) isomer also strongly dominates the Py_2_^+^ ion population. We note again that the blue-shaded band D with a high-frequency tail up to 3300 cm^–1^ cannot be rationalized at all by the cn1 and cc1 isomers proposed in the recent report,^43^ while it can readily be rationalized by the strongly H-bonded isomers suggested herein.

**Figure 8:**
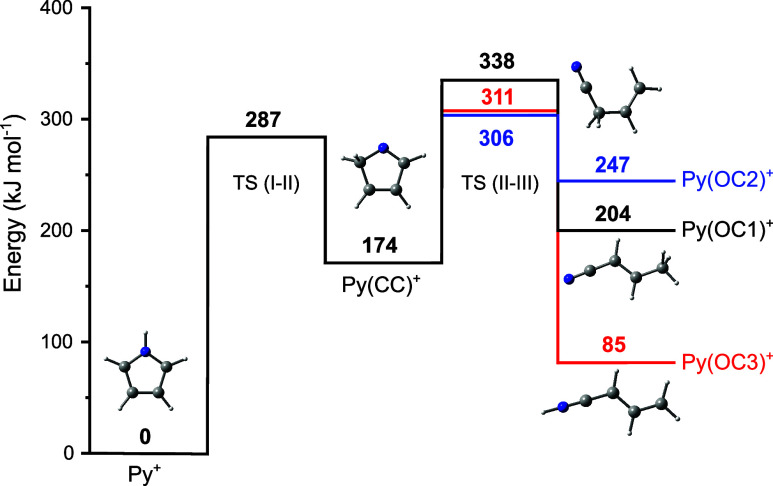
Potential energy surface of Py^+^ for H-migration and subsequent ring opening computed at the B3LYP-D3/aug-cc-pVTZ level.

To determine the barriers for the H-shift and subsequent ring-opening reactions of the Py^+^ monomer, the potential energy pathways for the formation of Py(OC1–3)^+^ are calculated (Figure 8). H-migration from N to C_α_ requires a barrier of *V*_b_ = 287 kJ mol^–1^ (2.97 eV) to form Py(CC1)^+^ at *E*_0_ = 174 kJ mol^–1^ (1.80 eV). After H-migration, further barriers of 164, 132, and 137 kJ mol^–1^ have to be surmounted to form Py(OC1)^+^, Py(OC2)^+^, and Py(OC3)^+^, respectively. Overall, total barriers of 338, 306, and 311 kJ mol^–1^ (3.50, 3.17, and 3.22 eV) have to be overcome for the Py^+^ → Py(OC1)^+^, Py^+^ → Py(OC2)^+^, and Py^+^ → Py(OC3)^+^ isomerization reactions. Such an amount of energy is readily available upon EI in our experiment (with *E* ≤ 200 eV). Because of the relatively high barriers of 3.0 eV involved in the formation of the Py(CC)^+^ and Py(OC)^+^ monomer ions and their insufficient collisional cooling in the plasma expansion, the resulting Py(CC)_2_^+^ and Py(OC)_2_^+^ dimers probably also contain a high internal energy content (limited only by their rather high binding energy), which probably prevents the formation of measurable concentrations cold Ar/N_2_-tagged species. This consideration explains why the core ions of Py_2_^+^Ar/N_2_ are strongly dominated by stacked Py_2_^+^ dimers, while the bare Py_2_^+^ clusters contain also a minor population of higher-energy isomers.

## 4 Conclusions

We analyze herein the internal energy dependence of Py_2_^+^ formation using IRPD spectroscopy of mass-selected clusters by adjusting their internal energy using various tags (none, Ar, N_2_) and variable ion source conditions. The geometries, energies, and IR spectra of various Py_2_^+^ isomers are computed at the dispersion-corrected B3LYP-D3 level to assign the IRPD spectra. The IRPD spectrum of bare Py_2_^+^ exhibits broader transitions compared to Py_2_^+^Ar and Py_2_^+^N_2_, indicating the lower internal energy of the tagged ions. The Py_2_^+^Ar/N_2_ spectra exhibit one ν_CH_ and two distinct ν_NH_ bands, indicating the exclusive presence of the most stable stacked Py_2_^+^ isomer. In contrast, IRPD spectra of bare Py_2_^+^ show two additional bands below 3100 cm^–1^, suggesting the minor presence of further less stable isomers under high-internal energy conditions. Our B3LYP-D3 calculations offer an assignment to isomers resulting from the H-shift and ring opening of Py^+^. These structures differ from those suggested previously (cc1 and cn1),^43^ which require the formation of a chemical C–C or C–N bond between the two heterocycles. The cc1 and cn1 structures, indeed, cannot provide an acceptable assignment to the intense and broad band at 3020 cm^–1^ (in particular, its blue tail), strongly questioning the previous assignment.^43^ Our results demonstrate that Ar/N_2_ tagging not only reduces the internal energy of Py_2_^+^ but also selects the most stable Py_2_^+^ structure. The applied experimental and computational approach will be further utilized to study the CR interaction of stacked Py_2_^+^ through microhydration without the interference of other high-energy isomers.

## Supporting Information Available

Additional isomeric structures, measured and computed harmonic and anharmonic IR spectra, and energies of various isomers of Py^(+)^, Py_2_^+^, and Py_2_^+^L_*n*_ (PDF)
