# Supplementary material for: Internal Energy Dependence of the Pyrrole Dimer Cation Structures Formed in a Supersonic Plasma Expansion: Charge-Resonance and Hydrogen-Bonded Isomers
Source: J Phys Chem A. 2024 May 13;128(20):3993–4006. doi: 10.1021/acs.jpca.4c01834 (PMC11129305; doi:10.1021/acs.jpca.4c01834)
Supplement: Supplementary file 1 — jp4c01834_si_001.pdf [file jp4c01834_si_001.pdf]

Supporting Information

Internal Energy Dependence of the Pyrrole Dimer Cation Structures Formed in a Supersonic Plasma Expansion: Charge-Resonance and Hydrogen-Bonded Isomers

Dashjargal Arildii,[a] Yoshiteru Matsumoto,[b] Otto Dopfer[a,c]*

[a] Institut für Optik und Atomare Physik, Technische Universität Berlin, Hardenbergstrasse 36, 10623 Berlin, Germany.

[b] Department of Chemistry, Faculty of Science, Shizuoka University, 836 Ohya, Suruga-ku, Shizuoka, 422-8529, Japan.

[c] International Research Frontiers Initiative, Tokyo Institute of Technology, 4259, Nagatsuta-cho, Midori-ku, Yokohama, 226-8503, Japan.

* Corresponding author: dopfer@physik.tu-berlin.de

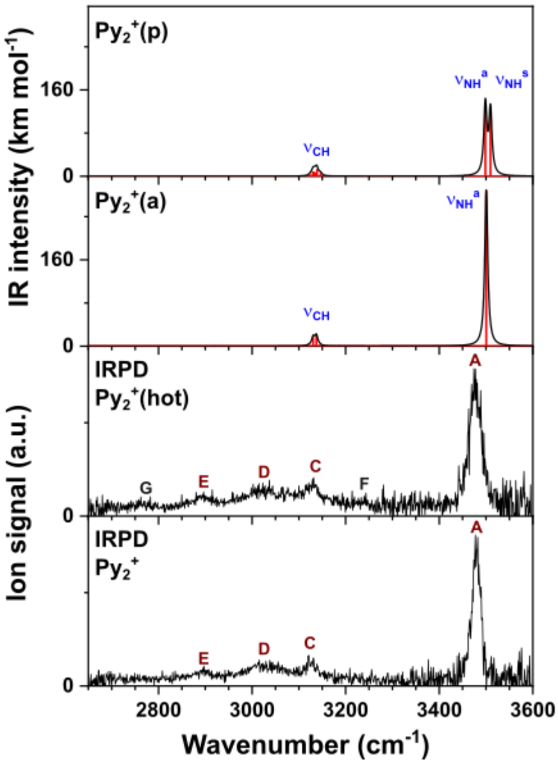

Figure S1. Comparison of IRPD spectra of Py_2^+(hot) for nozzle-out and Py_2^+ for nozzle-in conditions in the CH and NH stretch range to linear IR absorption spectra computed for the most stable isomers of Py_2^+(a/p) at the B3LYP-D3/aug-cc-pVTZ level. The positions, widths, and vibrational and isomer assignments of the transitions observed are listed in Table 1 and Table S1.

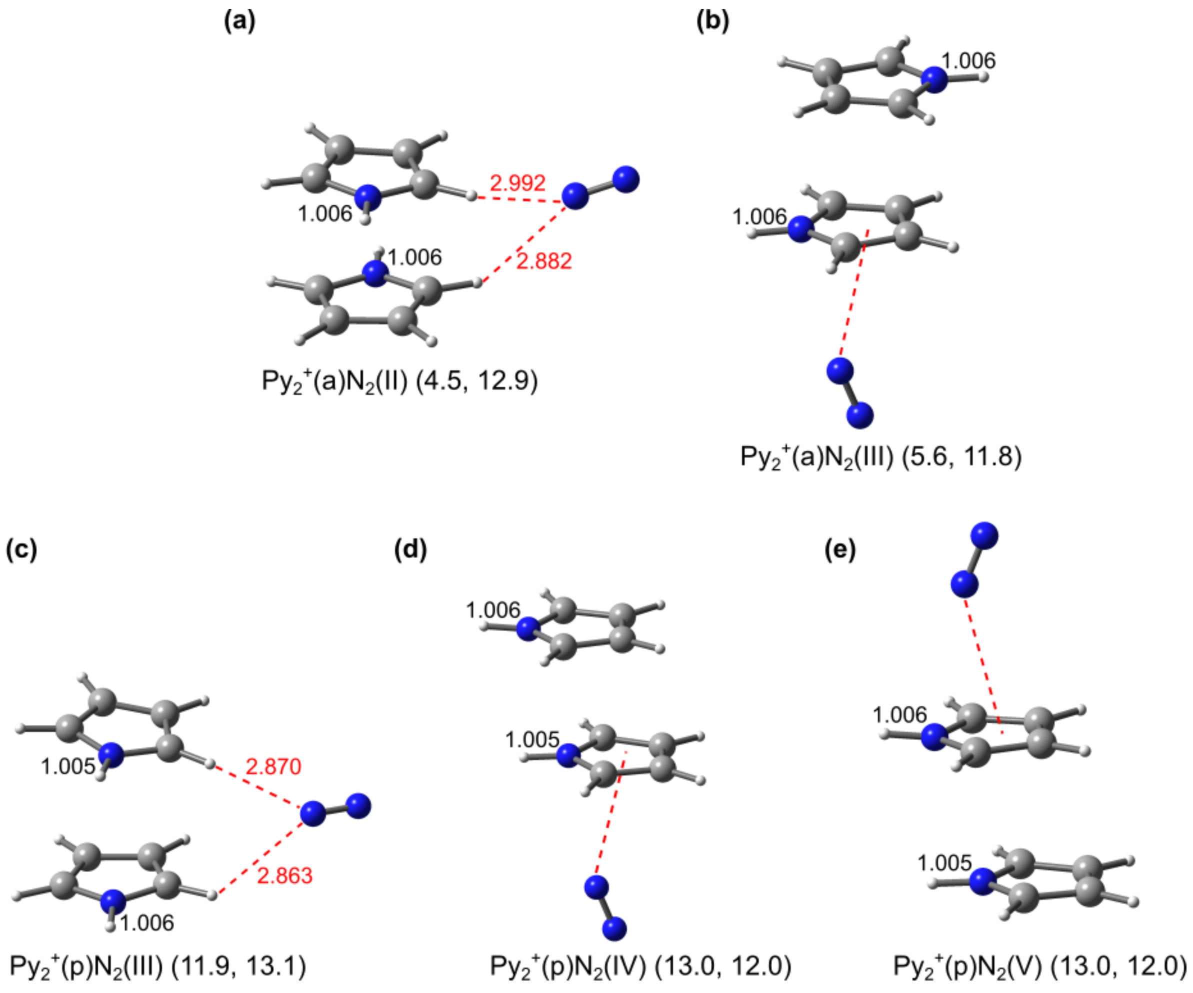

Figure S2. Optimized structures of $Py_2^+(a/p)N_2$ isomers calculated at the B3LYP-D3/aug-cc-pVTZ level. Selected intra- and intermolecular bond lengths (in Å) are indicated in black and red colors, respectively. The energies in parentheses correspond to E_0 and D_0 in kJ mol^{-1}.

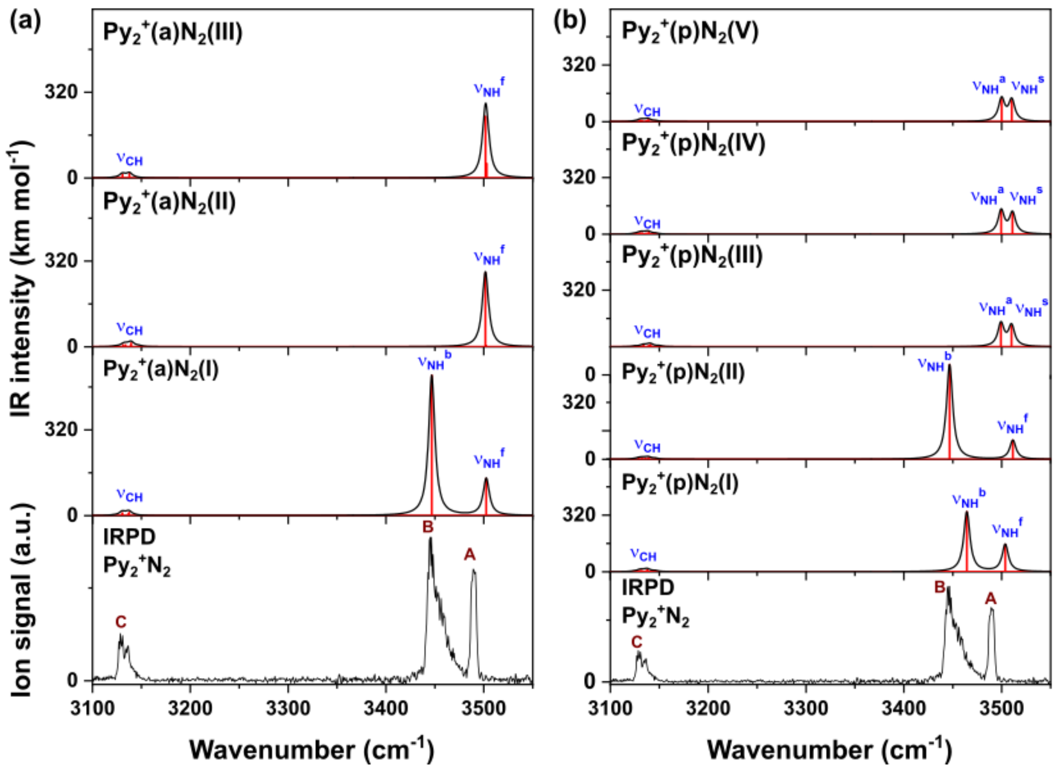

Figure S3. Comparison of IRPD spectra of $Py_2^+N_2$ in the CH and NH stretch range to linear IR absorption spectra computed for the isomers of (a) Py_2^+(a)N_2 and (b) Py_2^+(p)N_2 at the B3LYP-D3/aug-cc-pVTZ level. The positions, widths, and vibrational and isomer assignments of the transitions observed are listed in Table S2.

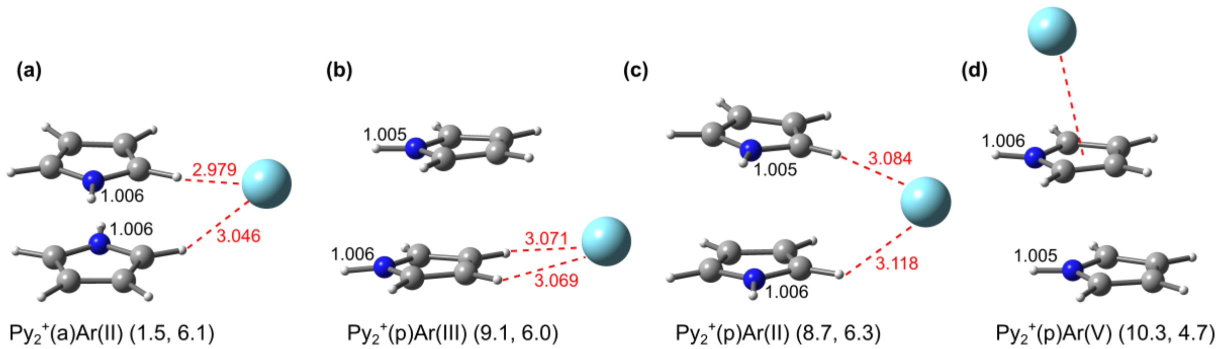

Figure S4. Optimized structures of Py_2^+(a/p)Ar isomers calculated at the B3LYP-D3/aug-cc-pVTZ level. Selected intra- and intermolecular lengths (in Å) are indicated in black and red colors, respectively. The energies in parentheses correspond to relative energies and dissociation energies of the most weakly-bonded ligand (E_0 and D_0 in kJ mol^{-1}).

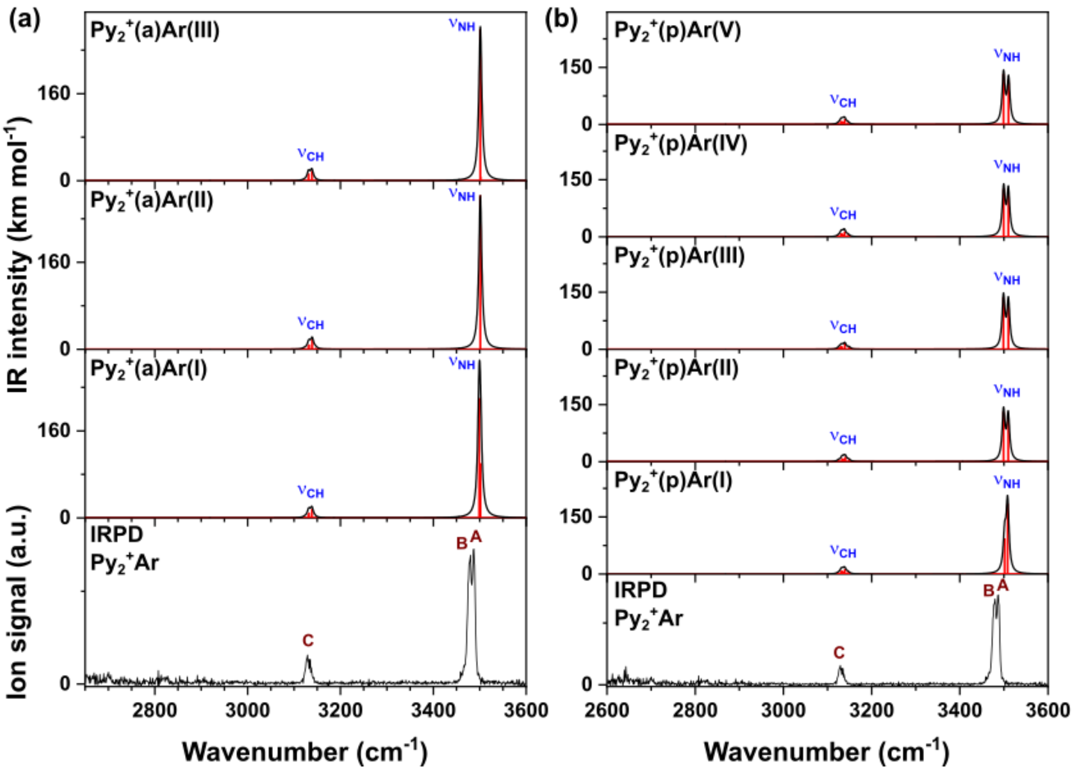

Figure S5. Comparison of IRPD spectrum of Py_2^+Ar in the CH and NH stretch range to linear IR absorption spectra computed for the isomers of (a) Py_2^+(a)Ar and (b) Py_2^+(p)Ar at the B3LYP-D3/aug-cc-pVTZ level. The positions, widths, and vibrational and isomer assignments of the transitions observed are listed in Table S2.

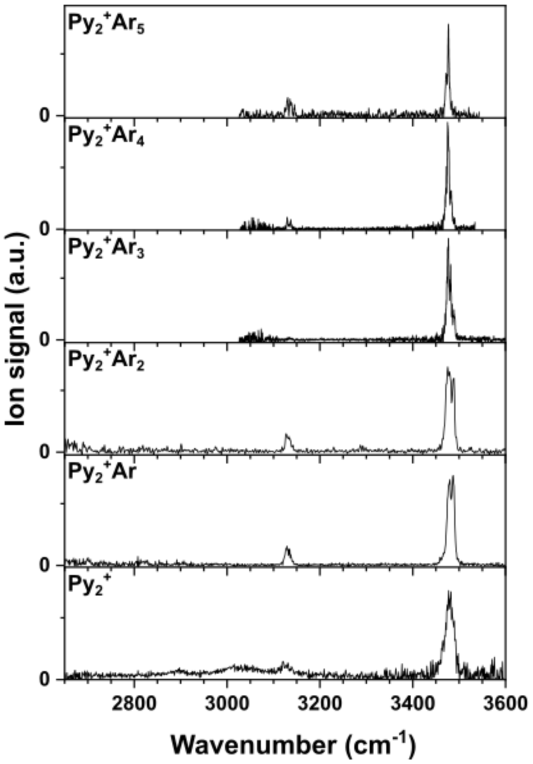

Figure S6. IRPD spectra of $Py_2^+Ar_n$ (n=0-5) are measured in the CH and NH stretch range. The positions, widths, and assignments of the transitions observed are listed in Table S3.

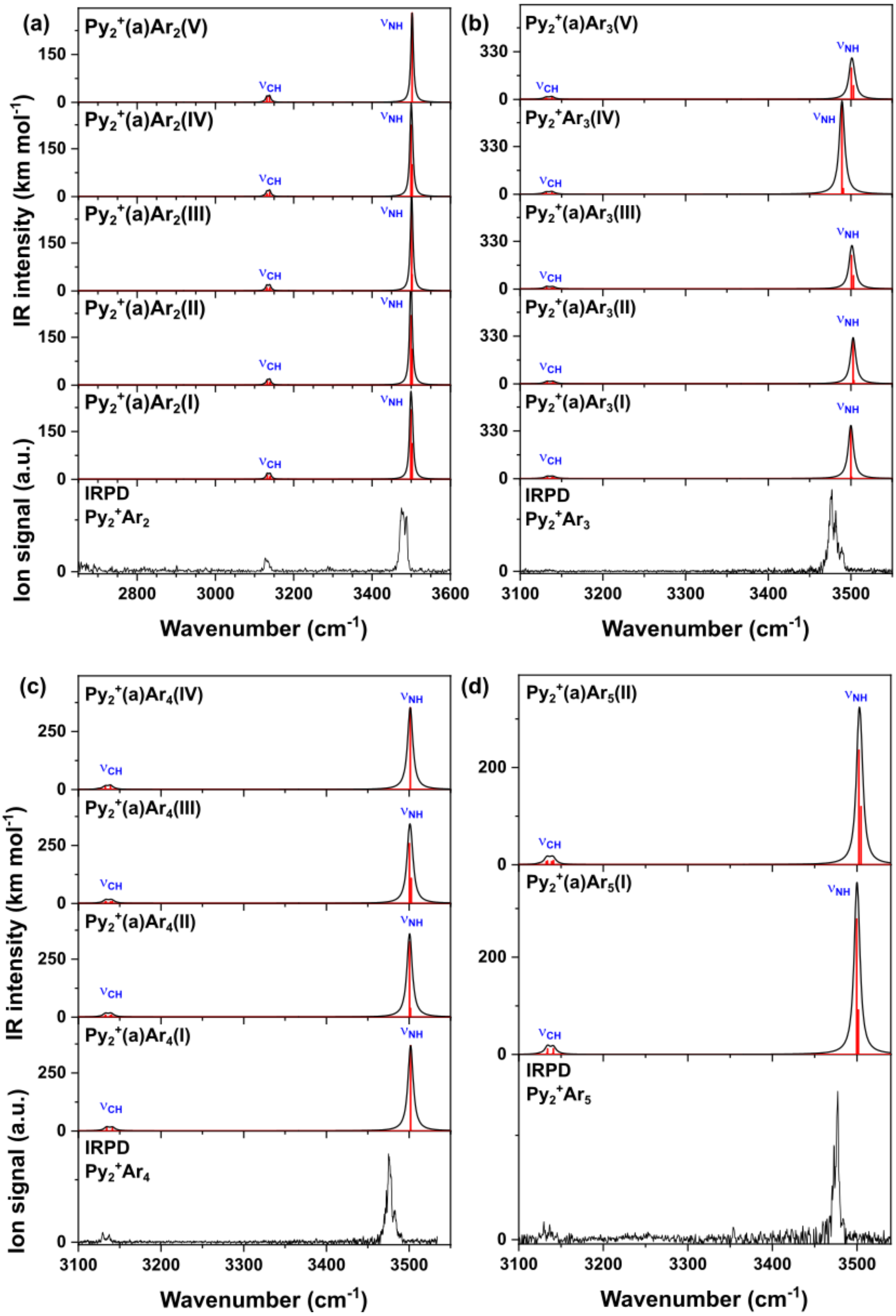

Figure S7. Comparison of IRPD spectra of $Py_2^+Ar_n$ (n=2-5) in the CH and NH stretch range to linear IR absorption spectra computed for the isomers of (a) Py_2^+(a)Ar_2, (b) Py_2^+(a)Ar_3, (c) Py_2^+(a)Ar_4, and (d) Py_2^+(a)Ar_5 at the B3LYP-D3/aug-cc-pVTZ level. The positions, widths, and vibrational and isomer assignments of the transitions observed are listed in Table S3.

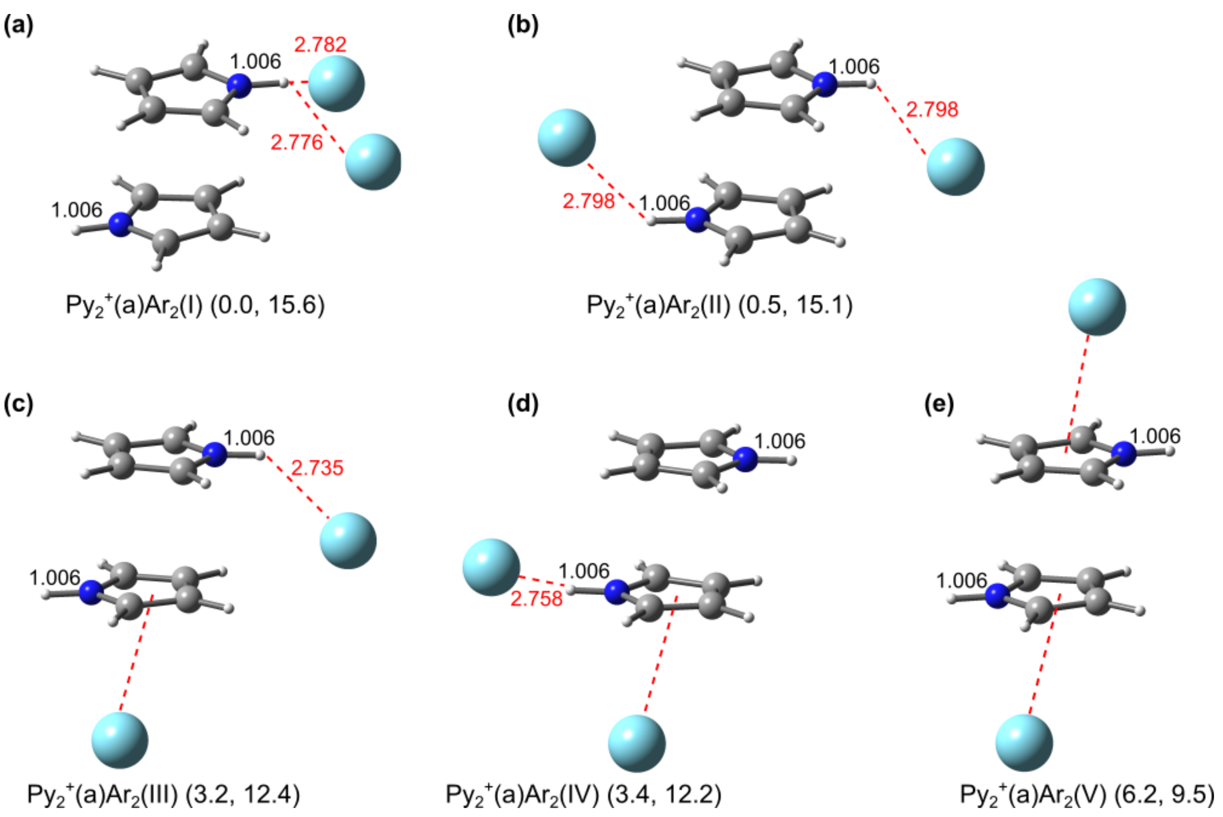

Figure S8. Optimized structures of Py_2^+(a)Ar_2 isomers calculated at the B3LYP-D3/aug-cc-pVTZ level. Selected intra- and intermolecular bond lengths (in Å) are indicated in black and red colors, respectively. The energies in parentheses correspond to E_0 and D_0 (total binding energy) in kJ mol^{-1}.

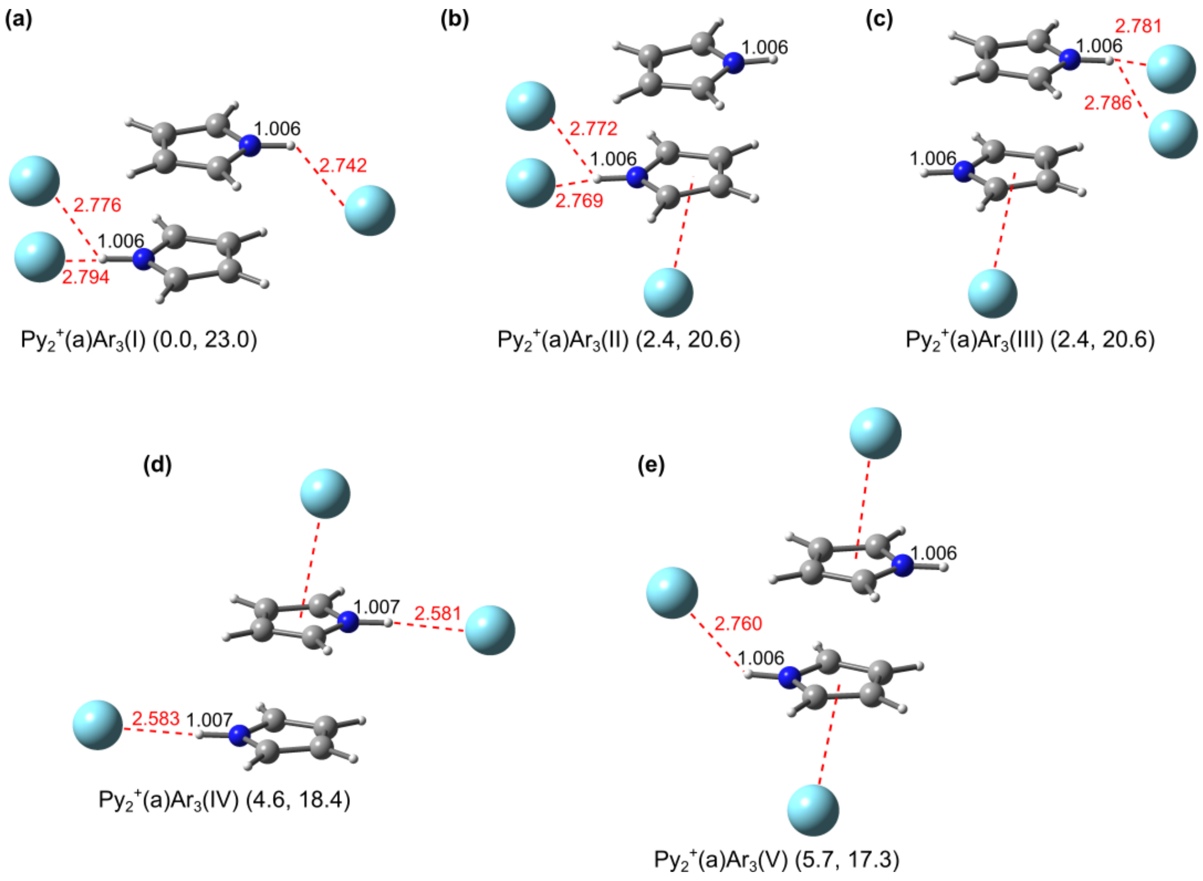

Figure S9. Optimized structures of Py_2^+(a)Ar_3 isomers calculated at the B3LYP-D3/aug-cc-pVTZ level. Selected intra- and intermolecular bond lengths (in Å) are indicated in black and red colors, respectively. The energies in parentheses correspond to E_0 and D_0 (total binding energy) in kJ mol^{-1}.

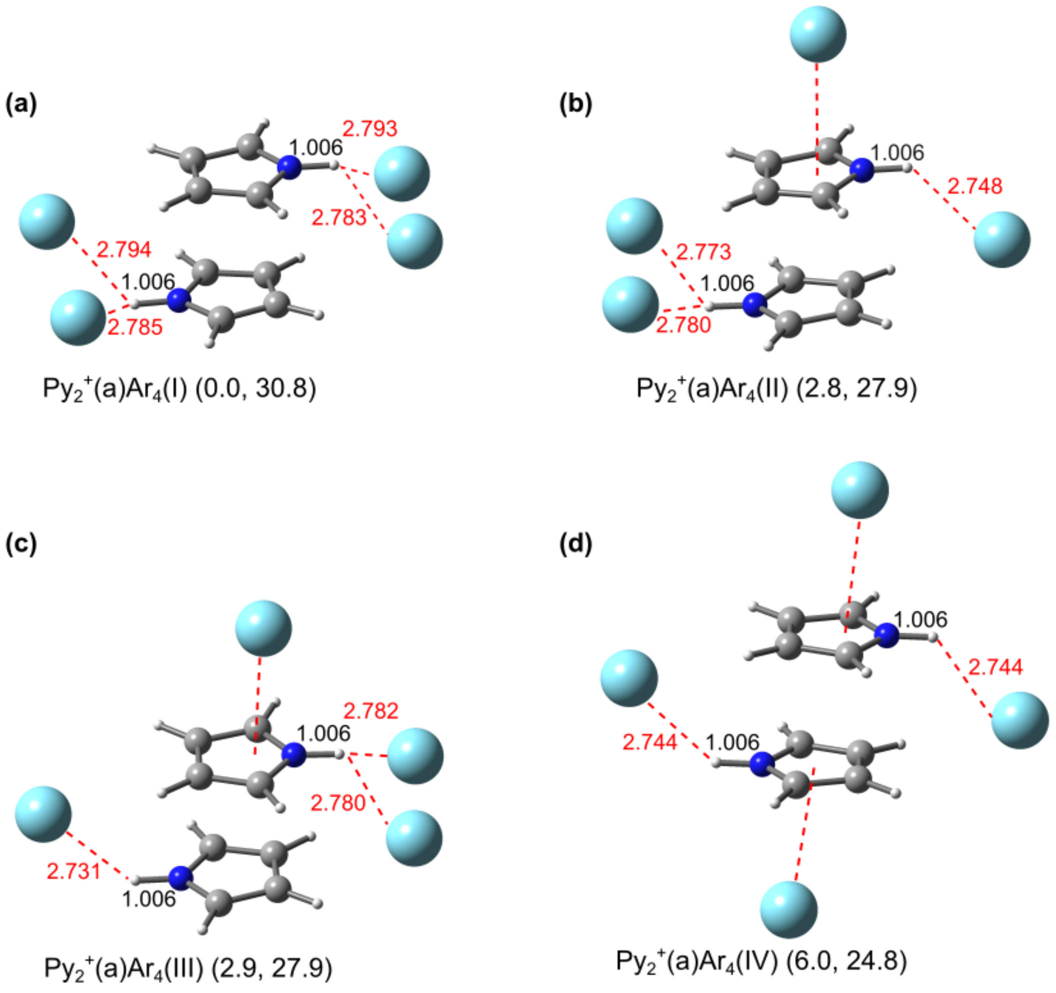

Figure S10. Optimized structures of Py_2^+(a)Ar_4 isomers calculated at the B3LYP-D3/aug-cc-pVTZ level. Selected intra- and intermolecular bond lengths (in Å) are indicated in black and red colors, respectively. The energies in parentheses correspond to E_0 and D_0 (total binding energy) in kJ mol^{-1}.

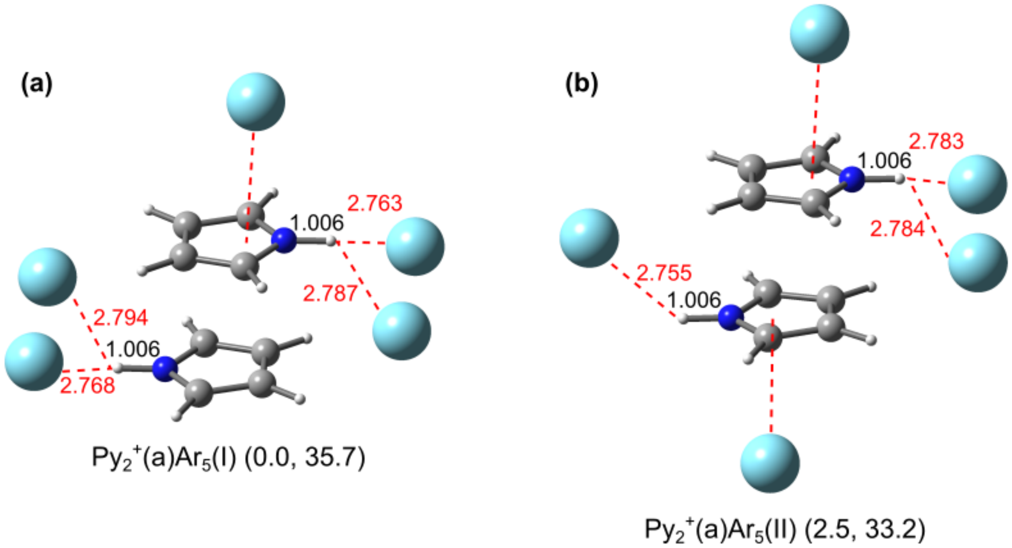

Figure S11. Optimized structures of Py_2^+(a)Ar_5 isomers calculated at the B3LYP-D3/aug-cc-pVTZ level. Selected intra- and intermolecular bond lengths (in Å) are indicated in black and red colors, respectively. The energies in parentheses correspond to E_0 and D_0 (total binding energy) in kJ mol^{-1}.

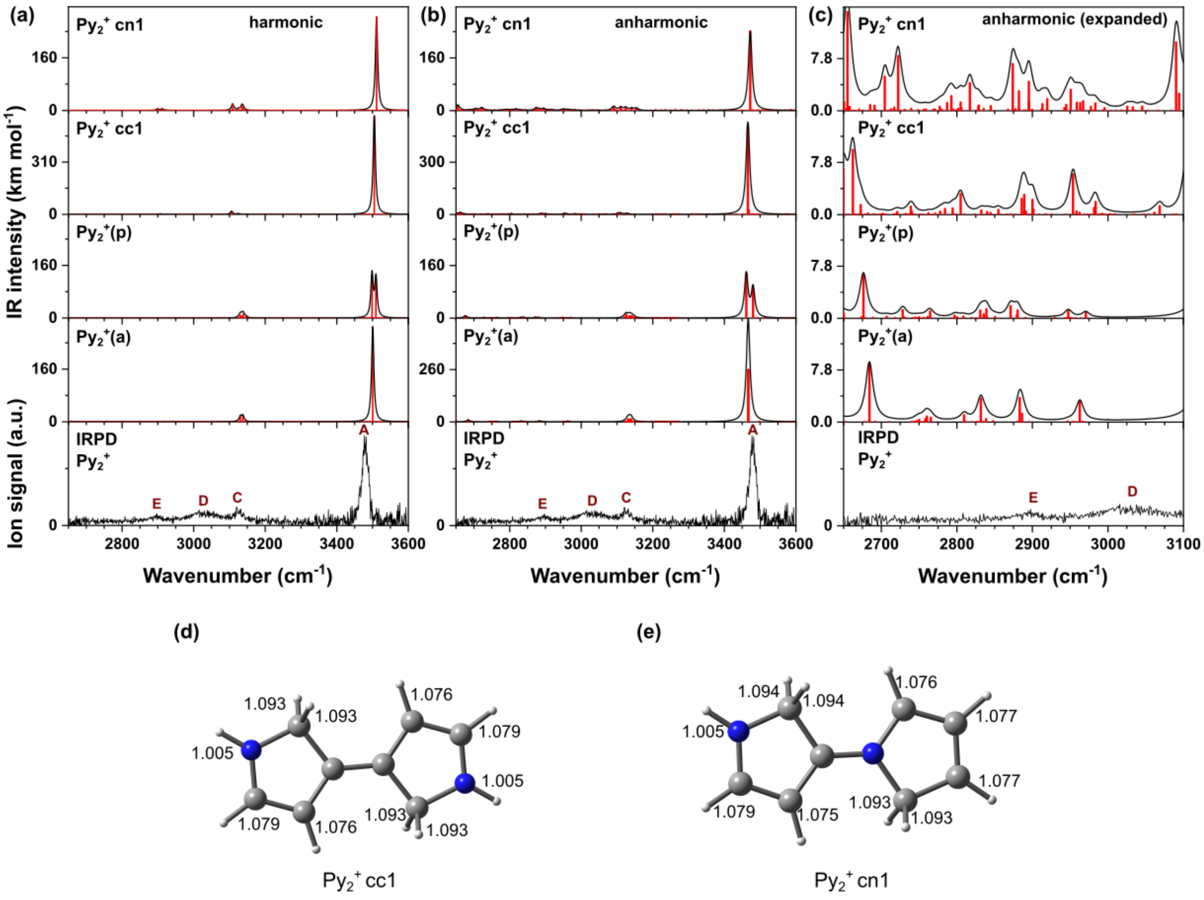

Figure S12. (a-c) Comparison of IRPD spectra of Py$_2^+$ in the CH and NH stretch range to linear IR absorption spectra computed for the isomers of Py$_2^+$(a/p), cc1, and cn1. (a) Harmonic IR absorption spectra calculated at the B3LYP-D3/aug-cc-pVTZ level, (b) anharmonic IR absorption spectra, and (c) expanded anharmonic IR absorption spectra calculated at the B3LYP-D3/aug-cc-pVDZ level. The positions, IR oscillator strength, and vibrational assignments of the harmonic transitions of cc1 and cn1 are listed in Table S4. Optimized structures of (d) cc1 and (e) cn1 isomers calculated at the B3LYP-D3/aug-cc-pVTZ level.

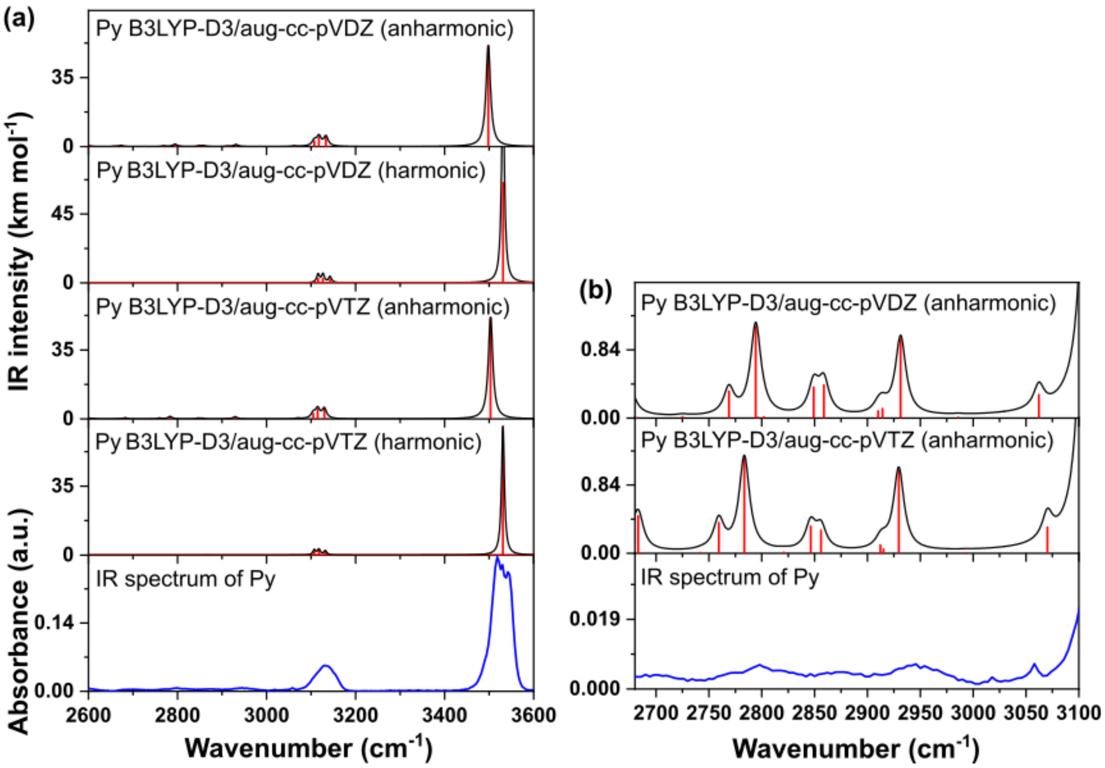

Figure S13. (a) Comparison of IR absorption spectrum of Py (taken from the NIST data base) to linear harmonic and anharmonic IR absorption spectra computed at the B3LYP-D3/aug-cc-pVTZ and B3LYP-D3/aug-cc-pVDZ levels. (b) Expanded view of (a).

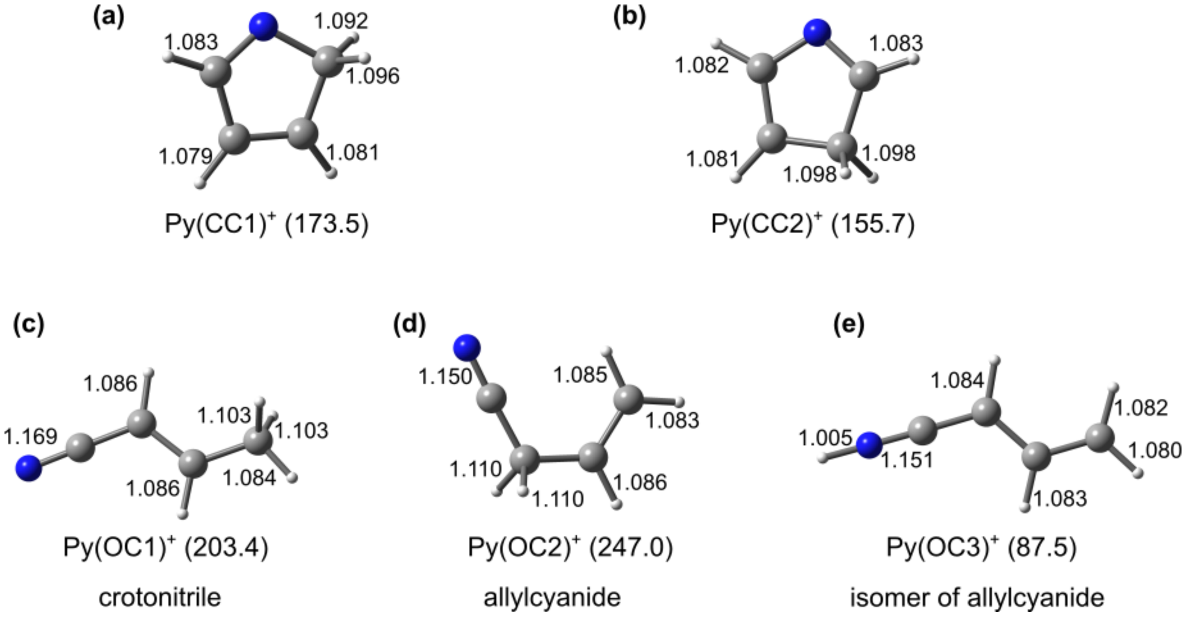

Figure S14. Optimized structures of Py^+ isomers calculated at the B3LYP-D3/aug-cc-pVTZ level. Selected intramolecular bond lengths (in Å) are indicated. E_0 energies in parentheses are given in kJ mol^{-1}.

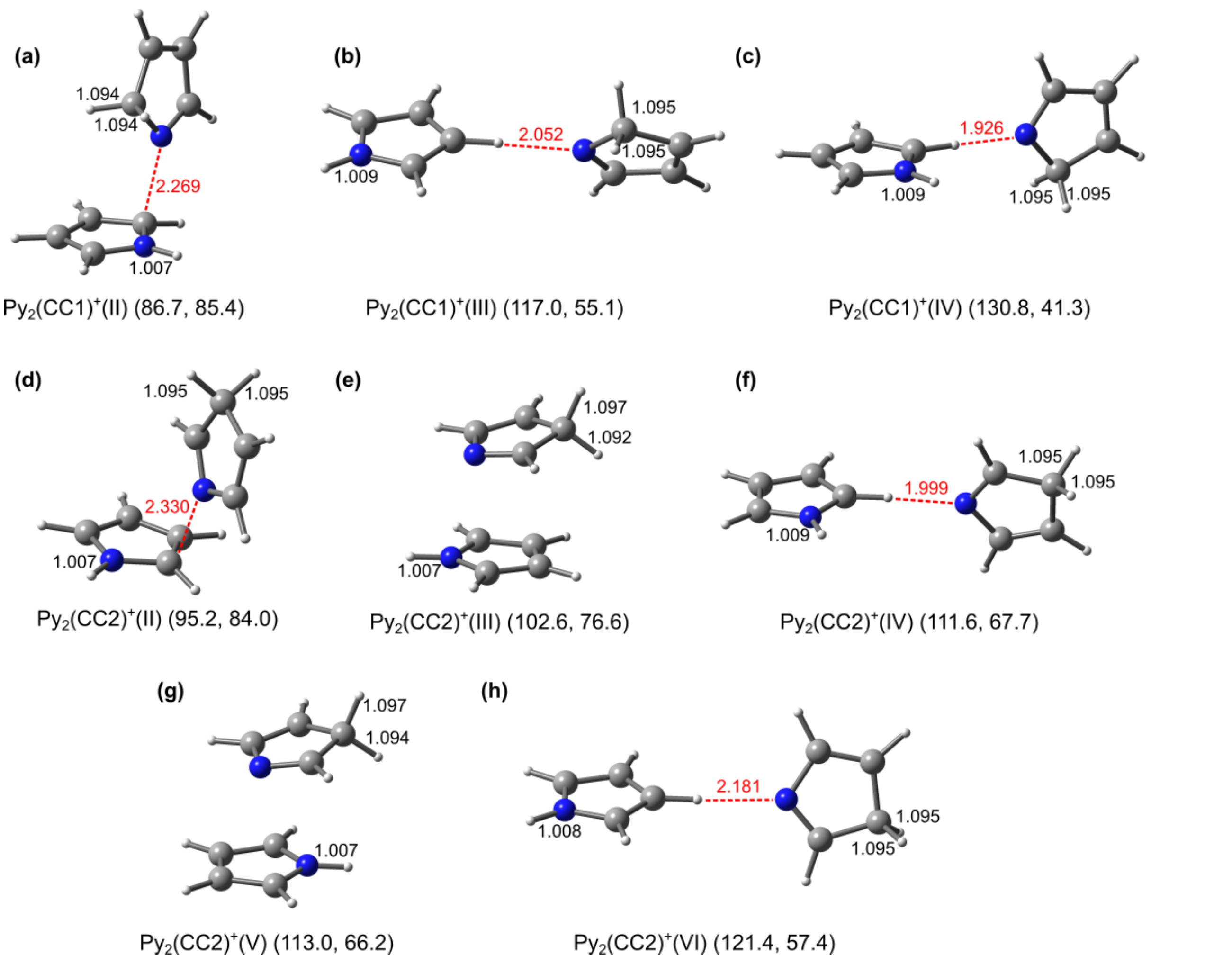

Figure S15. Optimized structures of (a-c) $Py_2(CC1)^+$ and (d-h) $Py_2(CC2)^+$ isomers calculated at the B3LYP-D3/aug-cc-pVTZ level. Selected intra- and intermolecular bond lengths (in Å) are indicated in black and red colors, respectively. The energies in parentheses correspond to E_0 and D_0 in kJ mol^{-1}.

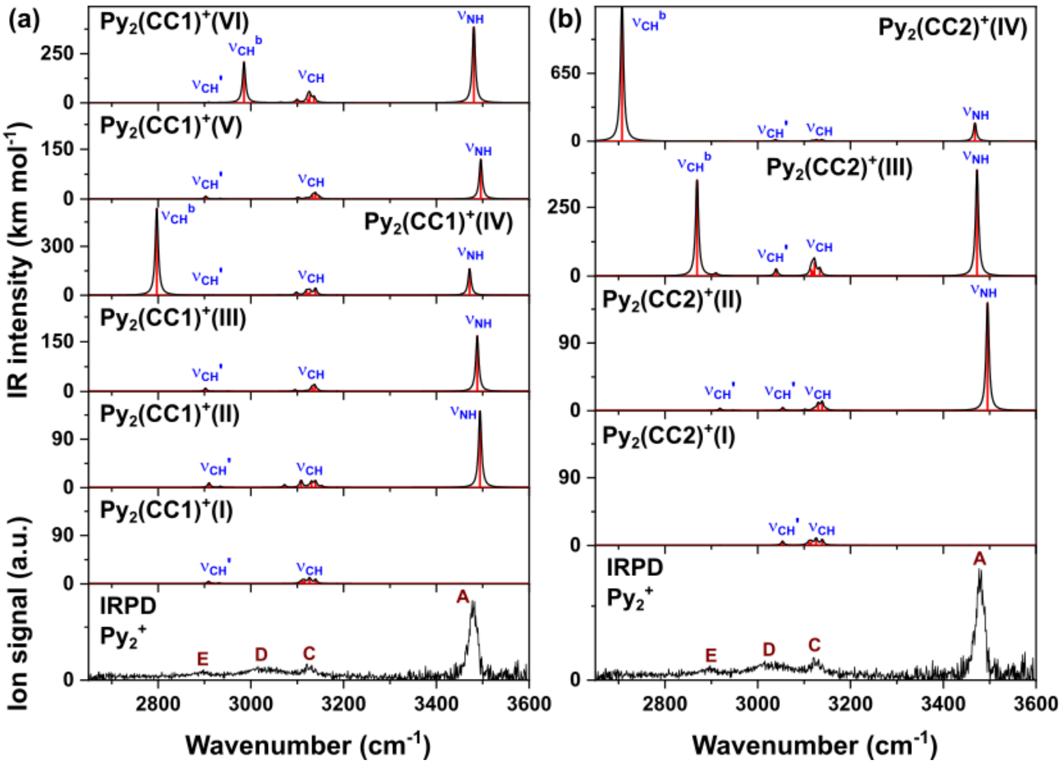

Figure S16. Comparison of IRPD spectra of Py_2^+ in the CH and NH stretch range to linear IR absorption spectra computed for the isomers of (a) $Py_2(CC1)^+$ and (b) $Py_2(CC2)^+$ at the B3LYP-D3/aug-cc-pVTZ level.

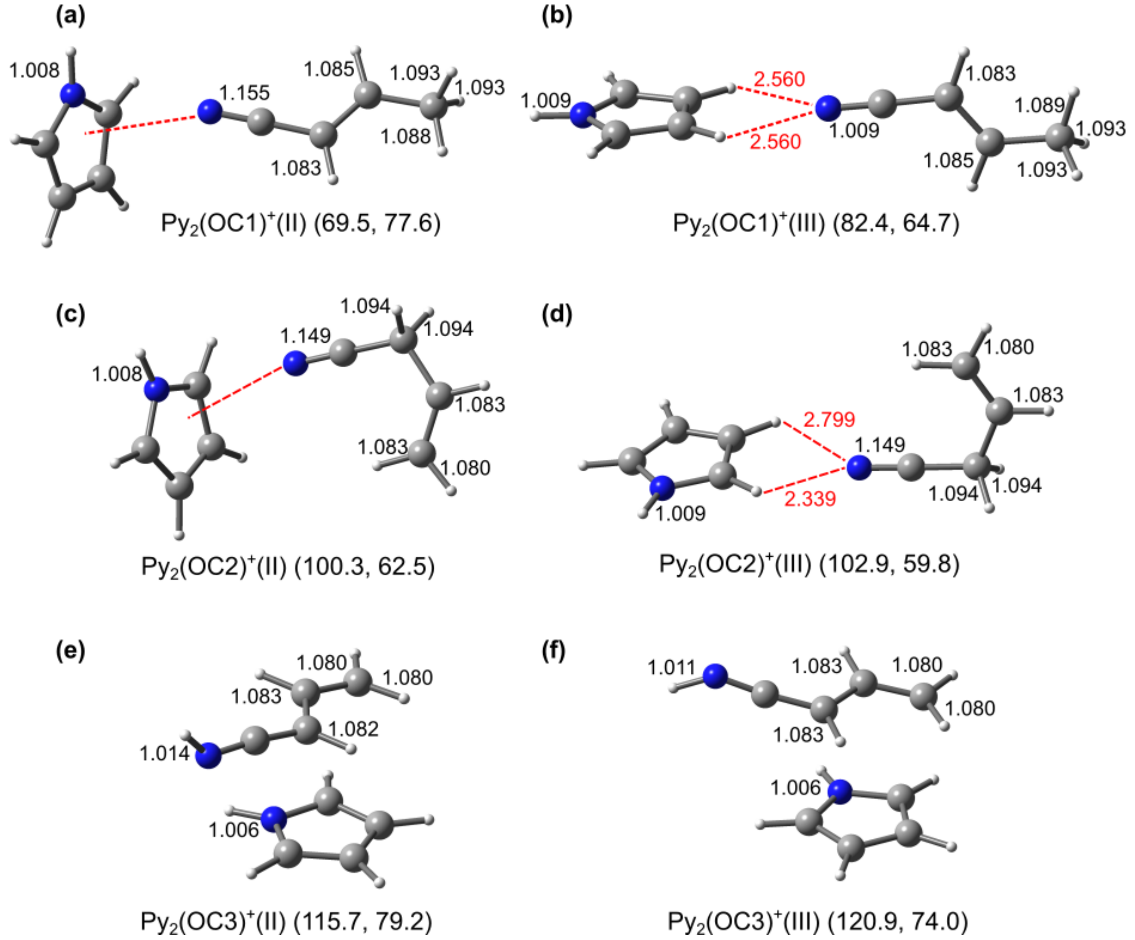

Figure S17. Optimized structures of (a-b) $Py_2(OC1)^+$, (c-d) $Py_2(OC2)^+$, and (e-f) $Py_2(OC3)^+$ isomers calculated at the B3LYP-D3/aug-cc-pVTZ level. Selected intra- and intermolecular bond lengths (in Å) are indicated in black and red colors, respectively. The energies in parentheses correspond to E_0 and D_0 in kJ mol^{-1}.

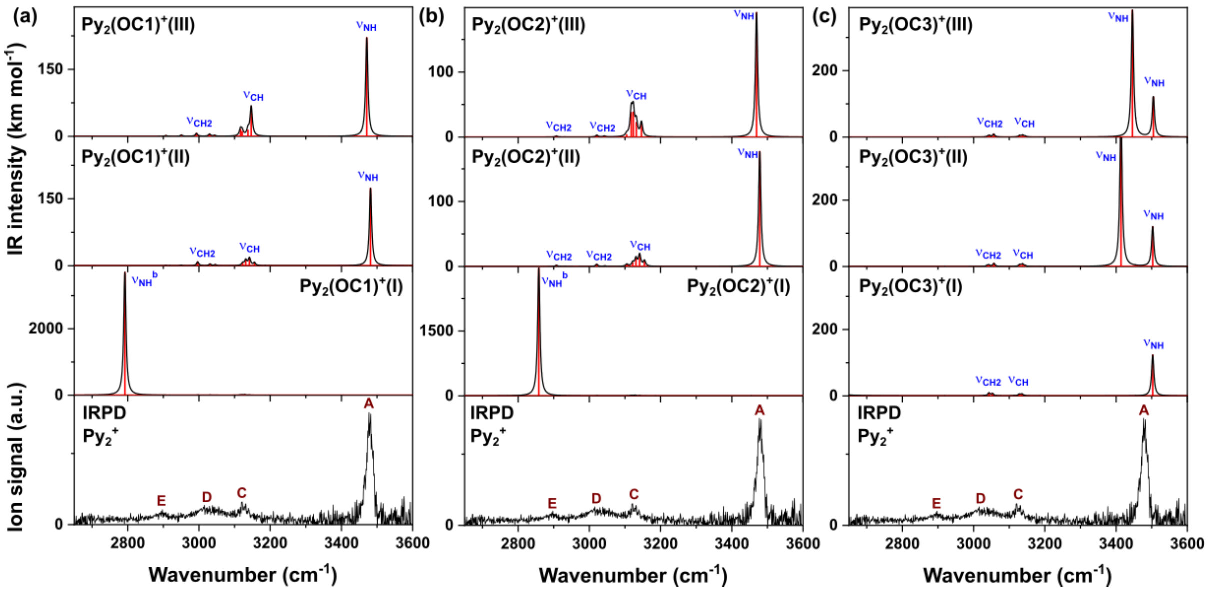

Figure S18. Comparison of IRPD spectra of Py_2^+ in the CH and NH stretch range to linear IR absorption spectra computed for the isomers of (a) $Py_2(OC1)^+$, (b) $Py_2(OC2)^+$, and $Py_2(OC3)^+$ at the B3LYP-D3/aug-cc-pVTZ level.

Table S1. Peak positions (cm^{-1}) and integrated areas normalized to 1 of the deconvoluted IRPD spectra of Py_2^+(hot) in nozzle-out, Py_2^+ in nozzle-in conditions, $Py_2^+Ar_n$ (n=1-2), and $Py_2^+N_2$ using Lorentzian line profiles.

**Py_2^+(hot)**		**Py_2^+**		**Py_2^+Ar**		**$Py_2^+Ar_2$**		**$Py_2^+N_2$**	
Position[a]	Area	Position[a]	Area	Position[a]	Area	Position[a]	Area	Position[a]	Area
3477	0.45	3479	0.51	3482	0.88	3479	0.89	3490	0.84
3128	0.11	3126	0.09	3130	0.12	3131	0.11	3445	
3026	0.29	3028	0.32					3133	0.16
2891	0.06	2888	0.08						
3236	0.06								
2756	0.04								

[a]The peak positions are the center of the deconvoluted peaks.

Table S2. IR frequencies and intensities (in parenthesis, km mol^{-1}) of the additional isomers of Py_2^+(a/p)Ar and Py_2^+(a/p)N_2 calculated at the B3LYP-D3/aug-cc-pVTZ level.

structure	calc (cm^{-1})	assignment
Py_2^+(a)N_2(II)	3502 (5)	ν_{NH}^{s}
	3501 (276)	ν_{NH}^{a}
	3140 (14)	ν_{CH}
Py_2^+(a)N_2(III)	3503 (54)	ν_{NH}^{s}
	3502 (230)	ν_{NH}^{a}
	3138 (16)	ν_{CH}
Py_2^+(p)N_2(III)	3510 (117)	ν_{NH}^{s}
	3499 (130)	ν_{NH}^{a}
	3141 (12)	ν_{CH}
Py_2^+(p)N_2(IV)	3511 (117)	ν_{NH}^{s}
	3500 (132)	ν_{NH}^{a}
	3139 (11)	ν_{CH}
Py_2^+(p)N_2(V)	3510 (119)	ν_{NH}^{s}
	3500 (127)	ν_{NH}^{a}
	3139 (12)	ν_{CH}
Py_2^+(a)Ar(II)	3502 (1)	ν_{NH}^{s}
	3501 (282)	ν_{NH}^{a}
	3139 (17)	ν_{CH}
Py_2^+(p)Ar(II)	3510 (119)	ν_{NH}^{s}
	3499 (130)	ν_{NH}^{a}
	3140 (10)	ν_{CH}
Py_2^+(p)Ar(III)	3510 (123)	ν_{NH}^{s}
	3499 (134)	ν_{NH}^{a}
	3139 (14)	ν_{CH}
Py_2^+(p)Ar(V)	3510 (116)	ν_{NH}^{s}
	3499 (131)	ν_{NH}^{a}
	3139 (12)	ν_{CH}

Table S3. Positions and suggested vibrational assignments of the transitions observed in the IRPD spectra of $Py_2^+Ar_n$ (n=2-5) compared to calculated IR frequencies of the most stable isomers at the B3LYP-D3/aug-cc-pVTZ level.

	exp (cm^{-1})^a	structure	calc (cm^{-1})^b	assignment
$Py_2^+Ar_2$	3488 (7)<br>3479 (13)<br>3475 (10)<br>3131 (11)	Py_2^+(a)Ar_2(I)	3503 (112)	ν_{NH}^{s}
			3498 (218)	ν_{NH}^{a}
			3132 (13)	ν_{CH}
		Py_2^+(a)Ar_2(II)	3503 (112)	ν_{NH}^{s}
			3499 (218)	ν_{NH}^{a}
			3132 (13)	ν_{CH}
		Py_2^+(a)Ar_2(III)	3502 (51)	ν_{NH}^{s}
			3501 (258)	ν_{NH}^{a}
			3131 (10)	ν_{CH}
		Py_2^+(a)Ar_2(IV)	3502 (99)	ν_{NH}^{s}
			3500 (223)	ν_{NH}^{a}
			3139 (14)	ν_{CH}
		Py_2^+(a)Ar_2(V)	3502 (279)	ν_{NH}^{a}
			3138 (19)	ν_{CH}
$Py_2^+Ar_3$	3489 (7)<br>3481 (3)<br>3476 (6)<br>3135 (22)	Py_2^+(a)Ar_3(I)	3501 (10)	ν_{NH}^{s}
			3500 (359)	ν_{NH}^{a}
			3140 (9)	ν_{CH}
		Py_2^+(a)Ar_3(II)	3504 (22)	ν_{NH}^{s}
			3503 (300)	ν_{NH}^{a}
			3133 (11)	ν_{CH}
		Py_2^+(a)Ar_3(III)	3503 (92)	ν_{NH}^{s}
			3501 (232)	ν_{NH}^{a}
			3132 (15)	ν_{CH}
		Py_2^+(a)Ar_3(IV)	3491 (39)	ν_{NH}^{s}
			3489 (612)	ν_{NH}^{a}
			3138 (15)	ν_{CH}
		Py_2^+(a)Ar_3(V)	3503 (95)	ν_{NH}^{s}
			3501 (217)	ν_{NH}^{a}
			3138 (15)	ν_{CH}
$Py_2^+Ar_4$	3489 (6)<br>3482 (5)<br>3476 (6)<br>3473 (6)<br>3137 (5)<br>3129 (3)	Py_2^+(a)Ar_4(I)	3503 (1)	ν_{NH}^{s}
			3502 (367)	ν_{NH}^{a}
			3141 (14)	ν_{CH}
		Py_2^+(a)Ar_4(II)	3502 (40)	ν_{NH}^{s}
			3500 (325)	ν_{NH}^{a}
			3140 (9)	ν_{CH}
		Py_2^+(a)Ar_4(III)	3503 (108)	ν_{NH}^{s}
			3500 (258)	ν_{NH}^{a}
			3141 (9)	ν_{CH}
		Py_2^+(a)Ar_4(IV)	3502 (0.02)	ν_{NH}^{s}
			3501 (353)	ν_{NH}^{a}
			3139 (16)	ν_{CH}
$Py_2^+Ar_5$	3484 (3)<br>3477 (4)<br>3473 (7)<br>3145 (3)<br>3137 (4)<br>3130 (5)	Py_2^+(a)Ar_5(I)	3502 (91)	ν_{NH}^{s}
			3500 (278)	ν_{NH}^{a}
			3141 (12)	ν_{CH}
		Py_2^+(a)Ar_5(II)	3505 (119)	ν_{NH}^{s}
			3502 (236)	ν_{NH}^{a}
			3141 (9)	ν_{CH}

^{a}Widths are in parenthesis (FWHM in cm^{-1}). ^{b}IR intensities in parenthesis (in km mol^{-1}).

Table S4. Calculated IR frequencies (cm^{-1}) and intensities ($km\ mol^{-1}$) of cc1 and cn1 structures at the B3LYP-D3/aug-cc-pVTZ level compared to available literature values.

	This work (cm^{-1})	Ref. Wei et al. (cm^{-1})[a]	assignment
cc1	3505 (583.8)	3473 (607.4)	ν_{NH}
	3125 (4.4)	3095 (4.8)	ν_{CH}
	3105 (18.0)	3077 (18.4)	ν_{CH}
	2936 (1)	2911 (1.5)	ν_{CH}
	2907 (3)	2881 (3.2)	ν_{CH}
cn1	3511 (284.6)	3479 (296.1)	ν_{NH}
	3139 (6.8), 3135 (14.7), 3126 (4.5)	3108 (6.9), 3106 (14.1), 3095 (4.9)	ν_{CH}
	3112 (2), 3109 (19)	3082 (1.5), 3080 (19.9)	ν_{CH}
	2944 (0.2), 2929 (0.4), 2912 (5), 2899 (5)	2920 (0.4), 2904 (0.6), 2886 (5.6), 2873 (5.5)	ν_{CH}

[a] Chen, X.; Wei, C.; Xie, M.; Hu, Y. Single-Photon Ionization Induced New Covalent Bond Formation in Acrylonitrile(AN)-Pyrrole(Py) Clusters. J. Phys. Chem. A 2023, 127 (40), 8272–8279. (The harmonic frequencies were calculated at the B3LYP-D3(BJ)/6-311++G(d,p) level and scaled by a factor of 0.954.

Table S5. Ionization energies of Py isomers calculated at the B3LYP-D3/aug-cc-pVTZ level.

Name	IE ($kJ\ mol^{-1}$)	IE (eV)	Ref. (eV)
Py	780.6	8.09	8.21[a]
Py(CC1)	889.3	9.22	
Py(CC2)	864.4	8.96	
Py(OC1)	944.3	9.79	10.20[b]
Py(OC2)	972.2	10.08	10.20[a]
Py(OC3)	756.4	7.84	

[a] Linstrom, P. J.; Mallard W. G., Eds. NIST Chemistry WebBook, NIST Standard Reference Database Number 69; National Institute of Standards and Technology, Gaithersburg MD, 20899.
[b] Sun, J.; Gruetzmacher, H.-F.; Lifshitz, C. Gas Phase Reactions of Carbon Cluster Ions with Crotononitrile. J. Phys. Chem. Lett. 1994, 98 (17), 4536–4542.

Cartesian coordinates (Å) and energies (hartree) of all relevant structures (B3LYP-D3/aug-cc-pVTZ)

N_2

1	7	0	-1.32796	1.34761	0.27829
2	7	0	-0.31242	1.498716	-0.09114

Sum of electronic and zero-point Energies= -109.565010
Sum of electronic and thermal Energies= -109.562650
Sum of electronic and thermal Enthalpies= -109.561706
Sum of electronic and thermal Free Energies= -109.583435

Ar

1	18	0	0	0	0

Sum of electronic and zero-point Energies= -527.560002
Sum of electronic and thermal Energies= -527.558586
Sum of electronic and thermal Enthalpies= -527.557642
Sum of electronic and thermal Free Energies= -527.575214

Py_2^+(a)

1	6	0	1.562634	0.60234	1.112731
2	6	0	1.562634	-0.73394	0.696537
3	6	0	1.562634	-0.73394	0.696537
4	6	0	1.562634	0.60234	1.112731
5	7	0	1.580394	1.38897	0.000000
6	1	0	1.594577	2.395019	0.000000
7	1	0	1.59127	1.024466	2.102133
8	1	0	1.591628	-1.58615	1.352511
9	1	0	1.591628	-1.58615	1.352511
10	1	0	1.59127	1.024466	2.102133
11	6	0	-1.56263	-0.60234	1.112731
12	6	0	-1.56263	0.733936	0.696537
13	6	0	-1.56263	0.733936	0.696537
14	6	0	-1.56263	-0.60234	1.112731
15	7	0	-1.58039	-1.38897	0.000000
16	1	0	-1.59458	-2.39502	0.000000
17	1	0	-1.59127	-1.02447	2.102133
18	1	0	-1.59163	1.586152	1.352511
19	1	0	-1.59163	1.586152	1.352511
20	1	0	-1.59127	-1.02447	2.102133

Sum of electronic and zero-point Energies= -420.090494
Sum of electronic and thermal Energies= -420.081137
Sum of electronic and thermal Enthalpies= -420.080193
Sum of electronic and thermal Free Energies= -420.126263

Py_2^+(p)

1	6	0	1.449062	1.110809	-0.53758
2	6	0	2.04218	0.69853	0.66615
3	6	0	2.045638	-0.68926	0.672157
4	6	0	1.454548	-1.11478	-0.52775
5	7	0	1.122216	-0.00583	-1.24542
6	1	0	0.716937	-0.0109	-2.16543
7	1	0	1.301201	2.097758	-0.93954
8	1	0	2.420248	1.357439	1.428171
9	1	0	2.426752	-1.33962	1.439977
10	1	0	1.310809	-2.10587	-0.92094
11	6	0	-1.74304	1.113735	-0.08667
12	6	0	-1.20324	0.701513	1.132771
13	6	0	-1.20289	-0.69428	1.137806
14	6	0	-1.74241	-1.11547	-0.07851
15	7	0	-2.06201	-0.00352	-0.80696
16	1	0	-2.54561	-0.00684	-1.68938
17	1	0	-1.95076	2.101685	-0.4602
18	1	0	-0.8757	1.357354	1.919511
19	1	0	-0.8749	-1.34422	1.929236
20	1	0	-1.94945	-2.10617	-0.44505

Sum of electronic and zero-point Energies= -420.087601
Sum of electronic and thermal Energies= -420.078127
Sum of electronic and thermal Enthalpies= -420.077182
Sum of electronic and thermal Free Energies= -420.124899

Py_2^+(a)N_2(I)

1	7	0	1.1896	-1.42772	0.120018
2	1	0	2.195175	-1.51109	0.125644
3	6	0	0.407325	-1.26156	1.222012
4	6	0	0.404875	-1.44058	-0.99236
5	6	0	-0.92794	-1.19577	0.801932
6	6	0	-0.92956	-1.30772	-0.58519
7	1	0	0.827039	-1.24008	2.212681
8	1	0	0.822202	-1.57845	-1.97464
9	1	0	-1.77879	-1.10715	1.454312
10	1	0	-1.78191	-1.32527	-1.2414
11	7	0	-1.32418	1.927752	-0.14218
12	1	0	-2.32578	2.021741	-0.14435
13	6	0	-0.53569	1.934903	0.969766
14	6	0	-0.54773	1.755992	-1.24914
15	6	0	0.792795	1.792372	0.556666
16	6	0	0.785334	1.680275	-0.83274
17	1	0	-0.94894	2.077273	1.953003
18	1	0	-0.97169	1.738251	-2.23795
19	1	0	1.648311	1.802793	1.208793
20	1	0	1.633793	1.585302	-1.48726
21	7	0	5.461268	-1.91638	0.149158
22	7	0	4.383925	-1.74925	0.139022

Sum of electronic and zero-point Energies= -529.659812
Sum of electronic and thermal Energies= -529.646490
Sum of electronic and thermal Enthalpies= -529.645546
Sum of electronic and thermal Free Energies= -529.704911

Py_2^+(a)N_2(II)

1	7	0	-0.83577	1.834412	0.902392
2	1	0	-0.62707	2.132155	1.840421
3	6	0	0.071022	1.727333	-0.10843
4	6	0	-2.06489	1.450414	0.454007
5	6	0	-0.60973	1.291982	-1.25239
6	6	0	-1.94572	1.116449	-0.89885
7	1	0	1.106017	1.985559	0.029284
8	1	0	-2.93031	1.47297	1.093139
9	1	0	-0.16422	1.155996	-2.22212
10	1	0	-2.75834	0.813433	-1.53545
11	7	0	-0.70983	-1.94893	-0.90483
12	1	0	-0.90523	-2.25461	-1.84319
13	6	0	0.495129	-1.49396	-0.46013
14	6	0	-1.61556	-1.90656	0.113318
15	6	0	0.362794	-1.18106	0.897931
16	6	0	-0.95886	-1.43785	1.255784
17	1	0	1.357707	-1.4545	-1.10143
18	1	0	-2.63093	-2.23641	-0.02169
19	1	0	1.160041	-0.8319	1.529457
20	1	0	-1.40734	-1.33334	2.228088
21	7	0	3.47301	0.341658	0.016306
22	7	0	4.557128	0.223787	-0.01504

Sum of electronic and zero-point Energies= -529.658064
Sum of electronic and thermal Energies= -529.644357
Sum of electronic and thermal Enthalpies= -529.643413
Sum of electronic and thermal Free Energies= -529.705221

Py_2^+(a)N_2(III)

1	7	0	0.605455	-2.14046	-0.01867
2	1	0	1.520308	-2.55897	-0.02551
3	6	0	-0.10166	-1.81302	1.099471
4	6	0	-0.11313	-1.80181	-1.12629
5	6	0	-1.32643	-1.27287	0.692228
6	6	0	-1.33367	-1.2661	-0.70114
7	1	0	0.278212	-2.01406	2.085958
8	1	0	0.256856	-1.99274	-2.11852
9	1	0	-2.11472	-0.95877	1.353537
10	1	0	-2.1287	-0.94545	-1.35113
11	7	0	-0.65871	1.87038	0.002066
12	1	0	-1.56516	2.306614	0.005251
13	6	0	0.056749	1.537578	1.111445
14	6	0	0.05198	1.543801	-1.11239
15	6	0	1.278298	0.997444	0.691434
16	6	0	1.275314	1.001471	-0.70078
17	1	0	-0.31427	1.734163	2.102117
18	1	0	-0.32296	1.746868	-2.10028
19	1	0	2.070252	0.677041	1.345272
20	1	0	2.064688	0.685284	-1.35977
21	7	0	2.133375	5.237619	-4.4E-05
22	7	0	1.354164	4.474125	0.002324

Sum of electronic and zero-point Energies= -529.657664
Sum of electronic and thermal Energies= -529.644035
Sum of electronic and thermal Enthalpies= -529.643091
Sum of electronic and thermal Free Energies= -529.703934

$Py_2^+(p)N_2(I)$

1	7	0	0.343248	1.972175	0.017664
2	1	0	1.296685	2.292549	0.014137
3	6	0	-0.4308	1.785901	-1.09342
4	6	0	-0.41377	1.75985	1.135808
5	6	0	-1.7164	1.446696	-0.67098
6	6	0	-1.70568	1.430274	0.725377
7	1	0	-0.039	1.940734	-2.08388
8	1	0	-0.0068	1.891545	2.123495
9	1	0	-2.5532	1.258078	-1.31949
10	1	0	-2.5326	1.226729	1.382006
11	7	0	0.288187	-1.21045	-0.0191
12	1	0	1.256343	-0.93074	-0.02003
13	6	0	-0.45935	-1.44977	1.092466
14	6	0	-0.47095	-1.41744	-1.12913
15	6	0	-1.73773	-1.85839	0.675641
16	6	0	-1.74504	-1.83825	-0.71078
17	1	0	-0.04053	-1.37524	2.080702
18	1	0	-0.06258	-1.31429	-2.11915
19	1	0	-2.5453	-2.13387	1.331126
20	1	0	-2.55947	-2.0948	-1.36548
21	7	0	3.431648	-0.44728	-0.02162
22	7	0	4.517991	-0.35324	-0.02229

Sum of electronic and zero-point Energies= -529.657123
Sum of electronic and thermal Energies= -529.643643
Sum of electronic and thermal Enthalpies= -529.642698
Sum of electronic and thermal Free Energies= -529.703749

$Py_2^+(p)N_2(II)$

1	7	0	-1.25625	-0.59668	0.014623
2	1	0	-2.13212	-0.09903	-0.04292
3	6	0	-0.58227	-1.13455	-1.04453
4	6	0	-0.6193	-0.94265	1.172078
5	6	0	0.515172	-1.84293	-0.54815
6	6	0	0.491867	-1.7231	0.840891
7	1	0	-0.93617	-1.01539	-2.0542
8	1	0	-1.00665	-0.654	2.13409
9	1	0	1.221812	-2.39159	-1.14478
10	1	0	1.176903	-2.15888	1.545976
11	7	0	1.190092	1.46004	-0.12556
12	1	0	0.351353	2.008949	-0.2003
13	6	0	1.814875	1.135697	1.040218
14	6	0	1.885209	0.941261	-1.17622
15	6	0	2.9719	0.409546	0.719542
16	6	0	3.01544	0.288433	-0.66312
17	1	0	1.447952	1.477842	1.991851

18	1	0	1.578487	1.108769	-2.19369
19	1	0	3.682281	0.028938	1.432445
20	1	0	3.766665	-0.20672	-1.25318
21	7	0	-4.09521	0.910536	-0.15057
22	7	0	-5.09964	1.330526	-0.20982

Sum of electronic and zero-point Energies= -529.656898
Sum of electronic and thermal Energies= -529.643461
Sum of electronic and thermal Enthalpies= -529.642517
Sum of electronic and thermal Free Energies= -529.702759

Py_2^+(p)N_2(III)

1	7	0	1.85547	-0.56048	-0.09865
2	1	0	2.429048	-1.08729	-0.73574
3	6	0	1.853623	0.803543	0.019256
4	6	0	1.050086	-1.09698	0.863899
5	6	0	1.024949	1.144673	1.085494
6	6	0	0.523724	-0.04391	1.618941
7	1	0	2.451566	1.422871	-0.62708
8	1	0	0.93216	-2.16017	0.977341
9	1	0	0.83616	2.145534	1.43073
10	1	0	-0.13094	-0.15698	2.464096
11	7	0	-1.01886	0.061281	-1.39696
12	1	0	-0.45822	-0.31223	-2.14315
13	6	0	-1.65197	-0.67467	-0.44741
14	6	0	-1.28218	1.38769	-1.20093
15	6	0	-2.3748	0.214953	0.370479
16	6	0	-2.14104	1.499299	-0.10314
17	1	0	-1.61819	-1.74917	-0.44153
18	1	0	-0.88595	2.142297	-1.8578
19	1	0	-2.99218	-0.07429	1.202902
20	1	0	-2.53476	2.4216	0.286503
21	7	0	-2.07102	-3.63265	2.874808
22	7	0	-1.53066	-3.12025	2.077686

Sum of electronic and zero-point Energies= -529.655292
Sum of electronic and thermal Energies= -529.641536
Sum of electronic and thermal Enthalpies= -529.640592
Sum of electronic and thermal Free Energies= -529.702985

Py_2^+(p)N_2(IV)

1	7	0	2.511159	-1.31658	-0.52797
2	1	0	2.653473	-2.17604	-1.0314
3	6	0	2.602212	-0.06308	-1.0658
4	6	0	2.326539	-1.21506	0.82271
5	6	0	2.47051	0.861785	-0.02922
6	6	0	2.298008	0.140423	1.153479
7	1	0	2.789607	0.086001	-2.11524
8	1	0	2.268845	-2.08828	1.449434
9	1	0	2.51872	1.930203	-0.14116
10	1	0	2.184858	0.534105	2.147752
11	7	0	-0.59919	-0.52547	-0.50462
12	1	0	-0.56565	-1.38145	-1.03053

13	6	0	-0.79975	-0.43868	0.838228
14	6	0	-0.53166	0.72459	-1.03805
15	6	0	-0.8851	0.922179	1.173648
16	6	0	-0.71778	1.647719	0.003138
17	1	0	-0.9255	-1.31574	1.448308
18	1	0	-0.41759	0.881653	-2.09619
19	1	0	-1.05206	1.306977	2.164493
20	1	0	-0.72661	2.717554	-0.11118
21	7	0	-4.858	0.366961	-0.5531
22	7	0	-3.82676	0.017379	-0.61939

Sum of electronic and zero-point Energies= -529.654876
Sum of electronic and thermal Energies= -529.641144
Sum of electronic and thermal Enthalpies= -529.640200
Sum of electronic and thermal Free Energies= -529.701999

Py_2^+(p)N_2(V)

1	7	0	-0.99043	0.806029	-0.97323
2	1	0	-1.31819	1.357698	-1.74817
3	6	0	-0.90494	-0.55712	-0.95997
4	6	0	-0.73619	1.285265	0.280202
5	6	0	-0.5756	-0.957	0.336485
6	6	0	-0.46982	0.197028	1.113309
7	1	0	-1.11682	-1.14218	-1.83823
8	1	0	-0.79797	2.33639	0.503406
9	1	0	-0.4527	-1.9753	0.659398
10	1	0	-0.24782	0.259369	2.163612
11	7	0	2.203645	0.510272	-0.96352
12	1	0	1.977825	1.072858	-1.76545
13	6	0	2.475812	0.991183	0.28134
14	6	0	2.30753	-0.84771	-0.96128
15	6	0	2.797067	-0.10025	1.102918
16	6	0	2.692135	-1.24714	0.327918
17	1	0	2.482577	2.046592	0.489936
18	1	0	2.164944	-1.42639	-1.85696
19	1	0	3.074156	-0.03455	2.140531
20	1	0	2.87018	-2.26297	0.634735
21	7	0	-3.94175	0.219341	0.309028
22	7	0	-4.82204	-0.08292	0.878024

Sum of electronic and zero-point Energies= -529.654865
Sum of electronic and thermal Energies= -529.641154
Sum of electronic and thermal Enthalpies= -529.640209
Sum of electronic and thermal Free Energies= -529.701801

Py_2^+(a)Ar(I)

1	7	0	-0.48877	1.683836	-0.00026
2	1	0	-1.49506	1.679799	-0.00042
3	6	0	0.296778	1.65442	1.112133
4	6	0	0.297085	1.654223	-1.11242
5	6	0	1.633875	1.640944	0.696268
6	6	0	1.634075	1.640831	-0.69618
7	1	0	-0.12541	1.684817	2.101428

8	1	0	-0.12481	1.684407	-2.10184
9	1	0	2.486402	1.660546	1.352256
10	1	0	2.486779	1.660296	-1.35194
11	7	0	2.198707	-1.53095	0.000798
12	1	0	3.204011	-1.57063	0.001442
13	6	0	1.412092	-1.49036	1.113193
14	6	0	1.413512	-1.4909	-1.1126
15	6	0	0.077024	-1.45271	0.695986
16	6	0	0.077909	-1.45296	-0.69709
17	1	0	1.832629	-1.52977	2.1029
18	1	0	1.835289	-1.531	-2.10175
19	1	0	-0.77731	-1.45355	1.349401
20	1	0	-0.77559	-1.45419	-1.35161
21	18	0	-3.42059	-0.34011	0.000036

Sum of electronic and zero-point Energies= -947.653420
Sum of electronic and thermal Energies= -947.641440
Sum of electronic and thermal Enthalpies= -947.640496
Sum of electronic and thermal Free Energies= -947.698461

Py_2^+(a)Ar(II)

1	7	0	-0.4469	1.801826	-0.09741
2	1	0	-1.45073	1.867927	-0.0847
3	6	0	0.355935	1.817022	1.002744
4	6	0	0.31859	1.663761	-1.21747
5	6	0	1.683444	1.715969	0.568789
6	6	0	1.659165	1.619714	-0.82083
7	1	0	-0.04538	1.929217	1.994447
8	1	0	-0.11895	1.645136	-2.2004
9	1	0	2.546807	1.743698	1.210029
10	1	0	2.500147	1.555246	-1.48867
11	7	0	2.156784	-1.49353	0.10931
12	1	0	3.160253	-1.56179	0.084384
13	6	0	1.407836	-1.32233	1.234263
14	6	0	1.337728	-1.53853	-0.97981
15	6	0	0.060751	-1.2879	0.854181
16	6	0	0.017557	-1.42299	-0.53162
17	1	0	1.855771	-1.27392	2.211039
18	1	0	1.727171	-1.68423	-1.97232
19	1	0	-0.76919	-1.20243	1.532925
20	1	0	-0.85491	-1.46562	-1.15964
21	18	0	0.13495	0.084	4.326238

Sum of electronic and zero-point Energies= -947.652854
Sum of electronic and thermal Energies= -947.640894
Sum of electronic and thermal Enthalpies= -947.639950
Sum of electronic and thermal Free Energies= -947.696364

Py_2^+(a)Ar(III)

1	7	0	0.784481	1.096688	0.138473
2	1	0	0.988246	2.071108	0.28373
3	6	0	0.641468	0.167998	1.124662

4	6	0	0.615666	0.500957	-1.07509
5	6	0	0.397211	-1.07023	0.518871
6	6	0	0.381203	-0.86159	-0.85794
7	1	0	0.759749	0.424816	2.162839
8	1	0	0.711016	1.054273	-1.99299
9	1	0	0.282193	-2.00295	1.042472
10	1	0	0.250637	-1.59777	-1.6315
11	7	0	-2.8211	-1.04497	-0.14091
12	1	0	-3.01463	-2.0212	-0.28843
13	6	0	-2.64579	-0.4524	1.073778
14	6	0	-2.68085	-0.11318	-1.12565
15	6	0	-2.41434	0.910945	0.859075
16	6	0	-2.43584	1.123198	-0.51766
17	1	0	-2.73381	-1.00876	1.990641
18	1	0	-2.7995	-0.36813	-2.16433
19	1	0	-2.28048	1.645273	1.633875
20	1	0	-2.322	2.057332	-1.03906
21	18	0	4.070142	-0.10279	0.001082

Sum of electronic and zero-point Energies= -947.652325
Sum of electronic and thermal Energies= -947.640284
Sum of electronic and thermal Enthalpies= -947.639340
Sum of electronic and thermal Free Energies= -947.696490

Py_2^+(p)Ar(I)

1	7	0	2.18783	-0.10397	-0.64155
2	1	0	2.70275	-0.10617	-1.50574
3	6	0	1.849979	1.011913	0.070197
4	6	0	1.795085	-1.21609	0.049096
5	6	0	1.228276	0.597914	1.250491
6	6	0	1.191709	-0.79713	1.235396
7	1	0	2.102601	1.998456	-0.27773
8	1	0	1.996245	-2.207	-0.32017
9	1	0	0.869506	1.252209	2.024786
10	1	0	0.797807	-1.44811	1.995383
11	7	0	-0.92045	-0.02466	-1.3152
12	1	0	-0.43215	-0.09916	-2.19098
13	6	0	-1.41603	-1.07034	-0.59445
14	6	0	-1.19344	1.142094	-0.67281
15	6	0	-2.0596	-0.54776	0.536258
16	6	0	-1.92214	0.832431	0.489079
17	1	0	-1.32936	-2.08521	-0.94109
18	1	0	-0.92317	2.094448	-1.09346
19	1	0	-2.5626	-1.12989	1.28842
20	1	0	-2.29761	1.552535	1.194996
21	18	0	1.219235	1.472195	-3.70157

Sum of electronic and zero-point Energies= -947.650976
Sum of electronic and thermal Energies= -947.638948
Sum of electronic and thermal Enthalpies= -947.638004
Sum of electronic and thermal Free Energies= -947.695037

Py_2^+(p)Ar(II)

1	7	0	2.048018	0.008174	-0.59058
2	1	0	2.631707	0.018349	-1.41014
3	6	0	1.642083	1.114548	0.102823
4	6	0	1.646442	-1.11448	0.078143
5	6	0	0.961488	0.683963	1.242606
6	6	0	0.963983	-0.71153	1.227219
7	1	0	1.890148	2.108001	-0.22917
8	1	0	1.898423	-2.09951	-0.27534
9	1	0	0.537577	1.325334	1.994003
10	1	0	0.542876	-1.37092	1.964473
11	7	0	-1.07033	0.010553	-1.39947
12	1	0	-0.56759	0.018513	-2.27001
13	6	0	-1.47457	-1.10842	-0.73607
14	6	0	-1.47907	1.117124	-0.71824
15	6	0	-2.19522	-0.69997	0.396807
16	6	0	-2.1979	0.687695	0.407982
17	1	0	-1.28391	-2.09403	-1.123
18	1	0	-1.29192	2.109567	-1.08911
19	1	0	-2.65133	-1.36033	1.113167
20	1	0	-2.65662	1.334763	1.134753
21	18	0	-1.37195	-0.05061	3.966662

Sum of electronic and zero-point Energies= -947.649956
Sum of electronic and thermal Energies= -947.637923
Sum of electronic and thermal Enthalpies= -947.636978
Sum of electronic and thermal Free Energies= -947.693503

Py_2^+(p)Ar(III)

1	7	0	2.021978	-0.19985	-0.71403
2	1	0	2.536045	-0.31208	-1.5717
3	6	0	1.824312	0.986544	-0.06157
4	6	0	1.524457	-1.21995	0.04585
5	6	0	1.182142	0.715035	1.145197
6	6	0	0.996762	-0.66656	1.21593
7	1	0	2.17263	1.918073	-0.47363
8	1	0	1.612341	-2.24781	-0.26
9	1	0	0.908282	1.448675	1.882298
10	1	0	0.551637	-1.22728	2.017826
11	7	0	-1.11638	0.207413	-1.29926
12	1	0	-0.66692	0.260629	-2.19695
13	6	0	-1.45147	-0.94119	-0.65636
14	6	0	-1.51099	1.281778	-0.55353
15	6	0	-2.11383	-0.58767	0.534281
16	6	0	-2.14922	0.799022	0.594152
17	1	0	-1.27532	-1.90883	-1.09089
18	1	0	-1.35543	2.290435	-0.89452
19	1	0	-2.5131	-1.28579	1.248957
20	1	0	-2.57968	1.409282	1.368628
21	18	0	-0.59031	-4.04578	1.01946

Sum of electronic and zero-point Energies= -947.650074
Sum of electronic and thermal Energies= -947.638027
Sum of electronic and thermal Enthalpies= -947.637083
Sum of electronic and thermal Free Energies= -947.693910

Py_2^+(p)Ar(IV)

1	7	0	2.04208	0.018361	-0.85233
2	1	0	2.483611	0.002146	-1.75633
3	6	0	1.706408	1.147544	-0.15861
4	6	0	1.80335	-1.07784	-0.07128
5	6	0	1.239874	0.759228	1.098046
6	6	0	1.300453	-0.63424	1.1528
7	1	0	1.853497	2.128539	-0.57656
8	1	0	2.036597	-2.07208	-0.4115
9	1	0	0.918749	1.429801	1.874948
10	1	0	1.036253	-1.26729	1.980935
11	7	0	-1.15247	-0.13155	-1.1491
12	1	0	-0.80237	-0.15214	-2.09118
13	6	0	-1.40307	-1.22754	-0.38136
14	6	0	-1.50196	0.993576	-0.46677
15	6	0	-1.95922	-0.78621	0.829661
16	6	0	-2.02083	0.59892	0.776385
17	1	0	-1.23463	-2.22465	-0.74842
18	1	0	-1.42126	1.97055	-0.90988
19	1	0	-2.27568	-1.42514	1.635464
20	1	0	-2.39542	1.267143	1.531917
21	18	0	-4.52392	-0.31588	-2.00505

Sum of electronic and zero-point Energies= -947.649469
Sum of electronic and thermal Energies= -947.637369
Sum of electronic and thermal Enthalpies= -947.636425
Sum of electronic and thermal Free Energies= -947.694243

Py_2^+(p)Ar(V)

1	7	0	2.009601	0.154488	-0.75875
2	1	0	2.519773	0.189499	-1.62526
3	6	0	1.60643	1.243126	-0.03801
4	6	0	1.748756	-0.98077	-0.04383
5	6	0	1.068021	0.787596	1.166876
6	6	0	1.15667	-0.60499	1.162688
7	1	0	1.762155	2.245224	-0.39858
8	1	0	2.029334	-1.95288	-0.41102
9	1	0	0.683301	1.415137	1.950751
10	1	0	0.854673	-1.28076	1.942716
11	7	0	-1.15081	-0.05444	-1.27416
12	1	0	-0.72405	-0.05012	-2.18438
13	6	0	-1.45148	-1.17101	-0.55323
14	6	0	-1.54293	1.052579	-0.58573
15	6	0	-2.08873	-0.76182	0.627602
16	6	0	-2.14614	0.624889	0.608013
17	1	0	-1.25355	-2.15807	-0.93281
18	1	0	-1.43086	2.041404	-0.99447
19	1	0	-2.45864	-1.42144	1.392956
20	1	0	-2.57094	1.273232	1.354326
21	18	0	4.884848	0.340229	1.207089

Sum of electronic and zero-point Energies= -947.649466
Sum of electronic and thermal Energies= -947.637370

Sum of electronic and thermal Enthalpies= -947.636426
Sum of electronic and thermal Free Energies= -947.694140

Py_2^+(a)Ar_2(I)

1	7	0	0.313241	-0.00161	1.421467
2	1	0	1.291195	-0.00406	1.184663
3	6	0	-0.45628	1.112174	1.564873
4	6	0	-0.46126	-1.11173	1.56637
5	6	0	-1.76466	0.699465	1.848609
6	6	0	-1.76776	-0.69281	1.849638
7	1	0	-0.03465	2.099551	1.496503
8	1	0	-0.04412	-2.10108	1.498972
9	1	0	-2.59058	1.357471	2.054605
10	1	0	-2.59664	-1.3468	2.056493
11	7	0	-2.96362	0.0043	-1.14338
12	1	0	-3.95513	0.009113	-0.97342
13	6	0	-2.18159	1.113593	-1.27018
14	6	0	-2.19204	-1.11238	-1.26759
15	6	0	-0.87036	0.691104	-1.51236
16	6	0	-0.87679	-0.70262	-1.51069
17	1	0	-2.59728	2.104911	-1.2219
18	1	0	-2.61688	-2.09971	-1.21691
19	1	0	-0.03194	1.341415	-1.68839
20	1	0	-0.04467	-1.36119	-1.68594
21	18	0	2.643952	1.914554	-0.3087
22	18	0	2.644809	-1.91451	-0.30593

Sum of electronic and zero-point Energies= -1475.216471
Sum of electronic and thermal Energies= -1475.201955
Sum of electronic and thermal Enthalpies= -1475.201011
Sum of electronic and thermal Free Energies= -1475.266535

Py_2^+(a)Ar_2(II)

1	7	0	0.003855	-0.03213	-0.08082
2	1	0	0.008219	-0.05316	0.925199
3	6	0	1.113093	0.000237	-0.87095
4	6	0	-1.11144	0.03277	-0.86063
5	6	0	0.691474	0.053432	-2.20487
6	6	0	-0.70096	0.073599	-2.19831
7	1	0	2.103688	-0.05406	-0.45428
8	1	0	-2.09922	0.007281	-0.43466
9	1	0	1.341569	0.049451	-3.06173
10	1	0	-1.35888	0.088553	-3.04907
11	7	0	0.035044	3.220811	-2.70885
12	1	0	0.028429	3.241994	-3.71486
13	6	0	-1.0725	3.189079	-1.91613
14	6	0	1.151986	3.155495	-1.93165
15	6	0	-0.64791	3.135944	-0.58324
16	6	0	0.744494	3.114997	-0.59295
17	1	0	-2.06399	3.243942	-2.3306
18	1	0	2.138835	3.180447	-2.35981
19	1	0	-1.29604	3.140199	0.275104
20	1	0	1.404366	3.099946	0.2563

21	18	0	0.035057	1.946018	2.882041
22	18	0	0.014053	1.242164	-5.67148

Sum of electronic and zero-point Energies= -1475.216280
Sum of electronic and thermal Energies= -1475.201755
Sum of electronic and thermal Enthalpies= -1475.200811
Sum of electronic and thermal Free Energies= -1475.266400

Py_2^+(a)Ar_2(III)

1	7	0	2.532162	0.485491	-0.11285
2	1	0	2.972415	-0.41285	-0.22071
3	6	0	2.187308	1.051513	1.07661
4	6	0	2.124296	1.289323	-1.13423
5	6	0	1.568504	2.278658	0.807502
6	6	0	1.529055	2.427267	-0.57667
7	1	0	2.42984	0.583594	2.014689
8	1	0	2.311158	1.033046	-2.16247
9	1	0	1.224764	2.975151	1.551969
10	1	0	1.147767	3.26356	-1.13591
11	7	0	-1.53199	1.408474	0.094619
12	1	0	-2.0302	2.27809	0.181494
13	6	0	-1.16679	0.825104	-1.08197
14	6	0	-1.12182	0.630467	1.13423
15	6	0	-0.52307	-0.38042	-0.78383
16	6	0	-0.49375	-0.5024	0.603499
17	1	0	-1.41574	1.26683	-2.03095
18	1	0	-1.32936	0.89825	2.155497
19	1	0	-0.15437	-1.08252	-1.51062
20	1	0	-0.09754	-1.31915	1.180312
21	18	0	2.228679	-3.03509	0.000964
22	18	0	-4.26659	-0.76818	-0.01022

Sum of electronic and zero-point Energies= -1475.215244
Sum of electronic and thermal Energies= -1475.200639
Sum of electronic and thermal Enthalpies= -1475.199695
Sum of electronic and thermal Free Energies= -1475.267493

Py_2^+(a)Ar_2(IV)

1	7	0	0.760121	0.178457	0.492768
2	1	0	1.006888	1.050591	0.929402
3	6	0	0.5824	0.000198	-0.84514
4	6	0	0.523503	-0.99378	1.143538
5	6	0	0.249136	-1.34263	-1.06107
6	6	0	0.210279	-1.96463	0.183937
7	1	0	0.738252	0.802419	-1.54451
8	1	0	0.627426	-1.07737	2.211255
9	1	0	0.090151	-1.79364	-2.02475
10	1	0	0.013545	-3.00131	0.393721
11	7	0	-2.98201	-1.43057	-0.47088
12	1	0	-3.25315	-2.29213	-0.91392
13	6	0	-2.80746	-1.25605	0.869769
14	6	0	-2.71674	-0.26168	-1.11973
15	6	0	-2.44674	0.077531	1.087468

16	6	0	-2.38963	0.700553	-0.15787
17	1	0	-2.98355	-2.0541	1.569747
18	1	0	-2.81225	-0.17548	-2.18806
19	1	0	-2.28058	0.524357	2.051894
20	1	0	-2.16699	1.732512	-0.36459
21	18	0	0.447038	3.584843	-0.00453
22	18	0	3.961016	-1.06864	-0.0443

Sum of electronic and zero-point Energies= -1475.215179
Sum of electronic and thermal Energies= -1475.200611
Sum of electronic and thermal Enthalpies= -1475.199666
Sum of electronic and thermal Free Energies= -1475.266035

Py_2^+(a)Ar_2(V)

1	7	0	0.133004	-1.55418	1.412896
2	1	0	0.14666	-1.56514	2.418726
3	6	0	1.232021	-1.46768	0.612464
4	6	0	-0.98674	-1.63216	0.640881
5	6	0	0.801351	-1.51644	-0.71815
6	6	0	-0.58747	-1.61969	-0.70044
7	1	0	2.225779	-1.42058	1.022294
8	1	0	-1.96594	-1.73093	1.075836
9	1	0	1.447604	-1.50942	-1.57818
10	1	0	-1.24936	-1.70929	-1.54376
11	7	0	-0.133	1.554183	-1.4129
12	1	0	-0.14666	1.565142	-2.41873
13	6	0	-1.23202	1.467683	-0.61246
14	6	0	0.98674	1.63216	-0.64088
15	6	0	-0.80135	1.516443	0.718148
16	6	0	0.587469	1.619694	0.700437
17	1	0	-2.22578	1.42058	-1.02229
18	1	0	1.965938	1.730925	-1.07584
19	1	0	-1.4476	1.509418	1.578175
20	1	0	1.249362	1.709289	1.543761
21	18	0	0.285724	-5.00284	0.870335
22	18	0	-0.28572	5.002843	-0.87034

Sum of electronic and zero-point Energies= -1475.214122
Sum of electronic and thermal Energies= -1475.199481
Sum of electronic and thermal Enthalpies= -1475.198536
Sum of electronic and thermal Free Energies= -1475.265615

Py_2^+(a)Ar_3(I)

1	7	0	0.882646	0.093335	-1.3763
2	1	0	1.885585	0.071293	-1.29919
3	6	0	0.080552	-1.00058	-1.49002
4	6	0	0.114581	1.215687	-1.30607
5	6	0	-1.24813	-0.55948	-1.53009
6	6	0	-1.22661	0.828169	-1.41518
7	1	0	0.489787	-1.99228	-1.56959
8	1	0	0.555018	2.193481	-1.22177
9	1	0	-2.10885	-1.19239	-1.65492
10	1	0	-2.06716	1.499173	-1.43215

11	7	0	-1.93724	-0.10521	1.639778
12	1	0	-2.94114	-0.08107	1.57692
13	6	0	-1.17243	-1.23012	1.562216
14	6	0	-1.13001	0.986993	1.742837
15	6	0	0.170158	-0.84604	1.654413
16	6	0	0.1969	0.542172	1.76741
17	1	0	-1.61499	-2.20807	1.48819
18	1	0	-1.53409	1.98042	1.829539
19	1	0	1.008962	-1.51921	1.65949
20	1	0	1.060787	1.173342	1.877794
21	18	0	3.453434	-1.91615	-0.15886
22	18	0	3.457314	1.916082	0.090564
23	18	0	-4.80307	0.029933	-0.43236

Sum of electronic and zero-point Energies= -2002.779282
Sum of electronic and thermal Energies= -2002.762209
Sum of electronic and thermal Enthalpies= -2002.761265
Sum of electronic and thermal Free Energies= -2002.835712

Py_2^+(a)Ar_3(II)

1	7	0	-0.73633	-0.08396	0.005185
2	1	0	-0.95218	0.898697	0.007252
3	6	0	-0.53977	-0.84132	-1.10812
4	6	0	-0.53518	-0.84491	1.115122
5	6	0	-0.23387	-2.14467	-0.69522
6	6	0	-0.23091	-2.14694	0.696662
7	1	0	-0.66343	-0.43239	-2.09532
8	1	0	-0.65474	-0.4393	2.104207
9	1	0	-0.06759	-2.97962	-1.35298
10	1	0	-0.06184	-2.98402	1.350994
11	7	0	2.985594	-1.92962	-0.00879
12	1	0	3.257069	-2.89825	-0.01279
13	6	0	2.761889	-1.17051	-1.11876
14	6	0	2.767668	-1.17813	1.107387
15	6	0	2.417963	0.117969	-0.69734
16	6	0	2.421537	0.113215	0.696529
17	1	0	2.894976	-1.56806	-2.10973
18	1	0	2.905692	-1.58243	2.094948
19	1	0	2.216299	0.950103	-1.34834
20	1	0	2.223291	0.940891	1.354213
21	18	0	-0.25428	2.739587	-1.94398
22	18	0	-0.23376	2.744846	1.941957
23	18	0	-3.94631	-1.44215	0.005092

Sum of electronic and zero-point Energies= -2002.778351
Sum of electronic and thermal Energies= -2002.761184
Sum of electronic and thermal Enthalpies= -2002.760240
Sum of electronic and thermal Free Energies= -2002.836019

Py_2^+(a)Ar_3(III)

1	7	0	-1.87057	1.153739	-0.00812
2	1	0	-2.47944	0.352713	-0.00128
3	6	0	-1.36992	1.767869	1.098609

4	6	0	-1.36038	1.741778	-1.12517
5	6	0	-0.53678	2.810993	0.673984
6	6	0	-0.53108	2.794607	-0.71835
7	1	0	-1.65573	1.46173	2.089545
8	1	0	-1.63811	1.412207	-2.11085
9	1	0	-0.02982	3.50243	1.32397
10	1	0	-0.0185	3.470512	-1.38015
11	7	0	2.276942	1.227243	-0.00419
12	1	0	2.940857	1.9827	-0.02105
13	6	0	1.761093	0.658791	1.12125
14	6	0	1.754555	0.614524	-1.10365
15	6	0	0.898209	-0.36995	0.727841
16	6	0	0.894538	-0.39769	-0.66511
17	1	0	2.059812	0.980999	2.103416
18	1	0	2.048364	0.897257	-2.09935
19	1	0	0.367612	-1.02377	1.397009
20	1	0	0.360039	-1.07732	-1.3048
21	18	0	-2.62005	-1.62089	1.952443
22	18	0	-2.60878	-1.63732	-1.94735
23	18	0	4.458765	-1.53912	-0.00324

Sum of electronic and zero-point Energies= -2002.778368
Sum of electronic and thermal Energies= -2002.761122
Sum of electronic and thermal Enthalpies= -2002.760178
Sum of electronic and thermal Free Energies= -2002.837707

Py_2^+(a)Ar_3(IV)

1	7	0	1.515942	0.049808	0.000728
2	1	0	2.373716	-0.4772	0.00005
3	6	0	0.84358	0.458171	-1.11056
4	6	0	0.842866	0.454134	1.113095
5	6	0	-0.28651	1.172086	-0.69401
6	6	0	-0.28692	1.169586	0.69843
7	1	0	1.217228	0.259964	-2.0999
8	1	0	1.215929	0.252346	2.101928
9	1	0	-0.99141	1.652979	-1.34914
10	1	0	-0.99243	1.647826	1.354838
11	7	0	-2.47714	-1.16032	-0.00192
12	1	0	-3.32877	-0.62323	-0.00086
13	6	0	-1.80278	-1.56287	-1.11491
14	6	0	-1.80278	-1.56723	1.109501
15	6	0	-0.67367	-2.27899	-0.70061
16	6	0	-0.67367	-2.28171	0.692383
17	1	0	-2.17405	-1.35751	-2.10376
18	1	0	-2.1743	-1.36617	2.099146
19	1	0	0.032224	-2.75657	-1.35714
20	1	0	0.032228	-2.76186	1.347029
21	18	0	4.491006	-1.95306	0.002285
22	18	0	-5.41784	0.896786	0.002447
23	18	0	2.846683	3.274271	-0.00161

Sum of electronic and zero-point Energies= -2002.778368
Sum of electronic and thermal Energies= -2002.760367
Sum of electronic and thermal Enthalpies= -2002.759423

Sum of electronic and thermal Free Energies= -2002.836288

Py_2^+(a)Ar_3(V)

1	7	0	1.737884	0.459581	0.004189
2	1	0	1.889361	1.454213	0.003743
3	6	0	1.592638	-0.31211	1.116524
4	6	0	1.59552	-0.31351	-1.10743
5	6	0	1.381059	-1.63247	0.701262
6	6	0	1.382859	-1.63339	-0.69101
7	1	0	1.686576	0.101131	2.105473
8	1	0	1.692113	0.098365	-2.09669
9	1	0	1.273663	-2.47808	1.357601
10	1	0	1.27722	-2.47984	-1.34654
11	7	0	-1.83805	-1.70668	0.001754
12	1	0	-2.03958	-2.69222	0.002703
13	6	0	-1.68374	-0.93438	1.113615
14	6	0	-1.68038	-0.93728	-1.11166
15	6	0	-1.44341	0.379095	0.69615
16	6	0	-1.4414	0.377306	-0.69687
17	1	0	-1.79144	-1.3429	2.103151
18	1	0	-1.78481	-1.34835	-2.10049
19	1	0	-1.31714	1.22414	1.349406
20	1	0	-1.31325	1.220635	-1.35198
21	18	0	0.236495	3.664646	-0.00009
22	18	0	-5.10634	-0.43366	-0.00456
23	18	0	5.031482	-0.73026	-0.00598

Sum of electronic and zero-point Energies= -2002.777101
Sum of electronic and thermal Energies= -2002.759864
Sum of electronic and thermal Enthalpies= -2002.758919
Sum of electronic and thermal Free Energies= -2002.835556

Py_2^+(a)Ar_4(I)

1	7	0	-1.49675	-0.095	1.402111
2	1	0	-2.49077	-0.06274	1.250263
3	6	0	-0.75072	-1.2299	1.479496
4	6	0	-0.68016	0.993364	1.481248
5	6	0	0.588462	-0.85531	1.650357
6	6	0	0.631337	0.536975	1.652823
7	1	0	-1.20377	-2.20511	1.445702
8	1	0	-1.07406	1.993801	1.449961
9	1	0	1.411586	-1.53578	1.777665
10	1	0	1.495295	1.164883	1.780915
11	7	0	1.496596	-0.09751	-1.40372
12	1	0	2.490581	-0.06577	-1.25156
13	6	0	0.749646	-1.23199	-1.4788
14	6	0	0.680882	0.991305	-1.48504
15	6	0	-0.58921	-0.85671	-1.65047
16	6	0	-0.63101	0.535579	-1.65575
17	1	0	1.201918	-2.2075	-1.44312
18	1	0	1.075534	1.991508	-1.45578
19	1	0	-1.41288	-1.53681	-1.77623
20	1	0	-1.49442	1.163998	-1.78504
21	18	0	-4.03357	-1.78618	-0.31575

22	18	0	-3.89297	2.044083	0.092663
23	18	0	4.037202	-1.78291	0.318289
24	18	0	3.889714	2.044305	-0.09213

Sum of electronic and zero-point Energies= -2530.342250
Sum of electronic and thermal Energies= -2530.322596
Sum of electronic and thermal Enthalpies= -2530.321651
Sum of electronic and thermal Free Energies= -2530.404288

Py_2^+(a)Ar_4(II)

1	7	0	1.607856	-1.61213	-0.05876
2	1	0	2.546734	-1.25026	-0.06004
3	6	0	0.869326	-1.87466	1.054784
4	6	0	0.851831	-1.8351	-1.16897
5	6	0	-0.39324	-2.31635	0.641087
6	6	0	-0.4043	-2.29178	-0.75118
7	1	0	1.284	-1.76958	2.041779
8	1	0	1.25078	-1.69513	-2.15818
9	1	0	-1.18436	-2.63577	1.296002
10	1	0	-1.20558	-2.58867	-1.4044
11	7	0	-1.97827	0.475694	0.008241
12	1	0	-2.92767	0.142856	0.013622
13	6	0	-1.21695	0.69619	1.115754
14	6	0	-1.24096	0.728686	-1.10827
15	6	0	0.042672	1.135666	0.692862
16	6	0	0.027778	1.155919	-0.69972
17	1	0	-1.61527	0.570798	2.107313
18	1	0	-1.66034	0.632047	-2.09435
19	1	0	0.849047	1.424901	1.343251
20	1	0	0.820066	1.464544	-1.35842
21	18	0	3.692255	0.29684	1.957559
22	18	0	3.695192	0.460269	-1.90178
23	18	0	-4.08471	-2.376	0.012577
24	18	0	-2.56838	3.911552	0.041027

Sum of electronic and zero-point Energies= -2530.341110
Sum of electronic and thermal Energies= -2530.322296
Sum of electronic and thermal Enthalpies= -2530.321352
Sum of electronic and thermal Free Energies= -2530.402153

Py_2^+(a)Ar_4(III)

1	7	0	-1.57451	-1.64042	-0.01009
2	1	0	-2.51579	-1.285	-0.01088
3	6	0	-0.82421	-1.87631	-1.12144
4	6	0	-0.82687	-1.87869	1.102777
5	6	0	0.437929	-2.31689	-0.70456
6	6	0	0.436142	-2.31847	0.688109
7	1	0	-1.23068	-1.75588	-2.11013
8	1	0	-1.23601	-1.76038	2.0906
9	1	0	1.237342	-2.61808	-1.35819
10	1	0	1.233945	-2.6211	1.343036
11	7	0	1.968014	0.48747	0.000098
12	1	0	2.918313	0.157271	0.003787

13	6	0	1.221593	0.719362	-1.11497
14	6	0	1.212734	0.718972	1.109368
15	6	0	-0.04688	1.145102	-0.70411
16	6	0	-0.0524	1.144895	0.688646
17	1	0	1.635026	0.611101	-2.10234
18	1	0	1.61834	0.610491	2.09995
19	1	0	-0.84587	1.4381	-1.36182
20	1	0	-0.85657	1.437608	1.340148
21	18	0	-3.68104	0.346374	-1.92803
22	18	0	-3.67666	0.31851	1.941177
23	18	0	4.142279	-2.30339	0.004288
24	18	0	2.434265	3.962323	0.008835

Sum of electronic and zero-point Energies= -2530.341168
Sum of electronic and thermal Energies= -2530.321425
Sum of electronic and thermal Enthalpies= -2530.320481
Sum of electronic and thermal Free Energies= -2530.405424

Py_2^+(a)Ar_4(IV)

1	7	0	-1.86498	-0.90064	-0.00697
2	1	0	-2.10883	-1.8767	-0.00772
3	6	0	-1.65081	-0.14572	1.105803
4	6	0	-1.65054	-0.14379	-1.11836
5	6	0	-1.31753	1.149105	0.691157
6	6	0	-1.31733	1.150316	-0.70137
7	1	0	-1.78361	-0.5488	2.094469
8	1	0	-1.78357	-0.54498	-2.10777
9	1	0	-1.12881	1.981222	1.346042
10	1	0	-1.12842	1.983485	-1.3549
11	7	0	1.865381	0.9009	-0.0044
12	1	0	2.10898	1.877018	-0.00335
13	6	0	1.650824	0.144017	1.106951
14	6	0	1.651657	0.145912	-1.11721
15	6	0	1.317973	-1.15015	0.689922
16	6	0	1.318529	-1.14898	-0.70261
17	1	0	1.782945	0.545446	2.096383
18	1	0	1.785136	0.548842	-2.10585
19	1	0	1.129177	-1.98343	1.343302
20	1	0	1.130225	-1.98106	-1.3577
21	18	0	-0.72137	-4.24399	0.02339
22	18	0	-5.04328	0.583205	-0.01181
23	18	0	0.719365	4.242665	0.023363
24	18	0	5.044015	-0.58228	-0.01211

Sum of electronic and zero-point Energies= -2530.339968
Sum of electronic and thermal Energies= -2530.320216
Sum of electronic and thermal Enthalpies= -2530.319272
Sum of electronic and thermal Free Energies= -2530.404455

Py_2^+(a)Ar_5(I)

1	7	0	1.471531	0.616759	-0.05058
2	1	0	2.431302	0.315528	-0.05808
3	6	0	0.696282	0.792151	-1.1554

4	6	0	0.73114	0.850411	1.067299
5	6	0	-0.57891	1.183927	-0.72986
6	6	0	-0.55695	1.220672	0.662
7	1	0	1.094579	0.668611	-2.14697
8	1	0	1.160742	0.779115	2.050803
9	1	0	-1.40084	1.430101	-1.37845
10	1	0	-1.35828	1.501584	1.322169
11	7	0	-1.97223	-1.64763	0.074118
12	1	0	-2.92891	-1.33627	0.081622
13	6	0	-1.23145	-1.88097	-1.04451
14	6	0	-1.19422	-1.81739	1.17875
15	6	0	0.056935	-2.24908	-0.63975
16	6	0	0.080404	-2.20921	0.752522
17	1	0	-1.66116	-1.80874	-2.028
18	1	0	-1.59046	-1.68863	2.170557
19	1	0	0.858281	-2.52977	-1.30007
20	1	0	0.903659	-2.45245	1.400605
21	18	0	3.676904	-1.33107	-1.8947
22	18	0	3.68303	-1.14771	1.957207
23	18	0	-4.16965	0.155371	-1.92828
24	18	0	-4.16531	0.328741	1.915203
25	18	0	1.973724	4.049879	-0.09529

Sum of electronic and zero-point Energies= -3057.904109
Sum of electronic and thermal Energies= -3057.881858
Sum of electronic and thermal Enthalpies= -3057.880914
Sum of electronic and thermal Free Energies= -3057.973596

Py_2^+(a)Ar_5(II)

1	7	0	-1.695	0.062957	0.002435
2	1	0	-2.25421	-0.77321	0.00154
3	6	0	-1.2368	0.699038	1.114924
4	6	0	-1.23729	0.701656	-1.10872
5	6	0	-0.48366	1.804468	0.700383
6	6	0	-0.48397	1.806126	-0.69189
7	1	0	-1.49645	0.36146	2.102689
8	1	0	-1.49747	0.366502	-2.09717
9	1	0	-0.02198	2.521842	1.355469
10	1	0	-0.02259	2.525079	-1.34543
11	7	0	2.416091	0.46451	0.002287
12	1	0	2.983562	1.295183	0.003722
13	6	0	1.951943	-0.17144	1.113401
14	6	0	1.951875	-0.16748	-1.11101
15	6	0	1.189319	-1.26803	0.695747
16	6	0	1.189247	-1.26554	-0.69717
17	1	0	2.21642	0.157807	2.10293
18	1	0	2.216229	0.165298	-2.09939
19	1	0	0.721204	-1.98286	1.349076
20	1	0	0.721116	-1.97805	-1.35302
21	18	0	-2.27462	-2.73972	1.971379
22	18	0	-2.27281	-2.73848	-1.97016
23	18	0	2.481251	4.003668	0.001854
24	18	0	4.828227	-2.08685	-0.00485
25	18	0	-4.18746	2.495599	-0.00641

Sum of electronic and zero-point Energies= -3057.903153
Sum of electronic and thermal Energies= -3057.880749
Sum of electronic and thermal Enthalpies= -3057.879805
Sum of electronic and thermal Free Energies= -3057.975478

Py_2^+cc1

1	6	0	2.83129	-0.78069	0.000146
2	6	0	1.50288	-1.18198	0.000213
3	6	0	0.693492	-0.03665	0.000185
4	6	0	1.608899	1.165668	0.000361
5	7	0	2.930866	0.55009	0.000349
6	6	0	-0.69349	0.036647	-4.1E-05
7	6	0	-1.6089	-1.16567	0.000031
8	7	0	-2.93087	-0.55009	-0.00028
9	6	0	-2.83129	0.780687	-0.00051
10	6	0	-1.50288	1.181979	-0.00048
11	1	0	3.712251	-1.40364	0.000073
12	1	0	1.175524	-2.20746	0.000148
13	1	0	1.47563	1.794271	0.884825
14	1	0	1.475748	1.79448	-0.88397
15	1	0	3.79389	1.065975	0.000468
16	1	0	-1.4758	-1.79421	0.884567
17	1	0	-1.47558	-1.79454	-0.88423
18	1	0	-3.79389	-1.06598	-0.00032
19	1	0	-3.71225	1.40364	-0.00081
20	1	0	-1.17552	2.207458	-0.00072

Sum of electronic and zero-point Energies= -420.113995
Sum of electronic and thermal Energies= -420.105423
Sum of electronic and thermal Enthalpies= -420.104479
Sum of electronic and thermal Free Energies= -420.148139

Py_2^+cn1

1	6	0	-2.75919	0.760151	-0.00014
2	6	0	-1.44171	1.192935	0.000242
3	6	0	-0.63469	0.053789	0.000165
4	6	0	-1.51529	-1.16751	0.000061
5	7	0	-2.84187	-0.57251	-9.1E-05
6	7	0	0.697237	-0.01349	0.000095
7	6	0	1.576712	1.181753	-0.00032
8	6	0	2.943471	0.579585	0.000188
9	6	0	2.836343	-0.77964	-7.9E-05
10	6	0	1.48065	-1.14381	-0.00003
11	1	0	-3.64955	1.369692	-0.00019
12	1	0	-1.12628	2.220755	0.000566
13	1	0	-1.35736	-1.79007	-0.886
14	1	0	-1.35758	-1.79011	0.886129
15	1	0	-3.69572	-1.10278	-0.00021
16	1	0	1.371057	1.789157	0.88463
17	1	0	1.37124	1.788521	-0.88574
18	1	0	3.845957	1.166817	0.000337
19	1	0	3.649551	-1.48653	-6E-06
20	1	0	1.043401	-2.12692	-3.2E-05

Sum of electronic and zero-point Energies= -420.092486
Sum of electronic and thermal Energies= -420.084085
Sum of electronic and thermal Enthalpies= -420.083140
Sum of electronic and thermal Free Energies= -420.126291

Py(CC1)$^+$

1	6	0	1.188254	-0.26863	0.066
2	6	0	0.509093	1.05262	-0.0051
3	6	0	-0.85573	0.860013	0.001967
4	6	0	-1.07351	-0.55268	0.059575
5	7	0	0.057553	-1.16894	-0.14705
6	1	0	1.623519	-0.48335	1.048417
7	1	0	1.972289	-0.40166	-0.68299
8	1	0	1.026337	1.990722	-0.15242
9	1	0	-1.62349	1.612603	-0.09377
10	1	0	-2.0102	-1.0837	0.175496

Sum of electronic and zero-point Energies= -209.810083
Sum of electronic and thermal Energies= -209.806038
Sum of electronic and thermal Enthalpies= -209.805094
Sum of electronic and thermal Free Energies= -209.837244

Py(CC2)$^+$

1	6	0	1.103705	0.46259	0.000031
2	6	0	-0.11446	1.186121	-0.00002
3	6	0	-1.20105	0.183742	0.000032
4	6	0	-0.40228	-1.07765	-5.8E-05
5	7	0	0.906669	-0.87366	-4.1E-05
6	1	0	2.103273	0.876079	0.000246
7	1	0	-0.22608	2.261072	-0.00033
8	1	0	-1.86597	0.266078	0.870389
9	1	0	-1.86633	0.266125	-0.87
10	1	0	-0.80708	-2.08257	0.000072

Sum of electronic and zero-point Energies= -209.816858
Sum of electronic and thermal Energies= -209.812541
Sum of electronic and thermal Enthalpies= -209.811597
Sum of electronic and thermal Free Energies= -209.844294

Py(OC1)$^+$

1	6	0	2.248555	-0.82374	0.073896
2	1	0	2.552851	-0.27551	-0.83412
3	1	0	2.580616	-1.85459	0.020997
4	1	0	1.147295	-0.77245	0.026616
5	6	0	2.722058	-0.12066	1.25304
6	1	0	3.347445	-0.63519	1.977272
7	6	0	2.406544	1.224297	1.514269
8	1	0	1.786025	1.798404	0.832385
9	6	0	2.879784	1.855282	2.651365
10	7	0	3.281673	2.384551	3.613219

Sum of electronic and zero-point Energies= -209.798669
Sum of electronic and thermal Energies= -209.793361
Sum of electronic and thermal Enthalpies= -209.792417
Sum of electronic and thermal Free Energies= -209.827457

Py(OC2)$^+$

1	6	0	0.96086	1.330749	0.369805
2	6	0	0.763691	0.02205	0.839731
3	6	0	-0.37962	-0.82571	0.512402
4	6	0	-1.3927	-0.27883	-0.36533
5	7	0	-2.17119	0.197084	-1.06544
6	1	0	1.840211	1.890562	0.663635
7	1	0	0.239518	1.799895	-0.29147
8	1	0	1.508471	-0.41218	1.499466
9	1	0	0.011667	-1.78612	0.117573
10	1	0	-0.82631	-1.1773	1.465229

Sum of electronic and zero-point Energies= -209.782078
Sum of electronic and thermal Energies= -209.776048
Sum of electronic and thermal Enthalpies= -209.775104
Sum of electronic and thermal Free Energies= -209.811895

Py(OC3)$^+$

1	7	0	2.401065	-0.17652	-0.09867
2	6	0	1.291725	0.129792	-0.07423
3	6	0	-0.0317	0.517011	-0.02721
4	6	0	-1.07611	-0.41016	-0.01836
5	6	0	-2.39451	-0.0224	0.028347
6	1	0	3.367156	-0.43794	-0.18816
7	1	0	-0.20789	1.586531	0.00176
8	1	0	-0.83755	-1.46621	-0.04887
9	1	0	-3.18626	-0.75729	0.034832
10	1	0	-2.67604	1.02198	0.059914

Sum of electronic and zero-point Energies= -209.842819
Sum of electronic and thermal Energies= -209.836577
Sum of electronic and thermal Enthalpies= -209.835633
Sum of electronic and thermal Free Energies= -209.872881

$Py_2(CC1)^+(I)$

1	6	0	-2.04118	-1.02599	-0.3327
2	6	0	-3.45977	-0.733	-0.23219
3	6	0	-3.55547	0.54258	0.178434
4	6	0	-2.17631	1.080265	0.345235
5	7	0	-1.28723	-0.02523	-0.01401
6	1	0	-1.61609	-1.97394	-0.63967
7	1	0	-4.25781	-1.42335	-0.45068
8	1	0	-4.45452	1.108428	0.364394
9	1	0	-1.98175	1.942603	-0.30014
10	1	0	-1.97444	1.40596	1.370495

11	6	0	2.161029	-0.34275	1.03552
12	6	0	3.539529	-0.21163	0.655836
13	6	0	3.547955	0.213037	-0.6409
14	6	0	2.174527	0.337198	-1.04076
15	7	0	1.37204	-0.00427	-0.00825
16	1	0	0.261004	-0.00774	-0.01481
17	1	0	1.741293	-0.65464	1.979078
18	1	0	4.381447	-0.41678	1.29467
19	1	0	4.398105	0.422297	-1.26739
20	1	0	1.767232	0.645374	-1.99096

Sum of electronic and zero-point Energies= -420.069274
Sum of electronic and thermal Energies= -420.059789
Sum of electronic and thermal Enthalpies= -420.058845
Sum of electronic and thermal Free Energies= -420.107255

$Py_2(CC1)^+$(II)

1	6	0	1.90901	1.072352	-0.02459
2	6	0	3.121474	0.281908	0.130194
3	6	0	2.748863	-1.00739	0.0645
4	6	0	1.270087	-1.05868	-0.13908
5	7	0	0.863159	0.34003	-0.19349
6	1	0	1.851443	2.153928	-0.00675
7	1	0	4.112539	0.683079	0.265825
8	1	0	3.379683	-1.87963	0.132691
9	1	0	0.99245	-1.57134	-1.06468
10	1	0	0.748218	-1.56609	0.677252
11	6	0	-1.29356	1.044383	-0.16508
12	6	0	-1.65984	0.545403	1.123381
13	6	0	-2.44064	-0.57482	0.928355
14	6	0	-2.58997	-0.74545	-0.45774
15	7	0	-1.91214	0.253619	-1.08893
16	1	0	-1.86628	0.387035	-2.08565
17	1	0	-0.90814	2.011943	-0.42908
18	1	0	-1.34796	0.979289	2.057426
19	1	0	-2.8766	-1.20828	1.681153
20	1	0	-3.13508	-1.49169	-1.01088

Sum of electronic and zero-point Energies= -420.057484
Sum of electronic and thermal Energies= -420.047978
Sum of electronic and thermal Enthalpies= -420.047034
Sum of electronic and thermal Free Energies= -420.095304

$Py_2(CC1)^+$(III)

1	6	0	3.057887	0.27692	2.034412
2	6	0	3.297046	1.667672	1.669805
3	6	0	2.287363	2.023647	0.860577
4	6	0	1.389902	0.84172	0.706795
5	7	0	1.990892	-0.22494	1.509907
6	1	0	3.695512	-0.31105	2.684938
7	1	0	4.128665	2.269357	1.99917
8	1	0	2.125063	2.980986	0.391146
9	1	0	0.372111	1.041589	1.056959

10	1	0	1.3004	0.517943	-0.33515
11	6	0	0.890267	-2.99009	2.087652
12	6	0	-0.0212	-3.44829	3.098225
13	6	0	-0.19715	-4.79435	2.923059
14	6	0	0.600303	-5.17674	1.807465
15	7	0	1.233552	-4.06636	1.337743
16	1	0	1.865229	-4.0408	0.551044
17	1	0	1.291749	-1.98143	1.876905
18	1	0	-0.46726	-2.81496	3.846115
19	1	0	-0.81142	-5.46583	3.498292
20	1	0	0.736004	-6.14507	1.352421

Sum of electronic and zero-point Energies= -420.050623
Sum of electronic and thermal Energies= -420.040708
Sum of electronic and thermal Enthalpies= -420.039764
Sum of electronic and thermal Free Energies= -420.090832

$Py_2(CC1)^+(IV)$

1	6	0	1.058971	0.003503	0.314872
2	6	0	0.639734	1.265497	-0.28047
3	6	0	-0.68918	1.172969	-0.46365
4	6	0	-1.11969	-0.16969	0.024729
5	7	0	0.093973	-0.83292	0.502183
6	1	0	2.078447	-0.24368	0.587957
7	1	0	1.285567	2.09524	-0.51893
8	1	0	-1.34859	1.915357	-0.88484
9	1	0	-1.8505	-0.10446	0.83712
10	1	0	-1.58579	-0.77185	-0.76173
11	6	0	0.055796	-4.23141	2.864337
12	6	0	0.575642	-3.72636	1.630556
13	6	0	1.357408	-4.72292	1.098239
14	6	0	1.317738	-5.82442	1.990262
15	7	0	0.521112	-5.49111	3.047906
16	1	0	0.313846	-6.08701	3.835056
17	1	0	-0.59618	-3.75851	3.581378
18	1	0	0.37112	-2.73126	1.229363
19	1	0	1.909391	-4.70203	0.174229
20	1	0	1.795204	-6.78929	1.927967

Sum of electronic and zero-point Energies= -420.045946
Sum of electronic and thermal Energies= -420.035883
Sum of electronic and thermal Enthalpies= -420.034938
Sum of electronic and thermal Free Energies= -420.086752

$Py_2(CC2)^+(I)$

1	6	0	0.997838	0.616887	-0.69104
2	6	0	-0.29681	0.950291	-0.69836
3	6	0	-0.99269	0.073154	0.29161
4	6	0	0.140361	-0.7506	0.798623
5	7	0	1.257519	-0.43585	0.237987
6	1	0	1.811194	1.025584	-1.26794
7	1	0	-0.77096	1.707898	-1.29896
8	1	0	-1.47968	0.628753	1.100632

9	1	0	-1.77606	-0.55066	-0.15292
10	1	0	0.070577	-1.52829	1.547065
11	6	0	4.195366	-2.58265	0.021022
12	6	0	5.506263	-2.86211	0.53437
13	6	0	5.745082	-1.94532	1.516821
14	6	0	4.578052	-1.11359	1.595566
15	7	0	3.670398	-1.5277	0.683356
16	1	0	2.683121	-1.08175	0.506351
17	1	0	3.648334	-3.0833	-0.76244
18	1	0	6.152306	-3.65086	0.18868
19	1	0	6.624101	-1.83983	2.129363
20	1	0	4.379477	-0.27631	2.246136

Sum of electronic and zero-point Energies= -420.065054
Sum of electronic and thermal Energies= -420.055493
Sum of electronic and thermal Enthalpies= -420.054549
Sum of electronic and thermal Free Energies= -420.103203

$Py_2(CC2)^+$(II)

1	6	0	0.813286	-0.84986	-1.1111
2	6	0	-0.16697	-0.61677	-1.991
3	6	0	-1.33005	-0.05251	-1.23814
4	6	0	-0.79972	-0.03373	0.158249
5	7	0	0.40216	-0.47991	0.201007
6	1	0	1.799676	-1.25276	-1.26995
7	1	0	-0.14205	-0.79429	-3.05311
8	1	0	-1.61885	0.950275	-1.57149
9	1	0	-2.23452	-0.66602	-1.31265
10	1	0	-1.32888	0.295705	1.041898
11	6	0	1.714698	-0.58587	2.123766
12	6	0	0.695033	-1.01827	3.024416
13	6	0	0.327298	0.070248	3.78644
14	6	0	1.133038	1.152097	3.388507
15	7	0	1.971892	0.723097	2.406153
16	1	0	2.664527	1.289533	1.944577
17	1	0	2.389544	-1.17579	1.531652
18	1	0	0.297204	-2.01741	3.061818
19	1	0	-0.42178	0.107116	4.558244
20	1	0	1.160848	2.166741	3.748784

Sum of electronic and zero-point Energies= -420.054243
Sum of electronic and thermal Energies= -420.044672
Sum of electronic and thermal Enthalpies= -420.043728
Sum of electronic and thermal Free Energies= -420.092147

$Py_2(CC2)^+$(III)

1	6	0	1.291385	0.547501	0.727653
2	6	0	0.270995	1.377371	0.366519
3	6	0	-0.99904	0.655631	0.661039
4	6	0	-0.46111	-0.59882	1.276514
5	7	0	0.835705	-0.65042	1.281282
6	1	0	2.349014	0.701954	0.58003
7	1	0	0.35353	2.340514	-0.11048

8	1	0	-1.69657	1.195679	1.305354
9	1	0	-1.55534	0.4296	-0.25736
10	1	0	-1.06193	-1.41429	1.656299
11	6	0	0.778711	-0.05542	4.20452
12	6	0	-0.22665	0.916428	4.047916
13	6	0	0.376911	2.05277	3.534651
14	6	0	1.749508	1.764363	3.381759
15	7	0	1.959788	0.492949	3.812761
16	1	0	2.840471	0.004447	3.785713
17	1	0	0.721563	-1.0653	4.572381
18	1	0	-1.26107	0.783547	4.312986
19	1	0	-0.08215	3.00003	3.311443
20	1	0	2.5576	2.391164	3.046271

Sum of electronic and zero-point Energies= -420.051421
Sum of electronic and thermal Energies= -420.042006
Sum of electronic and thermal Enthalpies= -420.041062
Sum of electronic and thermal Free Energies= -420.087709

$Py_2(CC2)^+$(IV)

1	6	0	0.425136	0.913349	0.744245
2	6	0	-0.86296	1.048657	0.393946
3	6	0	-1.22151	-0.13701	-0.44075
4	6	0	0.076008	-0.87728	-0.45136
5	7	0	1.000006	-0.27773	0.218877
6	1	0	1.02944	1.571977	1.347414
7	1	0	-1.53049	1.85179	0.657431
8	1	0	-2.03124	-0.73973	-0.01551
9	1	0	-1.54494	0.126376	-1.45368
10	1	0	0.252188	-1.81556	-0.96106
11	6	0	5.747003	-2.07393	1.230999
12	6	0	6.264284	-1.14631	0.28685
13	6	0	5.19635	-0.45249	-0.22202
14	6	0	4.012952	-0.95168	0.409007
15	7	0	4.394121	-1.92597	1.273023
16	1	0	3.764797	-2.4518	1.860626
17	1	0	6.259637	-2.79656	1.845368
18	1	0	7.306445	-1.03541	0.041009
19	1	0	5.20947	0.329822	-0.96153
20	1	0	2.957332	-0.67491	0.293529

Sum of electronic and zero-point Energies= -420.048015
Sum of electronic and thermal Energies= -420.037962
Sum of electronic and thermal Enthalpies= -420.037018
Sum of electronic and thermal Free Energies= -420.088584

$Py_2(CC2)^+$(V)

1	6	0	1.009943	0.670951	0.091135
2	6	0	-0.05191	1.394938	-0.35605
3	6	0	-1.24967	0.508882	-0.26768
4	6	0	-0.63613	-0.71542	0.341094
5	7	0	0.641041	-0.61039	0.521668
6	1	0	2.050245	0.953748	0.08942

7	1	0	-0.03106	2.385171	-0.78179
8	1	0	-2.08921	0.913413	0.305006
9	1	0	-1.65781	0.285156	-1.26116
10	1	0	-1.17346	-1.61871	0.599819
11	6	0	0.238394	-0.09762	3.497553
12	6	0	1.621905	0.131782	3.420901
13	6	0	1.798945	1.421469	2.957266
14	6	0	0.514182	1.975429	2.744635
15	7	0	-0.40438	1.037434	3.091087
16	1	0	-1.40155	1.17453	3.10058
17	1	0	-0.30505	-0.96771	3.82385
18	1	0	2.382245	-0.58718	3.670068
19	1	0	2.72807	1.935599	2.782125
20	1	0	0.226971	2.966386	2.438916

Sum of electronic and zero-point Energies= -420.047470
Sum of electronic and thermal Energies= -420.037905
Sum of electronic and thermal Enthalpies= -420.036961
Sum of electronic and thermal Free Energies= -420.084544

$Py_2(CC2)^+$(VI)

1	6	0	2.415373	0.837291	0.668328
2	6	0	3.731217	0.655046	0.431589
3	6	0	3.85459	-0.52038	-0.4791
4	6	0	2.415941	-0.88821	-0.65418
5	7	0	1.612718	-0.11399	-0.0027
6	1	0	1.946413	1.589404	1.282632
7	1	0	4.550571	1.236976	0.819493
8	1	0	4.333136	-0.28006	-1.43471
9	1	0	4.438402	-1.34202	-0.05033
10	1	0	2.054162	-1.71029	-1.25765
11	6	0	-2.51566	-0.94143	0.580599
12	6	0	-1.65047	0.004127	-0.03594
13	6	0	-2.45516	0.976046	-0.59096
14	6	0	-3.79751	0.627491	-0.3166
15	7	0	-3.79835	-0.53712	0.393789
16	1	0	-4.61975	-1.01858	0.725074
17	1	0	-2.2807	-1.84397	1.120595
18	1	0	-0.56703	-0.05668	-0.04261
19	1	0	-2.14703	1.850069	-1.13825
20	1	0	-4.71532	1.127365	-0.5796

Sum of electronic and zero-point Energies= -420.044284
Sum of electronic and thermal Energies= -420.034117
Sum of electronic and thermal Enthalpies= -420.033173
Sum of electronic and thermal Free Energies= -420.085443

$Py_2(OC1)^+$(I)

1	6	0	-2.12011	0.127009	-0.14516
2	1	0	-2.24987	0.836744	0.669507
3	1	0	-2.51819	0.564672	-1.06406
4	1	0	-2.72526	-0.76026	0.057118
5	6	0	-0.70036	-0.26017	-0.34054

6	1	0	-0.4915	-0.96701	-1.13673
7	6	0	0.320638	0.201766	0.393015
8	1	0	0.172834	0.908627	1.200164
9	6	0	1.658622	-0.20338	0.160931
10	7	0	2.754223	-0.51731	-0.00821
11	6	0	6.030353	-2.24682	0.261704
12	6	0	7.398684	-2.27471	-0.16533
13	6	0	7.560406	-1.23923	-1.04089
14	6	0	6.290313	-0.58242	-1.14549
15	7	0	5.4012	-1.21794	-0.34953
16	1	0	4.393785	-0.95498	-0.22427
17	1	0	5.510744	-2.89582	0.948657
18	1	0	8.133585	-2.9901	0.161727
19	1	0	8.452826	-0.946	-1.56665
20	1	0	6.006215	0.2761	-1.73314

Sum of electronic and zero-point Energies= -420.076278
Sum of electronic and thermal Energies= -420.064945
Sum of electronic and thermal Enthalpies= -420.064001
Sum of electronic and thermal Free Energies= -420.117674

$Py_2(OC1)^+$(II)

1	6	0	-5.11929	-0.11723	0.023279
2	1	0	-5.24011	-1.03222	-0.55344
3	1	0	-5.56272	-0.25967	1.011996
4	1	0	-5.69202	0.681353	-0.45516
5	6	0	-3.69697	0.287064	0.155661
6	1	0	-3.49621	1.191639	0.720052
7	6	0	-2.66362	-0.38339	-0.372
8	1	0	-2.80804	-1.28914	-0.94818
9	6	0	-1.32242	0.050199	-0.21841
10	7	0	-0.22445	0.396042	-0.12548
11	6	0	3.398259	0.262615	-0.85487
12	6	0	3.259909	-1.05127	-0.33689
13	6	0	2.49758	-0.95985	0.797899
14	6	0	2.162915	0.419665	0.973234
15	7	0	2.743379	1.120255	-0.02863
16	1	0	2.625158	2.108989	-0.18616
17	1	0	3.914124	0.609057	-1.73552
18	1	0	3.687364	-1.93521	-0.77749
19	1	0	2.183909	-1.75326	1.453706
20	1	0	1.603555	0.899433	1.756059

Sum of electronic and zero-point Energies= -420.064037
Sum of electronic and thermal Energies= -420.052455
Sum of electronic and thermal Enthalpies= -420.051511
Sum of electronic and thermal Free Energies= -420.106095

$Py_2(OC1)^+$(III)

1	6	0	-1.90566	0.090329	0.22101
2	1	0	-2.15869	0.655134	-0.67453

3	1	0	-2.50289	-0.82455	0.240897
4	1	0	-2.20244	0.669915	1.098551
5	6	0	-0.45791	-0.24055	0.29041
6	1	0	-0.12624	-0.80396	1.15618
7	6	0	0.445378	0.107491	-0.6327
8	1	0	0.165815	0.670074	-1.51517
9	6	0	1.820595	-0.23295	-0.52117
10	7	0	2.941687	-0.49697	-0.45375
11	6	0	7.462426	-0.44153	0.121205
12	6	0	6.053355	-0.53531	-0.08181
13	6	0	5.818218	-1.70407	-0.75362
14	6	0	7.082352	-2.33094	-0.96484
15	7	0	8.049264	-1.54117	-0.42425
16	1	0	9.038806	-1.73841	-0.42743
17	1	0	8.043953	0.325577	0.607762
18	1	0	5.313214	0.179578	0.233392
19	1	0	4.858767	-2.07922	-1.06497
20	1	0	7.322239	-3.26169	-1.45427

Sum of electronic and zero-point Energies= -420.059117
Sum of electronic and thermal Energies= -420.047374
Sum of electronic and thermal Enthalpies= -420.046430
Sum of electronic and thermal Free Energies= -420.101857

$Py_2(OC_2)^+$(I)

1	6	0	-1.2293	1.548045	5.68965
2	6	0	-1.08376	0.419389	6.364538
3	6	0	-0.17735	-0.73405	5.996676
4	6	0	0.584278	-0.52979	4.773334
5	7	0	1.180132	-0.35972	3.80777
6	1	0	-1.90577	2.31555	6.036911
7	1	0	-0.68805	1.753178	4.774624
8	1	0	-1.64196	0.248486	7.276796
9	1	0	0.53633	-0.93103	6.802606
10	1	0	-0.75888	-1.6532	5.875627
11	6	0	3.892829	0.520198	1.39312
12	6	0	4.3061	0.528775	0.02071
13	6	0	3.279104	-0.00408	-0.7051
14	6	0	2.241498	-0.33672	0.226032
15	7	0	2.649487	-0.00625	1.472535
16	1	0	2.097395	-0.1373	2.349692
17	1	0	4.419637	0.855937	2.272275
18	1	0	5.253863	0.89347	-0.33656
19	1	0	3.226691	-0.15834	-1.76922
20	1	0	1.273044	-0.77689	0.048555

Sum of electronic and zero-point Energies= -420.064230
Sum of electronic and thermal Energies= -420.053080
Sum of electronic and thermal Enthalpies= -420.052136
Sum of electronic and thermal Free Energies= -420.106064

$Py_2(OC2)^+$(II)

1	6	0	-3.12395	1.552022	0.331811

2	6	0	-3.74217	0.395407	0.155429
3	6	0	-3.08392	-0.94187	-0.0956
4	6	0	-1.62818	-0.89438	-0.14152
5	7	0	-0.48049	-0.84554	-0.1707
6	1	0	-3.68545	2.458514	0.505997
7	1	0	-2.04506	1.640723	0.310429
8	1	0	-4.8237	0.342259	0.18385
9	1	0	-3.37165	-1.65777	0.680299
10	1	0	-3.43469	-1.36545	-1.04162
11	6	0	2.925744	0.3418	0.978102
12	6	0	2.700764	1.329234	-0.01834
13	6	0	2.202805	0.685758	-1.11937
14	6	0	2.119233	-0.70592	-0.79507
15	7	0	2.579613	-0.86951	0.466516
16	1	0	2.591913	-1.7433	0.969805
17	1	0	3.302913	0.444786	1.982696
18	1	0	2.896477	2.380729	0.102067
19	1	0	1.9109	1.111604	-2.06364
20	1	0	1.798094	-1.53614	-1.39836

Sum of electronic and zero-point Energies= -420.052309
Sum of electronic and thermal Energies= -420.040867
Sum of electronic and thermal Enthalpies= -420.039923
Sum of electronic and thermal Free Energies= -420.095347

$Py_2(OC2)^+$(III)

1	6	0	3.85322	1.136218	-1.03885
2	6	0	4.307947	0.189465	-0.23375
3	6	0	3.477415	-0.68474	0.677629
4	6	0	2.044892	-0.42264	0.619873
5	7	0	0.917214	-0.2077	0.566507
6	1	0	4.530305	1.710866	-1.65433
7	1	0	2.79989	1.374622	-1.11537
8	1	0	5.369134	-0.02194	-0.18152
9	1	0	3.635752	-1.74024	0.43669
10	1	0	3.799246	-0.56151	1.716157
11	6	0	-2.05966	0.365889	0.585636
12	6	0	-2.13728	-0.51026	-0.54462
13	6	0	-3.45305	-0.57962	-0.91656
14	6	0	-4.18614	0.249049	-0.0216
15	7	0	-3.31018	0.800052	0.867453
16	1	0	-3.55576	1.430625	1.616141
17	1	0	-1.18077	0.652766	1.141074
18	1	0	-1.2802	-0.99655	-0.97689
19	1	0	-3.88661	-1.14261	-1.72526
20	1	0	-5.24279	0.460996	0.019902

Sum of electronic and zero-point Energies= -420.051318
Sum of electronic and thermal Energies= -420.039767
Sum of electronic and thermal Enthalpies= -420.038822
Sum of electronic and thermal Free Energies= -420.094870

$Py_2(OC3)^+$(I)

1	7	0	2.326781	0.330631	-0.38412
2	6	0	3.460477	0.48903	-0.20095
3	6	0	4.802322	0.754756	-0.03234
4	6	0	5.797162	0.094799	-0.76483
5	6	0	7.129412	0.362589	-0.59643
6	1	0	1.402354	-0.13063	-0.14255
7	1	0	5.048547	1.50921	0.705737
8	1	0	5.495668	-0.65121	-1.48982
9	1	0	7.877535	-0.15964	-1.17429
10	1	0	7.471419	1.100915	0.116767
11	6	0	-0.28082	-1.23349	-0.69728
12	6	0	-0.34418	-0.57416	0.523655
13	6	0	-0.73425	0.768794	0.258217
14	6	0	-0.87755	0.890212	-1.1024
15	7	0	-0.59897	-0.33019	-1.6679
16	1	0	-0.65251	-0.53498	-2.65129
17	1	0	-0.06825	-2.26243	-0.92897
18	1	0	-0.20033	-1.0309	1.488358
19	1	0	-0.8929	1.549044	0.981705
20	1	0	-1.158	1.733617	-1.708

Sum of electronic and zero-point Energies= -420.048444
Sum of electronic and thermal Energies= -420.037436
Sum of electronic and thermal Enthalpies= -420.036491
Sum of electronic and thermal Free Energies= -420.088551

$Py_2(OC3)^+$(II)

1	7	0	2.528751	1.174426	-1.24375
2	6	0	3.70294	1.035372	-1.15459
3	6	0	5.024634	0.954989	-0.90553
4	6	0	5.621872	-0.18047	-0.29854
5	6	0	6.952668	-0.26783	-0.05185
6	1	0	1.881942	0.941832	-1.98873
7	1	0	5.63102	1.785482	-1.24345
8	1	0	4.972043	-0.99372	0.000877
9	1	0	7.375168	-1.14568	0.413582
10	1	0	7.637456	0.504977	-0.36938
11	6	0	4.758055	2.348655	1.860957
12	6	0	5.950673	2.972989	1.494355
13	6	0	6.997659	2.158689	1.929531
14	6	0	6.428177	1.04453	2.55424
15	7	0	5.072019	1.188948	2.506471
16	1	0	4.407896	0.558963	2.923557
17	1	0	3.737167	2.667422	1.742309
18	1	0	6.027624	3.919332	0.987748
19	1	0	8.053296	2.342592	1.831065
20	1	0	6.889806	0.216855	3.062878

Sum of electronic and zero-point Energies= -420.046453
Sum of electronic and thermal Energies= -420.035666
Sum of electronic and thermal Enthalpies= -420.034722
Sum of electronic and thermal Free Energies= -420.084656

$Py_2(OC_3)^+$(III)

1	7	0	3.462783	-1.95568	-0.82677
2	6	0	4.005564	-0.91019	-0.73703
3	6	0	4.639903	0.280187	-0.80472
4	6	0	6.044974	0.407617	-0.68494
5	6	0	6.689775	1.596651	-0.81348
6	1	0	2.997909	-2.58198	-0.18334
7	1	0	4.019657	1.143387	-1.00976
8	1	0	6.618776	-0.48614	-0.4722
9	1	0	7.76477	1.656165	-0.73364
10	1	0	6.160544	2.499151	-1.08052
11	6	0	6.35788	2.171592	2.001344
12	6	0	5.325461	3.013533	1.580627
13	6	0	4.122944	2.326008	1.766972
14	6	0	4.437136	1.079711	2.304567
15	7	0	5.791788	1.009793	2.443401
16	1	0	6.295179	0.242756	2.85542
17	1	0	7.416354	2.346631	2.075813
18	1	0	5.454364	4.012966	1.203132
19	1	0	3.129127	2.685187	1.563349
20	1	0	3.797061	0.279128	2.631452

Sum of electronic and zero-point Energies= -420.044472
Sum of electronic and thermal Energies= -420.033630
Sum of electronic and thermal Enthalpies= -420.032686
Sum of electronic and thermal Free Energies= -420.082794
